# Enhancing Auxiliary‐Mediated Native Chemical Ligation at Challenging Junctions with Pyridine Scaffolds

**DOI:** 10.1002/chem.202202065

**Published:** 2022-10-17

**Authors:** Sebastian Trunschke, Emanuele Piemontese, Olaf Fuchs, Skander Abboud, Oliver Seitz

**Affiliations:** ^1^ Department of Chemistry Humboldt-Universität zu Berlin Brook-Taylor-Str. 2 12489 Berlin Germany

**Keywords:** base catalysis, ligation auxiliaries, peptide ligation, proteins, protein synthesis

## Abstract

To expand the scope of native chemical ligation (NCL) beyond reactions at cysteine, ligation auxiliaries are appended to the peptide N‐terminus. After the introduction of a pyridine‐containing auxiliary, which provided access to challenging junctions (proline or β‐branched amino acids), we herein probe the role of the pyridine‐ring nitrogen. We observed side reactions leading to preliminary auxiliary loss. We describe a new easy to attach β‐mercapto‐β‐(4‐methoxy‐2‐pyridinyl)‐ethyl (MMPyE) auxiliary, which 1) has increased stability; 2) enables NCL at sterically encumbered junctions (e. g., Leu‐Val); and 3) allows removal under mildly basic (pH 8.5) conditions was introduced. The synthesis of a 120 aa long peptide containing eight MUC5AC tandem repeats via ligation of two 60mers demonstrates the usefulness. Making use of hitherto unexplored NCL to tyrosine, the MMPyE auxiliary provided access to a head‐to‐tail‐cyclized 21‐mer peptide and a His_6_‐tagged hexaphosphorylated peptide comprising 6 heptapeptide repeats of the RNA polymerase II C‐terminal domain.

## Graphical abstract

**A properly positioned pyridine ring** can speed up auxiliary‐mediated native chemical ligation. A methoxy substituent increases auxiliary stability and facilitates rapid syntheses of long and multiphosphorylated multirepeat proteins and of head‐to‐tail cyclized peptides.

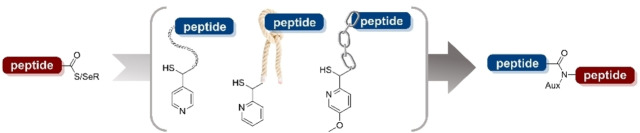

## Introduction

The native chemical ligation (NCL) reaction is a cornerstone of protein total synthesis.^[1]^ The success of the method is due not least to the versatility of the reaction principle. Related approaches such as the ligation‐desulfurization method or the auxiliary‐mediated NCL have considerably extended the scope of the NCL reaction, which in its original form was limited to peptide fragments offering a *N*‐terminal cysteine for reactions with peptide thioesters. The ligation‐desulfurization involves thiolated amino acids, which mimic the 1,2‐aminothiol structure of cysteine. After ligation, the thiol group is removed by a radical reaction.^[2]^ This procedure enables the synthesis of proteins that lack cysteine. However, the synthesis of the thiolated amino acid building blocks is often arduous and low yielding. Furthermore, the method is not applicable for ligations at glycine, and ligations to tyrosine have not yet been reported.

**Figure 1:**
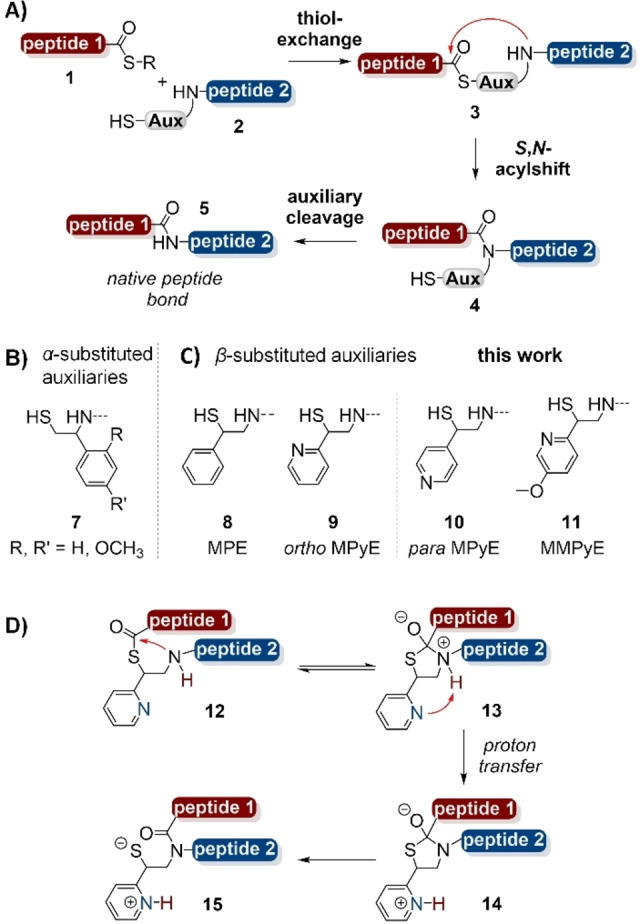
Mechanism of A) auxiliary‐mediated peptide ligation. Structure of B) reported *α*‐substituted mercaptoethyl auxiliaries and C) *β*‐substituted mercaptoethyl auxiliaries. D) Proposed mechanism for MPyE catalyzed *S*,*N*‐acyl shift.

Auxiliary‐mediated NCL is a potentially more convenient approach (Figure 1A). The idea is to create the aminothiol structure by appending a universal, generically applicable auxiliary group to the peptide *N*‐terminus.^[3]^ The auxiliary also serves as an amide protecting group, which eventually is cleaved. Auxiliary‐mediated ligation has the potential to simplify protein(retro)synthesis provided that ligations proceed in useful rates at a wide range of junctions. However, it proved difficult to achieve this goal because *N*‐terminal alkylation reduces the reactivity of the amino group. Exemplary for this problem are the early *N*‐benzyl type auxiliaries (e. g. **7**, Figure 1B).^[4]^ While the thiol exchange step (**1**+**2**→**3**, Figure 1A) can proceed with little hindrance, the *S*,*N*‐acyl transfer step (**3**→**4**) will become challenging if not impossible at amino acids with larger steric demand than glycine. A solution to the problem became apparent when we, while working on alternative auxiliary scaffolds, became aware of a radical fragmentation reaction (see below) that allows removal of non‐benzyl auxiliaries.^[5]^ The 2‐mercapto‐2‐phenylethyl (MPE) auxiliary **8** (Figure 1C) enabled NCL reactions at non‐glycine junctions. However, ligations at *β*‐branched amino acids such as Val remained difficult. Recently, we were able to solve the reactivity issue with a change to the aryl component. The 2‐mercapto‐2‐(pyridin‐2‐yl)ethyl (MPyE) scaffold **9** is the first auxiliary enabling ligations at sterically demanding junctions such as Leu‐Arg, Leu‐Val or Pro‐Ala.^[6]^ The nitrogen of the pyridine ring most likely promotes *S*,*N*‐acyl transfer by base catalysis (Figure 1D).

Herein, we provide experimental data which highlight the important role of the ring nitrogen in the MPyE auxiliary. The work led to the discovery of a side reaction, i. e. premature auxiliary cleavage from non‐ligated peptides. This observation prompted us to improve the auxiliary design. We introduce a methoxy‐ring substituted pyridine‐ethyl ligation template (**11**, Figure 1C). The new auxiliary provides high stability during storage and challenging native chemical ligation reactions at elevated temperature. Applications in the synthesis of a backbone‐cyclized peptide, a 120 aa long coiled coil peptide‐modified mucin peptide and hexaphosphorylated peptides comprising 6 heptapeptide repeats of the C‐terminal domain of the large RNA polymerase II subunit demonstrate the usefulness of the new auxiliary.

## Results and Discussion

The recently introduced MPyE auxiliary enables NCL reactions at ligation junctions inaccessible to previous ligation auxiliaries. For example, we established Ala‐Val, Leu‐Val, Pro‐Ala and Pro‐Arg bonds.^[6]^ We attributed the high reactivity of the MPyE auxiliary to the pyridine nitrogen. Quantum chemical calculations suggested that the nitrogen facilitates *S*,*N*‐acyl transfer by abstracting a proton from the ammonium ion of the tetravalent intermediate **13** (Figure 1D). This proton abstraction, which normally would occur in an intermolecular reaction, is a requirement for formation of the amide bond in product **15**. To probe the role of the ring nitrogen experimentally, we prepared the para isomer of the MPyE auxiliary (Figure 1C, **10**). While shifting the nitrogen from the ortho to the para position should have little effect on its basicity, the intramolecular proton abstraction (visualized in **13**) should be impossible. For the para isomer of the MPyE auxiliary, we therefore expected a ligation speed comparable to that of the MPE auxiliary **8**.

**Figure 2:**
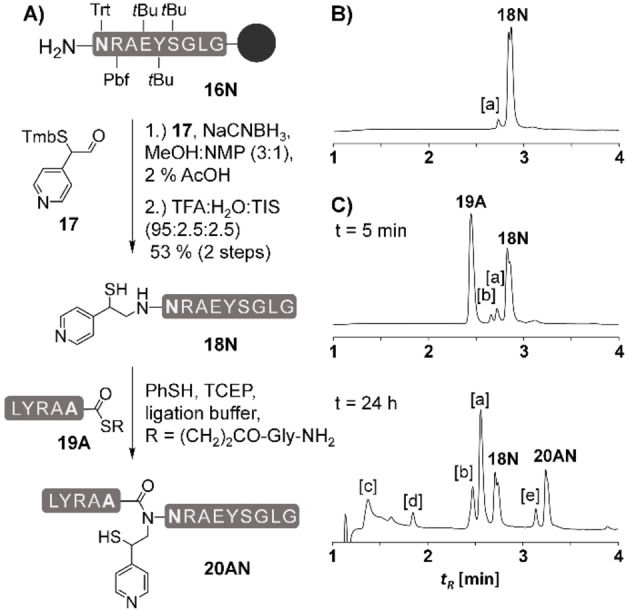
A) Introduction of the para‐MPyE auxiliary and NCL of para‐MPyE‐peptide **18N** with peptide thioester **19A**. Conditions for reductive alkylation: 15 equiv NaCNBH_3_, 15 equiv aldehyde **17**, MeOH/NMP/AcOH (6 : 4 : 0.2 v/v/v), 18 h. Conditions for NCL: 5 mM peptides, 20 mM TCEP, 200 mM Na_2_HPO_4_, 6 M Gdn‐HCl, 3 vol.% PhSH, rt, pH 7.5. Representative UPLC analysis of B) **18N** after HPLC purification and of C) ligation at t=5 min and after t=24 h and treatment with hydrazine (2.5 %). [a] **18N** with cleaved auxiliary. [b] Desulfurization product from **18N**. [c] Peptide hydrazide of **19A**. [d] Hydrolyzed **19A**. [e] Desulfurized **20AN**.

The aldehyde precursor **17** (Figure 2A) required for introduction via reductive alkylation was prepared according to a synthesis route established for the synthesis of the MPyE precursor (Scheme S1). Attachment of the auxiliary to the terminal Asn amino group of a peptide was challenging. At first glance, reductive alkylation seemed inefficient. However, after multiple attempts we found out that isolation of auxiliary‐modified peptide was difficult due to a rapid cleavage of the *para*‐MPyE group. Perhaps surprisingly, this cleavage occurred even during storage as a dry lyophilizate at −20 °C (Figure S17D). Under these conditions, the original ortho isomer of the MPyE auxiliary remained stable. Nevertheless, with quick and careful handling we succeeded in the isolation of product (53 %) and subsequently analyzed reactions of the *para*‐MPyE peptide with the peptide thioester **19A**. The attempted Ala‐Asn ligation was slow (Figure 2C). Only 19 % of starting material was converted to the corresponding ligation product within 24 h. Under similar conditions the *ortho*‐MPyE auxiliary furnished 83 % ligation product (Figure S20E). The decrease of ligation rates upon para positioning illustrates the role of the ring nitrogen, which must be in the ortho position to allow acceleration of the *S*,*N*‐acyl transfer by intramolecular base catalysis.

**Figure 3:**
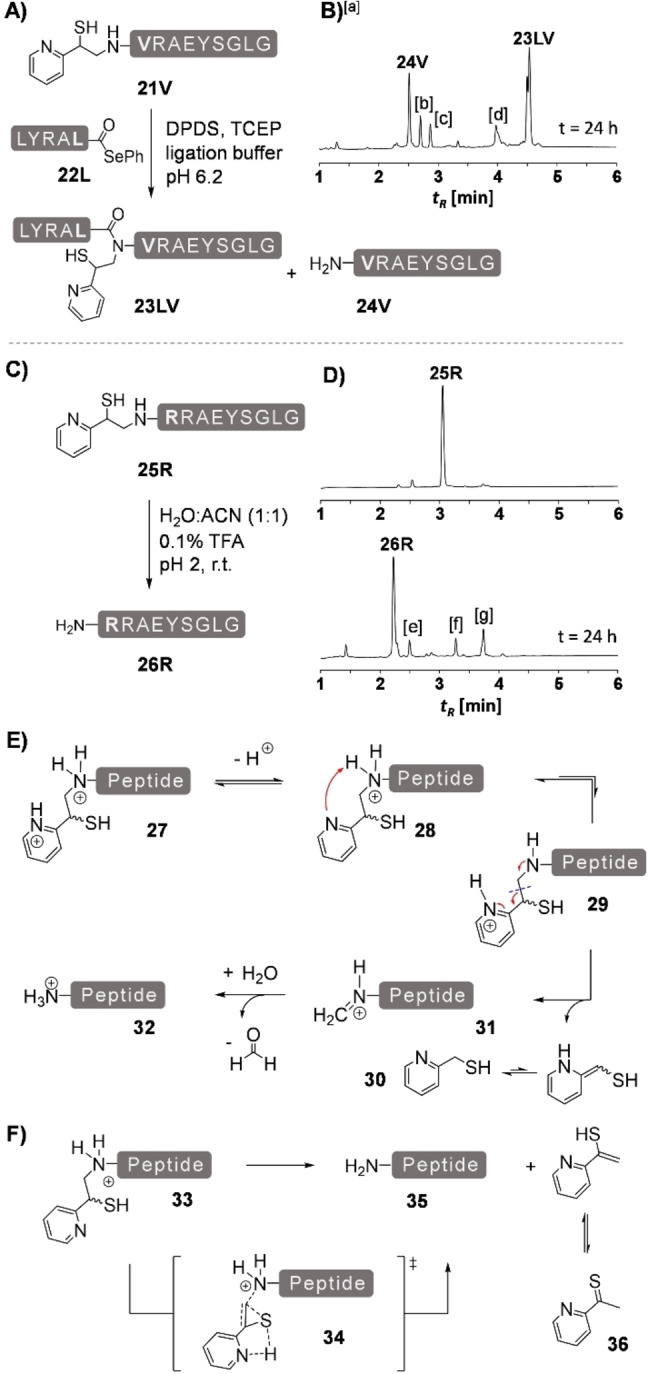
A) Stability of the MPyE auxiliary during selenoester ligation at the Leu‐Val junction. B) UPLC trace after 24 h of ligation and C) incubation of **25R** in acidic medium (pH 2) shows premature auxiliary cleavage. Proposed mechanism for cleavage of the MPyE auxiliary under mild acidic conditions via E) a retro‐Mannich reaction or F) anchimerically assisted Hoffmann Elimination. [a] UPLC sample was treated with hydrazine (2.5 %). [b] Desulfurized **24V**. [c] Hydrolyzed **22L**. [d] Desulfurized **23LV**. [e] Desulfurized **25R**. [f] [**26R**+66 Da]. [g] [**26R**+135 Da].

The rapid cleavage of the *para*‐MPyE group from peptide **18N** prompted us to also critically analyze the stability of the *ortho*‐MPyE auxiliary. In a published example^[6]^, we reported a selenoester‐mediated ligation establishing the difficult Leu‐Val junction in test peptide **23LV** (Figure 3A), which was impossible to achieve with previous auxiliaries. The reaction furnished 63 % ligation product, but the yield could have been even higher without formation of by‐product **24V**, which is the C‐terminal peptide fragment **21V** lacking the auxiliary (see also Figure 3B). This by‐product was also observed in an Ala‐Val (Figure S20A) and a Pro‐Arg (Figure S20B) selenoester ligation. Selenoester ligations are performed at slightly acidic pH 6.2. Usually, peptides are eluted via an acidic solvent mixture on the HPLC and converted to the dry TFA salt via lyophilization. We therefore analyzed the stability of MPyE‐peptides before ligation under storage conditions. While stable in dry form at −20 °C, the MPyE auxiliary was quantitatively cleaved within 24 h when peptides such as **25R** were kept as TFA salt in aqueous solution (Figure 3 C, D). The cleavage of the MPyE auxiliary possibly proceeds through a retro‐Mannich reaction (Figure 3E). Protonation of the N‐terminal amino group could trigger a fragmentation reaction yielding 2‐picolinethiol (**30**) and iminium ion **31**. The latter would undergo rapid hydrolysis. Alternatively, the ethylpyridine structures may undergo a Hoffman elimination facilitated by anchimeric assistance from a deprotonated thiol group, which would be considerably acidified by pyridine protonation (Figure 3F). Both mechanisms suggest a role of the basicity of the ring nitrogen. At strongly acidic conditions, the pyridine ring, the N‐terminal amine and the thiol will be protonated. Neither the retro‐Mannich reaction nor the anchimerically assisted Hoffman elimination could occur explaining why the auxiliary withstands the TFA cleavage conditions. On the other hand, without protonation cleavage cannot occur and MPyE peptides are stable when native chemical ligation is performed at pH>7 (Figure S20) but can suffer from cleavage at prolonged reaction times under pH<7. The proposed retro‐Mannich mechanism also explains the rapid loss of the para MPyE auxiliary. The cleavage is probably particularly fast here because the proton on the pyridine nitrogen cannot be shifted to the N‐terminal amine via an intramolecular exchange as in the ortho‐isomer.

The considerations on the stability and reactivity of the MPyE auxiliary indicate an important role of the ring nitrogen. We examined methoxy substitution in meta position to the nitrogen. Methoxy substitution decreases the basicity of pyridine nitrogen (pK_A_ (3‐MeO‐pyridine)=4.88; pK_A_ (pyridine)=5.17)^[7]^, which should improve the stability of the auxiliary under mildly acidic conditions. In addition, this substitution provides an additional means to probe intramolecular base catalysis in NCL reactions.

**Figure 4:**
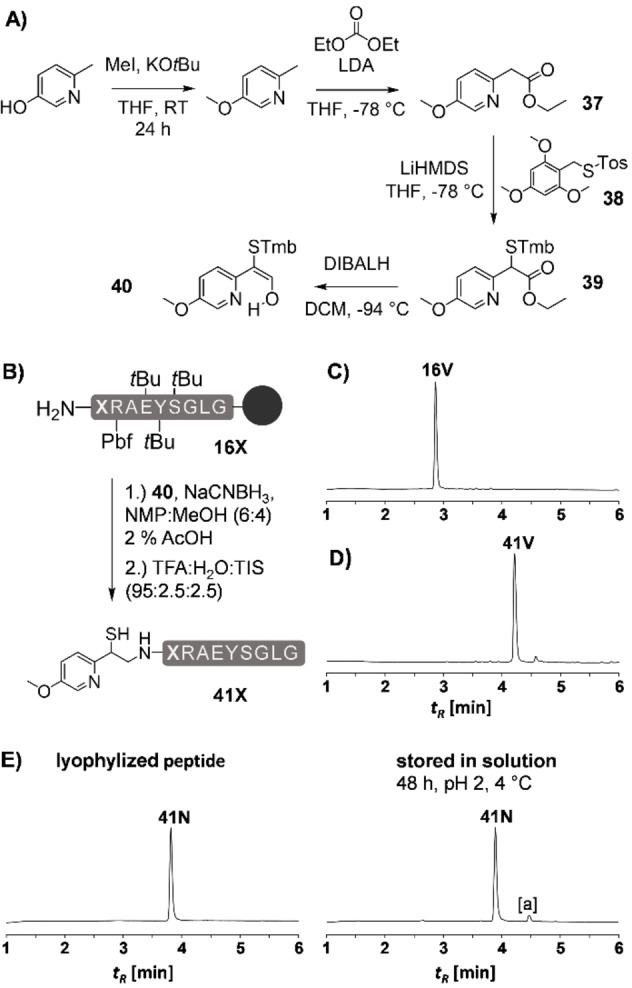
A) Synthesis of auxiliary precursor **40** and B) introduction of this compound to resin‐bound peptides **16X** via reductive alkylation. UPLC analysis of C) crude peptide **16 V** (X=Val) before and D) after 18 h of reductive alkylation with **40**. E) UPLC trace of purified **41N** after lyophilization and after incubation at pH 2 for 48 h. [a] Disulfide of **41N**.

For the introduction of the MMPyE auxiliary, a suitable precursor was synthesized in 4 steps (Figure 4A). In the first step, 5‐hydroxy‐2‐methylpyridine was O‐methylated with methyl iodide.^[8]^ The resulting compound was then deprotonated at the *α*‐methyl carbon with lithium diisopropylamide and subjected to a nucleophilic acylation with diethyl carbonate to yield methyl (5‐methoxy‐2‐pyridinyl)acetate (**37**). Treatment with lithium bis(trimethylsilyl)amide afforded an ester enolate which was converted to the *α*‐mercapto ester **39** in an electrophilic thiolation. Reduction of **39** with diisobutylaluminium hydride (DIBALH) furnished **40** as a suitable precursor for auxiliary introduction by reductive alkylation. Compound **40** is present in its enole form and shelf‐stable.

We next attached the MMPyE auxiliary to the unprotected N‐terminus of asparagine and valine test peptides (Figure 4B). After reductive alkylation with sodium cyanoborohydride and **40** (15 equiv each) in presence of 2 % acetic acid, the peptides were treated with a mixture of TFA/H_2_O/TIS (95/2.5/2.5 v/v/v) for final deprotection and cleavage of the peptides from resin. The crude peptides were obtained in high purity, indicative of efficient reductive alkylation (Figure 4D). The methoxy substitution in the MMPyE auxiliary resulted in a marked increase of stability. No cleavage occurred when **41N** was incubated for 48 h in aqueous solution at pH 2 (Figure 4E). The analysis of valine peptide **41V** confirmed the high acid stability. More than 98 % of the MMPyE‐peptide **41V** remained unharmed after a 24 h incubation at pH 2 (Figure S22).

**Figure 5:**
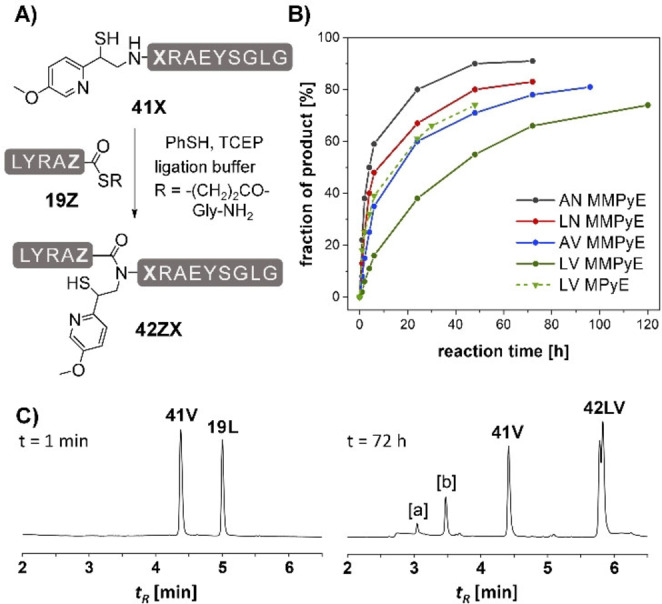
A) MMPyE‐induced ligation between peptide thioesters **19Z** and peptides **41X** (letters Z and X represent amino acids specified in the figure legend). B) Time course of ligation at four different junctions. Representative UPLC analysis for the ligation of peptides **41V** and **19L** C) at t=1 min and t=72 h and treatment with hydrazine (2.5 %). Conditions: 5 mM peptides, 20 mM TCEP, 200 mM Na_2_HPO_4_, 6 M Gdn‐HCl, 3 vol.% PhSH, rt, pH 7.5. [a] **41V** with cleaved auxiliary. [b] Hydrolyzed **19L**.

Next, we investigated how the methoxy substituent affects the NCL reactions at Ala‐Asn, Leu‐Asn, Ala‐Val and Leu‐Val junctions (Figure 5, Figure S25–S28). For the reactions of the MMPyE peptides **41X** with the peptide thioesters **19Z** the compounds were dissolved in a mixture prepared by adding 3 vol.% thiophenol to a phosphate buffer containing 6 M guanidinium hydrochloride with an apparent pH 7.5. The ligations proceeded smoothly. Compared to reactions at the non‐substituted MPyE peptide we observed the expected decrease in the reaction rate (Figure 5B). After adjustment of reaction times, the final yields with both auxiliaries were comparable. The high stability of the MMPyE scaffold is advantageous when ligations are performed at sterically demanding peptide bonds. For example, in an attempt to establish the Leu‐Val junction on the MPyE auxiliary, we observed the premature cleavage of this auxiliary (see Figure S20D). By contrast, the MMPyE auxiliary remained stable even after 72 h reaction time (Figure 5C). An additional advantage offered by the improved stability is that reactions can be carried out at elevated temperature. Performing the Leu‐Val ligation on the MMPyE auxiliary at 50 °C for 24 h afforded 76 % ligation product, compared to 51 % achieved with the MPyE auxiliary at room temperature (Figure S31, S32). Also for the Ala‐Asn ligation, increasing temperature is an option to accelerate ligation at the MMPyE auxiliary to an extent that exceeds rates provided by the non‐substituted MPyE scaffold at ambient temperature (Figure S31A). For the non‐substituted MPyE group, performing reactions at elevated temperature is not a viable option as this could result in increased auxiliary cleavage. The new MMPyE group therefore seems to us to be the more suitable ligation auxiliary.

**Figure 6:**
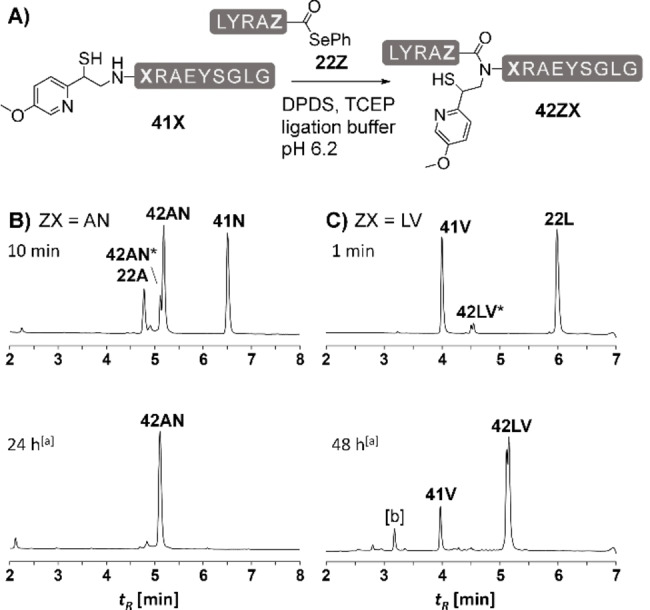
A) MMPyE‐induced ligation between peptide selenoesters **22Z** and auxiliary peptides **41X**. Representative UPLC analysis for B) the ligation of peptides **22A** and **41N** after 10 min and after 24 h with hydrazine (2.5 %) added and C) ligation of peptides **22L** and **41V** after 1 min and after 24 h with hydrazine (2.5 %) added. Conditions: 5 mM peptides, 100 mM TCEP, 200 mM Na_2_HPO_4_, 6 M Gdn‐HCl, 28 mM DPDS, rt, pH 6.2. [a] UPLC sample was treated with 2.5 % hydrazine. [b] Hydrolyzed **22L**. *ligated peptide **42ZX** before *S*,*N*‐acyl shift.

We next explored the MMPyE auxiliary in ligations with selenoesters, which are useful alternatives when conventional NCL reactions via thioesters are too slow (Figure 6A). After 10 min of Ala‐Asn ligation, a large portion of starting materials **22A** and **41N** was already converted to the desired product (Figure 6B). Interestingly, the thioester‐linked intermediate 42AN*, formed by the reaction of the selenoester with the auxiliary thiol, was still detectable via UPLC analysis. This intermediate could not be observed with the MPyE auxiliary (see Figure 3 in [6]), indicating again, that the additional methoxy group moderately lowers the rate of *S*,*N*‐acyl transfer. Nevertheless, complete conversion to ligated Ala‐Asn peptide was observed in less than 24 h. We also tested the MMPyE auxiliary in a Leu‐Val ligation using selenoesters. For the MPyE ligation, this reaction was accompanied by decomposition of the auxiliary peptide (see Figure 3A). MMPyE‐mediated ligation enabled a direct conversion to product **42LV** without formation of the unwanted by‐product (Figure 5C).

**Figure 7:**
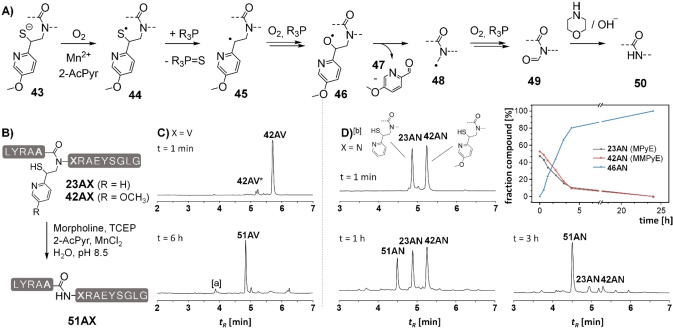
A) Proposed mechanism for auxiliary removal via radical‐induced oxidative fragmentation. B) Removal of the MPyE auxiliary from ligation products **23ZX** and **42ZX**. Representative UPLC analysis of MMPyE cleavage from C) peptide **42AV** C) at t=1 min and after 6 h, or D) a 1 : 1 mixture MMPyE/MPyE at 1 min, 1 h and 3 h. Conditions: 2 M Morpholine, 0.5 M TCEP, 0.5 M 2‐acetylpyridine, 5 mM MnCl_2_, rt, pH 8.5. [a] non‐peptidic material. The boxed graphic shows the fraction of compounds as the reaction progresses. [b] samples for the UPLC analysis were quenched with an aqueous solution of ascorbic acid (0.1 M). 42AV*=thioester formed by *N*,*S* rearrangement in acidic medium.

In a next set of experiments, we explored the detachment of the MMPyE auxiliary from the ligated peptides. Initially, we had envisioned that the methoxy substitution facilitates radical cleavage due to stabilization of the benzyl radical **45** (see Figure 7A, for radical energies see [9]). The methoxy substituent could also accelerate cleavage by phenylogous stabilization of the aldehyde **47** formed upon fragmentation. Applying the previously reported conditions (TCEP, morpholine, MnCl_2_ and 2‐acetylpyridine, pH 8.5), we observed a rapid auxiliary cleavage within 3 to 6 h (Figure 7B, C). For an accurate comparison of cleavage rates, a 1 : 1 mixture of both the MMPyE and the MPyE peptides **23AN** and **42AN**, respectively, was subjected to the auxiliary cleavage conditions. UPLC‐MS analysis (Figure 7D) revealed that, contrary to our expectations, both peptides were converted at similar rates. Perhaps, formation of the thiyl radical **44** is rate limiting under the reaction conditions.

**Figure 8:**
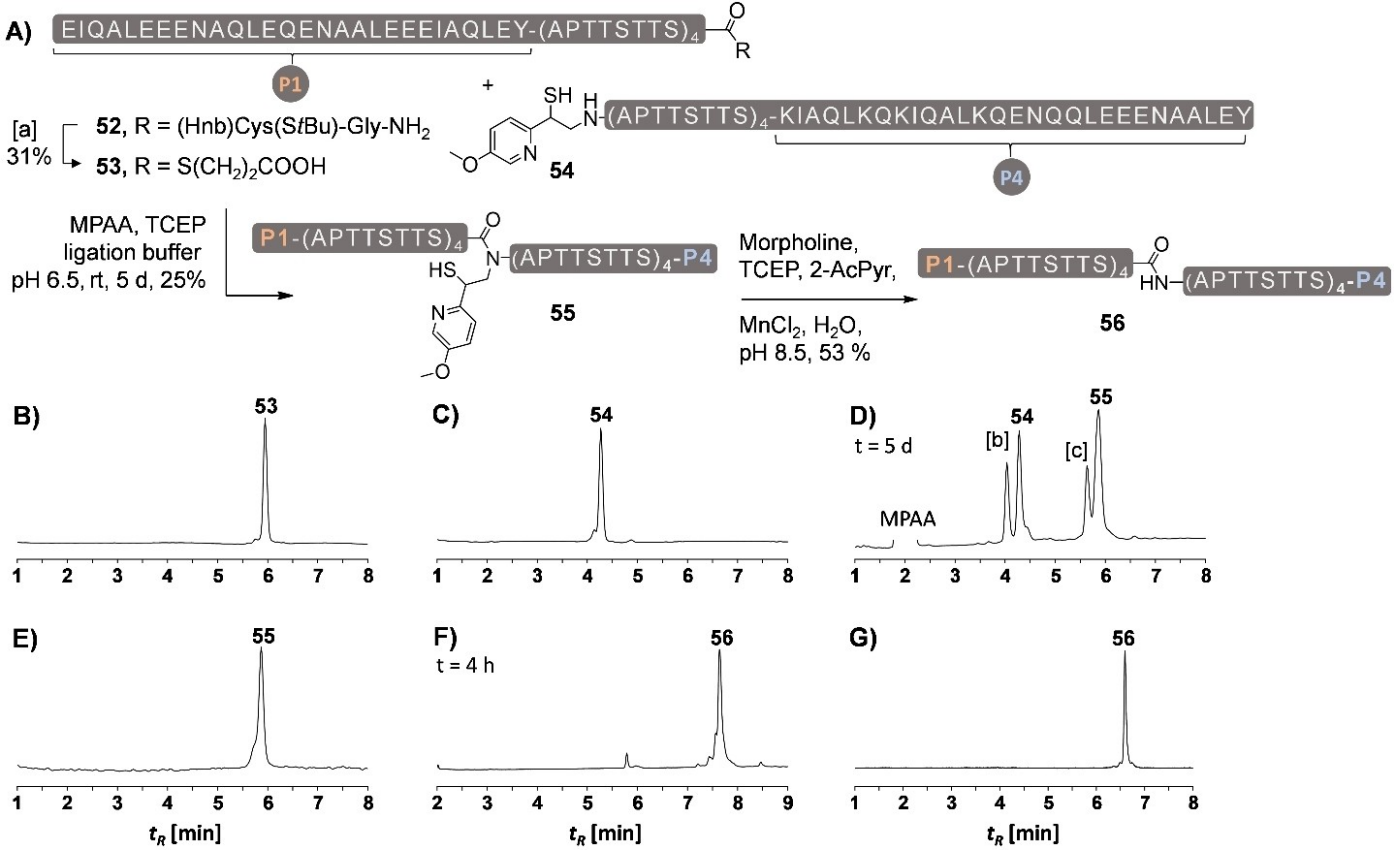
A) Synthesis of a 120 aa MUC5AC peptide P1‐(APTTSTTS)_8_‐P4 (**56**) by ligation of 60 aa thioester **53** (1.5 equiv) and 60 aa MMPyE‐peptide **54** (1.0 equiv) and subsequent auxiliary removal. UPLC trace after HPLC purification of B) thioester **53** and C) auxiliary peptide **54**. Representative UPLC analysis D) after 5 days ligation and treatment with hydrazine (2.5 %), E) of purified **55**, F) after 4 h auxiliary removal from purified ligation product and G) of purified **56**. [a] Conditions: 2 mM Peptide, 10 vol.% MPA, 100 mM TCEP, 200 mM Na_2_HPO_4_, 6 M Gdn‐HCl, 37 °C, pH 6.5, 18 h. [b] **54** with removed auxiliary. [c] peptide hydrazide of **53**.

We explored the usefulness of the MMPyE auxiliary and embarked on the synthesis of mucin repeat domains (Figure 8). Our long‐term goal is to develop a method for stitching of MUC5AC tandem repeats through coiled coil motifs. The MUC5AC octapeptide tandem repeat domain lacks cysteine.^[10]^ We selected the Ser‐Ala junction for connecting two 60 aa long peptides **53** and **54** (Figure 8A). The peptides were comprised of four repeats and were equipped at either the N‐ or the C‐terminus with 28 aa long orthogonal coiled coil peptides P1 and P4. *Jerala* recently reported a set of orthogonal coiled coils comprised of P1⋅P2, P3⋅P4 and P5⋅P6 pairs, among others.^[11]^ The goal is to obtain final product **56** that can be dimerized upon coiled coil formation with a P3‐peptide‐P5 fusion and use the ‘sticky’ coiled coil ends for further modifications. For the synthesis of the peptide thioester **53**, we employed the crypto thioester methodology from *Aucagne* (Figure S38).^[12]^ The 62 aa long crypto thioester peptide **52** was prepared by microwave‐assisted solid phase peptide synthesis (MW‐SPPS) and used in crude form in the reaction with mercaptopropionate (MPA). The conversion to the MPA ester **53** proceeded smoothly (Figure 8B). The C‐terminal segment **54** was also accessed by linear MW‐SPPS and reductive alkylation with **40** in 57 % overall yield (Figure 8C). In the event of ligation, we added MPAA as thiol additive. The handling of coiled coil‐modified peptides is difficult. While formation of orthogonal coiled coil duplexes is selective at micromolar concentration, the coil peptides tend to aggregate at higher concentration. Under the chosen conditions the reaction on the MMPyE scaffold required five days to form the ligation product as the main component of the reaction mixture (Figure 8D). The ligation was performed at pH 6.5 in order to minimize thioester hydrolysis. Under these conditions, the ligation was accompanied by small degree of premature auxiliary cleavage. Such cleavage reactions were not observed in test reactions with model peptides (Figure S21–S24), which is indicative of a sequence‐specific reactivity. We wish to note that previous attempts to perform such ligations with the unsubstituted MPyE auxiliary were unsuccessful. The HPLC purified ligation product **55** (Figure 8E) was submitted to the auxiliary cleavage conditions. After only 4 h detachment was complete (Figure 8F) affording the 120 aa long target peptide **56** in 53 % yield.

**Figure 9:**
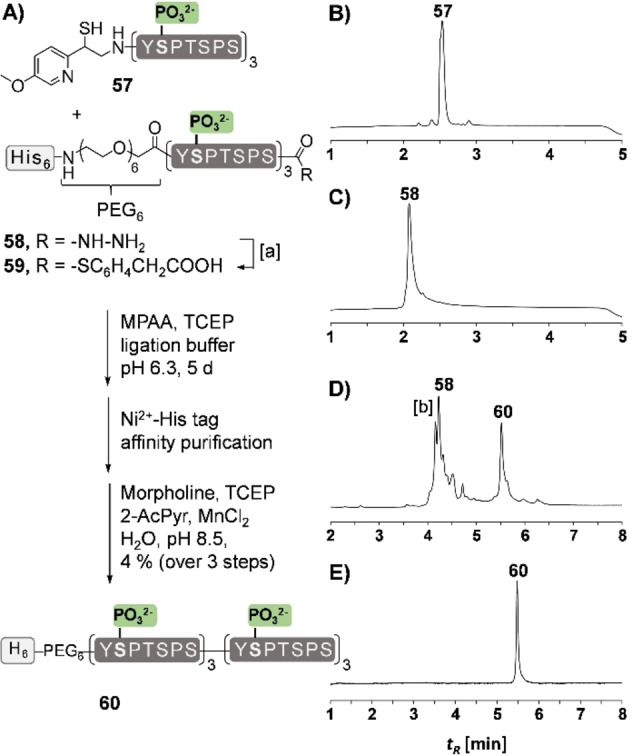
A) Synthesis of His_6_‐tagged hexaphosphorylated peptide comprising 6 heptapeptide repeats of the C‐terminal domain of the large RNA‐Pol‐II subunit. UPLC analysis of B) purified MMPyE‐peptide **57**, C) purified peptide hydrazide **58**, D) crude material obtained after conversion of **58** to the peptide thioester **59**, addition of MMPyE‐peptide **57** and reaction for 5 days, quenching with hydrazine, affinity purification and auxiliary removal for 24 h. E) UPLC analysis of product **60** obtained after HPLC purification. [a] Conditions: Acetylacetone (1.5 equiv), 200 mM MPAA, 6 M Gdn• HCl, pH 3. [b] peptide acid formed upon hydrolysis of peptide thioester **59**.

**Figure 10:**
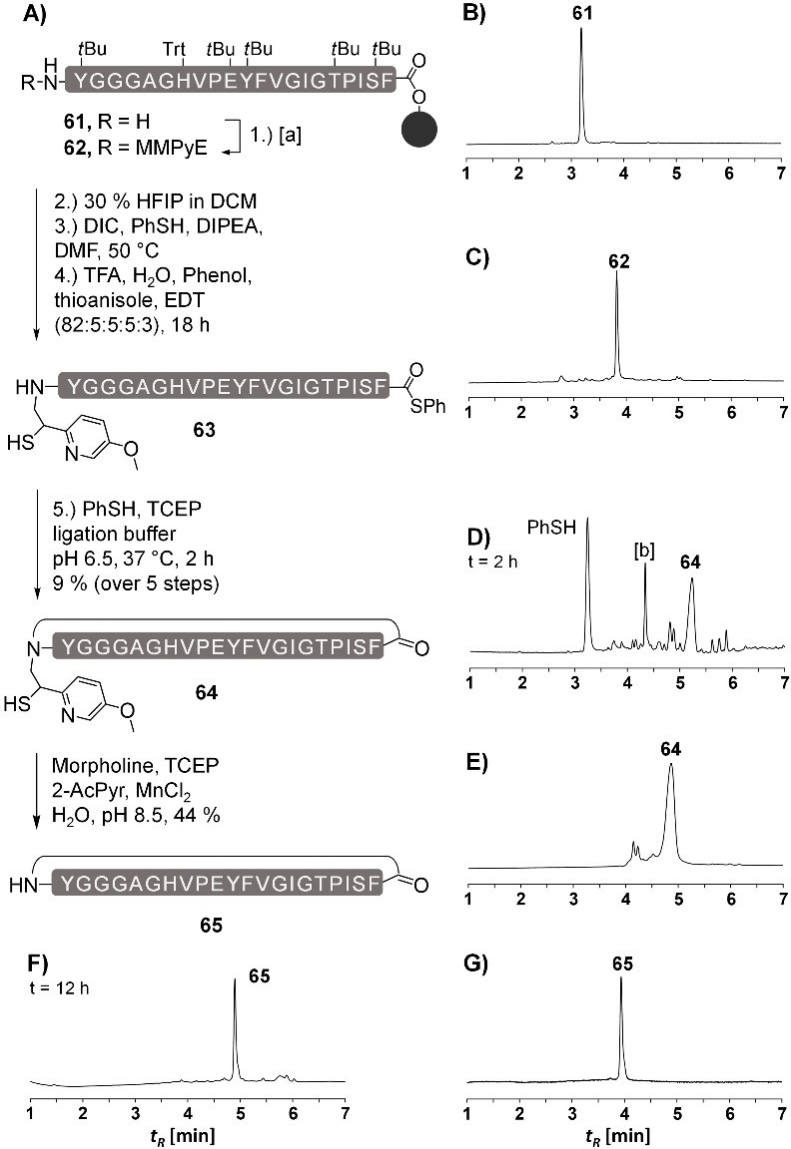
A) Synthesis of a cyclic 21 aa microcin **65** via cyclization of MMPyE‐thioester peptide **63** and subsequent auxiliary removal. UPLC analysis of aliquots withdrawn B) from crude peptide **61** after SPPS and C) from peptide **62** following auxiliary introduction and treatment with NaHCO_3_ (to a pH of 7) and TCEP (50 mM), D) after cleavage from resin with HFIP (30 vol.% in DCM), thioester formation, TFA deprotection and cyclization in NCL buffer for 2 h and E) of purified peptide **64**. UPLC trace F) after 12 h auxiliary removal from purified ligation product and G) of purified **65**. Chromatogram F) includes a 1 min wash to accommodate the high load of non‐peptide material. [a] Conditions: **40** (7 equiv), NaCNBH_3_ (16 equiv), 2 % AcOH, MeOH/NMP (6 : 4 v/v). [b] hydrolyzed **63**.

Due to our interest in post‐translationally modified multirepeat proteins, we tested the auxiliary in the rapid synthesis of a hexaphosphorylated peptide comprising 6 heptapeptide repeat units from the C‐terminal domain of the large RNA polymerase II subunit.^[13]^ The synthesis of multiphosphorylated peptides is a challenge.^[14]^ We therefore selected a synthesis route, in which two triple‐phosphorylated peptide segments containing three YSPTSPS repeats are joined by a Ser‐Tyr ligation (Figure 9A). To the best of our knowledge, ligations at tyrosine have not been reported previously. The MMPyE auxiliary was introduced by reductive alkylation after solid‐phase assembly of the triple‐phosphorylated 21‐mer. HPLC‐purification afforded the C‐terminal segment **57** in 35 % yield. The N‐terminal segment **58** was equipped with a hexahistidine tag to allow rapid purification (and immobilization onto microtiter plates for binding experiments). For an approach to a peptide thioester, we first synthesized the peptide hydrazide by microwave‐assisted solid‐phase synthesis. *Dawson's* acetoacetone method was applied to establish the peptide thioester **59** without detriment to the three phospho sites (Figure S45).^[15]^ The mixture after acetoacetone treatment was used without purification in the ligation reaction. HPLC analyses of aliquots withdrawn from the reaction mixture indicated a slow ligation and a comparably high degree of thioester hydrolysis (Figure S45). Of note, ligation of non‐phosphorylated peptides (Figure S44) proceeded smoother, while NCL between two hexaphosphorylated peptides to form a peptide containing 12 pSer residues was extremely difficult (data not shown). After 5 days the reaction between **59** and **57** was discontinued by treatment with hydrazine. Though our aim was to develop a method providing rapid access of product without isolation of intermediates, we also analyzed the ligation mixture (Figure S45B). We observed an unexpected by‐product resulting from cleavage of the N‐terminal tyrosine from peptide **57**. This side reaction was less pronounced in ligations with non‐phosphorylated peptides (Figure S44B) and did not occur at all with a test peptide (Figure S24). At current, we cannot explain this unusual side reaction. Other ligations at tyrosine (see Figure 10, S47, S48) proceeded without cleavage and we therefore attribute the surprising loss of N‐terminal tyrosine to a sequence‐dependent reaction. In spite of these challenges, the affinity purification with Ni^2+^‐loaded agarose beads provided a convenient means of enriching ligation product. Material eluted from the Ni^2+^ beads was incubated with the auxiliary removal cocktail for 24 h. UPLC‐MS analysis (Figure 9D) showed the desired product **60** and, as expected for His‐tag purification, the remainders of the N‐terminal segment; peptide acid from hydrolysis of the peptide thioester **59** and peptide hydrazide formed during hydrazine treatment. Isolation of the product by HPLC was facile and afforded the hexaphosphorylated His_6_‐(Y*p*SPTPS)_6_ peptide **60** in pure form.

In a further exploration of application scenarios, we studied a cyclopeptide. The chemical synthesis of cyclopeptides is an active area in medicinal peptide chemistry and NCL is powerful method to achieve cyclization of unprotected linear peptide precursors.^[16]^ In pioneering work, the ligation‐desulfurization approach was used to synthesize a head‐to‐tail cyclized 21‐mer by establishing a Gly‐Ala bond.^[2a]^ The peptide sequence was identical to the primary structure of microcin J25 (MccJ25), a potent inhibitor of bacterial transcription. Although the MccJ25 structure was later revised to include an 8‐mer side chain‐to‐tail cycle,^[17]^ we decided to demonstrate the broad applicability of the new MMPyE auxiliary in a cyclization reaction that forms a Phe‐Tyr bond (Figure 10A). The Fmoc‐based solid phase synthesis of a linear precursor of cyclization was commenced from Phe‐loaded resin. First attempts with the crypto thioester method revealed rather low purities of crude 21‐mer obtained after acid cleavage. We changed to synthesis on Phe‐loaded chlorotrityl resin and the use of pseudoproline building blocks. Indeed, test cleavage prior to introduction of the auxiliary showed high quality crude material (Figure 10B). The N‐terminal Tyr residue was submitted to the reductive alkylation. Analysis of crude material showed trifluoroacetylation (Figure S46B). The TFA adduct was cleavable by treatment with mild base (Figure S46C). We decided to perform mild acid cleavage,^[18]^ thioesterification with the protected peptide in solution, TFA cleavage and NCL without purification of intermediates. Of note, NCL occurred already during lyophilization of the deprotected peptide thioester and afforded the cyclopeptide in a remarkable purity (Figure S48B). Auxiliary removal proceeded cleanly (Figure 10F) and provided the target cyclopeptide in 44 % yield after HPLC purification (Figure 10G).

Previously we have shown that cysteine residues should be protected during auxiliary removal.^[5b]^ A recurring phenomenon is that peaks prior to auxiliary cleavage are broad (compare Figure 8E with 8G, 10E with 10G). This may be due to cis‐trans isomerism at the N‐alkylated peptide bond and epimeric forms at the auxiliary's chiral center. These isomers cannot exist in absence of the auxiliary and, as a result, peaks sharpen after auxiliary cleavage.

## Conclusion

Native chemical ligation reactions on *β*‐mercapto‐*β*‐(2‐pyridinyl)‐ethyl auxiliaries proceed in high rate and enable joining of peptide fragments at junctions, such as the Leu‐Val bond inaccessible to previous ligation auxiliaries. In this work, we probed the role of the *ortho*‐positioned ring nitrogen. A comparison with the *para* isomer (*β*‐mercapto‐*β*‐(4‐pyridinyl)‐ethyl) scaffold revealed the superior reactivity of the ortho pyridine derivatives. We attribute the high reactivity to the *ortho* ring nitrogen abstracting a proton from an ammonium ion formed when the amino group intramolecularly attacks the thioester bond. Without deprotonation the *S*,*N*‐acyl shift cannot proceed and the reaction stops at the thioester intermediate stage. Our investigations also uncovered a peculiar lability of peptides modified with the *β*‐mercapto‐*β*‐(4‐pyridinyl)‐ethyl) group at the N‐terminus. While the para‐pyridine auxiliary‐peptide bond was stable during TFA cleavage, rapid cleavage occurred at moderately acidic pH 2. This reaction only occurs prior to ligation indicating a role of the amino terminus’ lone pair. Though at a slower rate, premature auxiliary loss was also observed with the ortho‐system (*β*‐mercapto‐*β*‐(2‐pyridinyl)‐ethyl). Importantly, our studies show that the auxiliary gains stability when a methoxy substituent is added at the 4‐position of the ortho‐pyridine ring (i. e. meta to the ring nitrogen).

The new MMPyE (*β*‐mercapto‐*β*‐(4‐methoxy‐2‐pyridinyl)‐ethyl) auxiliary is conveniently attached to the peptide N‐terminus in the last step of solid‐phase synthesis by reductive alkylation. Despite slightly lowered ligation rates, the MMPyE auxiliary is better suited for NCL at challenging junctions than the MPyE auxiliary becausethe increased stability is a significant advantage when reactions such as the selenoester‐NCLs are performed at slightly acidic conditions. Reactions proceed cleanly also when long reaction times are needed in ligations of large peptides. Furthermore, the increased stability enables ligations at elevated temperature. The usefulness of the MMPyE auxiliary was demonstrated in three applications. The synthesis of a 120 aa long peptide containing 8 MUC5AC tandem repeats and two “sticky” coiled coil peptides involved the smooth ligation of two 60mers via a Ser‐Ala junction. Using a hitherto unexplored ligation site in NCL chemistry and demonstrating the potential for the preparation of post‐translationally modified peptides, we applied the MMPyE auxiliary in a Ser‐Tyr ligation for the synthesis of a His_6_‐tagged hexaphosphorylated peptide spanning 6 tandem repeats of the RNA polymerase II C‐terminal domain. The rapid synthesis of a Phe‐Tyr‐cyclized 21 aa peptides comprising the primary sequence of microcin J25 illustrates an application of the auxiliary in head‐to‐tail cyclization reactions.

With the MMPyE auxiliary we laid out a blueprint for the design of new auxiliaries, which 1) are stable over a wide pH range, 2) enable native chemical ligations at sterically encumbered junctions, 3) allow removal under mildly basic (pH 8.5) conditions and 4) provide options for attaching further handles by means of 4‐alkoxy substituents. The last option offers prospects for the introduction of functional units such as oligoethylene glycol or oligoarginine units that enhance the solubility of unfolded peptide segments.^[19]^ This could prove particularly valuable in multi segment ligation approaches where multiple auxiliaries would act as solubility‐enhancing amid protecting groups that would be removed in the last step. In this context, it is encouraging that the *β*‐mercapto‐*β*‐2‐pyridineethyl groups provide useful ligation rates at a wide range of ligation junctions.

## Experimental Section

### Synthesis of para MPyE‐Auxiliary Precursor 17

**Ethyl 2‐(pyridin‐4‐yl)‐2‐((2,4,6‐trimethoxybenzyl)thio)acetate (S4)**: Under argon atmosphere, lithium hexamethyldisilazide (4.03 mL, 1.3 M in THF, 5.25 mmol, 1.05 equiv) was added to a stirred solution of ethyl 2‐(pyridin‐4‐yl)acetate (**S3**, 840 mg, 5.0 mmol) in 75 mL anhydrous THF over the course of 25 min at −78 °C. After 30 min of stirring, thiosulfonate **38** (2.05 g, 5.50 mmol, 1.1 equiv, see Suppl. Inf.) in 40 mL anhydrous THF was added at −78 °C over 100 min. The reaction mixture was allowed to gradually warm to room temperature over the course of 30 min and a saturated solution of NH_4_Cl was added. The resultant mixture was extracted with EtOAc (3x) and the combined organic layers were dried over MgSO_4_, filtered, and concentrated under reduced pressure. The residue was purified by flash column chromatography on silica gel (EtOAc) to afford desired product (1.55 g, 4.10 mmol) in 82 % yield as a white solid. ^1^H NMR (CD_3_CN, 500 MHz): δ [ppm]=8.52 (d, *J*=4.4 Hz, 2H), 7.38 (d, *J*=4.4 Hz, 2H), 6.16 (s, 2H), 4.66 (s, 1H), 4.12 (q, J=7.2 Hz, 2H), 3.78 (s, *3*H), 3.75 (s, 6H), 3.75 ‐ 3.72 (m, 2H), 1.19 (t, *J*=7.2, 3H). ^13^C NMR (CD_3_CN, 126 MHz): δ [ppm]=170.94, 161.94, 159.85, 150.77, 147.17, 124.38, 107.02, 91.64, 62.74, 56.44, 56.06, 52.28, 25.19, 14.32.

**2‐(Pyridin‐4‐yl)‐2‐((2,4,6‐trimethoxybenzyl)thio)acetaldehyde (17)**: Under argon atmosphere ester **S4** (444 mg, 1.0 mmol) was dissolved in 5 mL anhydrous DCM and cooled to ‐94 °C. Diisobutylaluminium hydride (3 mL, 1.0 M in toluene, 3 mmol, 3.0 equiv) was added carefully over the vessel wall over the course of 1 h. The reaction mixture was stirred for an additional 15 min at −94 °C. Subsequently, a mixture of DCM/MeOH (1 mL, 3 : 1 v/v) was added via the vessel wall over the course of 30 min at −94 °C. The reaction mixture was stirred at room temperature for 10 min and a saturated solution of potassium sodium tartrate and DCM (20 ml each) was added. The resulting mixture was stirred at room temperature for 1 h and then extracted with DCM (3x). The combined organic layers were dried over MgSO_4_, filtered and concentrated. The residue was purified by flash column chromatography on silica gel (EtOAc) to afford the aldehyde **17** (247 mg) as yellow oil. The material was used for reductive alkylation despite impurities from remaining solvents and partial decomposition. ^1^H NMR (CD_3_CN, 500 MHz): δ [ppm]=9.52 (d, J=2.5 Hz, 1H), 8.52 (d, J=6.2 Hz, 2H), 7.27 (d, J=6.2 Hz, 2H), 6.10 (s, 2H), 4.62 (d, J=2.5 Hz, 1H), 3.78 (s, 3H), 3.77 (s, 6H), 3.75 (s, 2H).

### Synthesis of MMPyE‐Auxiliary Precursor 40

**Ethyl 2‐(5‐methoxypyridin‐2‐yl)acetate (37)**: *n*BuLi (18.2 mL, 2.5 M solution in hexane, 45.5 mmol, 3.5 equiv) was added dropwise to a stirred solution of diisopropylamine (6.6 mL, 46.8 mol, 3.6 equiv) in 20 ml anhydrous THF at −78 °C under argon. The resulting solution was slowly warmed to room temperature and was added dropwise over 1 h to a stirred solution of 5‐methoxy‐2‐methylpyridine (1.6 g, 26.0 mmol) and anhydrous diethylcarbonate (3.2 ml, 26.0 mmol, 2.0 equiv) in 26 ml anhydrous THF at −78 °C. After 2 h of reaction, the solution was slowly warmed to room temperature and a saturated solution of NH_4_Cl was added. The resultant mixture was extracted with EtOAc (3x), and the combined organic layers were dried over MgSO_4_, filtered, and concentrated under reduced pressure. The residue was purified by flash column chromatography on silica gel (cyclohexane/EtOAc=6 : 4 v/v) to afford **37** (807 mg, 4.2 mmol, 32 %) as a light‐yellow oil. ^1^H NMR (CDCl_3_, 300 MHz): δ [ppm]=8.25 (dd, J=2.8, 0.9 Hz, 1H), 7.28–7.20 (m, 2H), 4.18 (q, J=7.1 Hz, 2H), 3.86 (s, 3H), 3.82 (s, 2H), 1.26 (t, J=7.1 Hz, 3H). ^13^C NMR (CDCl_3_, 75 MHz): δ [ppm]=170.91, 154.88, 146.23, 136.13, 124.52, 122.22, 61.17, 55.83, 42.59, 14.28. HRMS: m/z=196.0967 (C_10_H_14_NO_3_ (M+H)^+^, calcd.: 196.0968).

**Ethyl 2‐(5‐methoxypyridin‐2‐yl)‐2‐((2,4,6‐trimethoxybenzyl)thio) acetate (39)**: Under argon atmosphere, lithium hexamethyldisilazide (4.36 mL, 1.3 M in THF, 5.67 mmol, 1.4 equiv) was added to a stirred solution of ethyl 2‐(5‐methoxypyridin‐2‐yl)acetate (790 mg, 4.05 mmol) in 40 mL anhydrous THF over the course of 25 min at −78 °C. After 30 min of stirring, thiosulfonate **38** (1.64 g, 4.46 mmol, 1.1 equiv) in 37 mL anhydrous THF was added at −78 °C within 100 min. The reaction mixture was allowed to gradually warm to room temperature over the course of 30 min and a saturated solution of NH_4_Cl was added. The resultant mixture was extracted with EtOAc (3x) and the combined organic layers were dried over MgSO_4_, filtered, and concentrated under reduced pressure. The residue was purified by flash column chromatography on silica gel (cyclohexane/EtOAc=1 : 1 v/v) to afford **39** (1.47 g, 3.61 mmol, 89 %) as a white solid. ^1^H NMR (CD_3_CN, 300 MHz): δ [ppm]=8.15 (dd, *J*=3.0, 0.7 Hz, 1H), 7.43 (dd, *J*=8.7, 0.7 Hz, 1H), 7.27 (dd, *J*=8.7, 3.0 Hz, 1H), 6.17 (s, 2H), 4.75 (s, 1H), 4.12 (q, *J*=7.1 Hz, 2H), 3.83 (s, 3H), 3.78 (s, 3H), 3.76 (s, 6H), 1.19 (t, *J*=7.1 Hz, 3H). ^13^C NMR (CD_3_CN, 75 MHz): δ [ppm]=171.39, 161.69, 159.74, 155.98, 149.63, 137.36, 124.05, 122.02, 107.32, 91.52, 62.23, 56.37, 56.33, 55.95, 54.59, 25.03, 14.33. HRMS: m/z=408.1455 (C_20_H_26_NO_6_S (M+H)^+^, calcd.: 408.1475).

**(*E*)‐2‐(5‐methoxypyridin‐2‐yl)‐2‐((2,4,6‐trimethoxybenzyl)thio)ethenol (40)**: Under argon atmosphere ester **39** (170 mg, 0.42 mmol) was dissolved in 2.1 mL anhydrous DCM and cooled to ‐94 °C. Diisobutylaluminium hydride (1.25 mL, 1.0 M in toluene, 1.25 mmol, 3.0 equiv) was added carefully via the vessel wall over the course of 1 h. The reaction mixture was stirred for an additional 15 min at −94 °C. Subsequently, a mixture of DCM/MeOH (1 mL, 3 : 1 v/v) was added via the vessel wall over the course of 30 min at −94 °C. The reaction mixture was stirred at room temperature for 10 min and a saturated solution of potassium sodium tartrate and DCM (20 ml each) was added. The resulting mixture was stirred at room temperature for 1 h and then extracted with DCM (3x). The combined organic layers were dried over MgSO_4_, filtered and concentrated. The residue was purified by recrystallization (MeOH/H_2_O) to afford the enol **40** (121 mg, 0.33 mmol, 80 %) as bright yellow crystals. ^1^H NMR (CD_3_CN, 300 MHz): δ [ppm]=14.83 (s, 1H), 8.07 (dd, J=3.0, 0.7 Hz, 1H), 7.79 (dd, J=9.1, 0.7 Hz, 1H), 7.45 (dd, J=9.1, 3.0 Hz, 1H), 7.15 (s, 1H), 6.11 (s, 2H), 3.86 (s, 3H), 3.76 (s, 3H), 3.70 (s, 2H), 3.61 (s, 6H). ^13^C NMR (CDCl_3_, 75 MHz): δ [ppm]=162.21, 161.52, 159.95, 154.14, 153.60, 132.01, 124.77, 122.72, 107.40, 102.62, 91.34, 56.68, 56.29, 55.97, 28.49. HRMS: m/z=364.1201 (C_18_H_22_NO_5_S (M+H)^+^, calcd.: 364.1213).

**Peptide Purification**: The desired peptides were purified by preparative HPLC on a C18‐column with a binary mixture of A (0.1 % TFA, 1 % ACN, 98.9 % H_2_O) and B (0.1 % TFA, 1 % H_2_O, 98.9 % ACN) as mobile phase (flow=6.0 mL/min). For model peptides a linear gradient from 3–30 % B was used. For other peptides a linear gradient was used as indicated. All products were isolated as white solids after lyophilization. Yields were determined by measurements of UV absorption as described in chapter 5.3. of the Suppl. Inf. or by weighing.

### Synthesis of Model Peptide Thioesters

**LYRAA‐S(CH_2_)_2_(CO)‐Gly‐NH_2_ (19A)**: Thioester **19A** was synthesized according to the published procedure.^[6]^ UPLC‐MS: t_R_=1.39 min (3–30 % B in 4 min); m/z=737.5 (C_32_H_53_N_10_O_8_S (M+H)^+^, calcd.: 737.4), 369.3 (C_32_H_54_N_10_O_8_S (M+2H)^2+^, calcd.: 369.2); C_32_H_52_N_10_O_8_S (MW=736.4 g mol^−1^).

**LYRAL‐S(CH_2_)_2_(CO)‐Gly‐NH_2_ (19L)**: Thioester **19L** was synthesized according to the published procedure.^[6]^ UPLC‐MS: t_R_=3.57 min (3–35 % B in 6 min); m/z=779.7 (C_35_H_59_N_10_O_8_S (M+H)^+^, calcd.: 779.4), 390.4 (C_35_H_60_N_10_O_8_S (M+2H)^2+^, calcd.: 390.2); C_35_H_58_N_10_O_8_S (MW=778.4 g mol^−1^).

### Synthesis of Model Peptide Selenoesters

**LYRAA‐SePh (22A)**: Selenoester **22A** was synthesized according to the published procedure.^[6]^ UPLC‐MS: t_R_=5.31 min (3–30 % B in 6 min); m/z=733.4 (C_33_H_49_N_8_O_6_Se (M+H)^+^, calcd.: 733.3), 367.4 (C_33_H_50_N_8_O_6_Se (M+2H)^2+^, calcd.: 367.1); C_33_H_48_N_8_O_6_Se (MW=732.3 g mol^−1^).

**LYRAL‐SePh (22L)**: Selenoester **22L** was synthesized according to the published procedure.^[6]^ UPLC‐MS: t_R_=2.31 min (3‐60 % B in 4 min); m/z=775.4 (C_36_H_55_N_8_O_6_Se (M+H)^+^, calcd.: 775.3), 388.3 (C_36_H_56_N_8_O_6_Se (M+2H)^2+^, calcd.: 388.2); C_36_H_54_N_8_O_6_Se (MW=774.3 g mol^−1^).

**General Procedure for Introduction of MMPyE Auxiliary**: The peptidyl‐resin was allowed to swell in MeOH/NMP/AcOH (6 : 4 : 0.02 v/v/v) for 15 min and then treated with a mixture of the enol **40** and NaCNBH_3_ (15 equiv each, c=0.4 M) in MeOH/NMP/AcOH (3 : 1 : 0.02 v/v/v) over night at room temperature (note: for N‐terminal glycine, shorter reaction times are recommended to avoid dialkylation). Afterwards the resin was washed with DCM (5x), MeOH (5x) and DCM (5x) and dried under vacuum. Finally, the peptide was deprotected and cleaved of the resin by addition of a mixture of TFA : TIS : H_2_O (95 : 2.5 : 2.5 v/v/v, 3 mL/10 μmol peptide). After 18–24 h the cleavage cocktail was collected by filtration and the resin was washed with TFA (3×0.5 mL). The combined filtrates were concentrated and ice cold Et_2_O (∼8–10‐fold volume) was added. The suspension was centrifuged (4000 rpm, 15 min, 4 °C) and the ether phase was decanted. For HPLC purification, the peptide pellet was dissolved in H_2_O : ACN : TFA (1 : 1 : 0.001 v/v/v).

***p*** **MPyE‐NRAEYSGLG (18N)**: Synthesis of **16N** was achieved by microwave‐assisted solid phase peptide synthesis (see chapter 5, Supporting Information, loading 10.0 μmol). The auxiliary was introduced according to the published procedure for the MPyE auxiliary^[6]^ and cleaved from the resin with TFA/H_2_O/TIS (95 : 2.5 : 2.5 v/v/v) for 18 h. After analyzing the resulting fractions with UPLC‐MS, the auxiliary peptide containing solutions were swiftly frozen for lyophilization to avoid decomposition of the product. Yield: 7.69 mg, 53 %. Note: **18N** was stored as dry lyophilizate at −80 °C to avoid auxiliary cleavage (Figure S17D). UPLC‐MS: t_R_=2.87 min (3–30 % B in 6 min); m/z=1102.6 (C_47_H_72_N_15_O_14_S (M+H)^+^, calcd.: 1102.5), 552.1 (C_47_H_73_N_15_O_14_S (M+2H)^2+^, calcd.: 551.8); C_47_H_71_N_15_O_14_S (MW=1102.2 g mol^−1^).

**MMPyE‐NRAEYSGLG (41N)**: Yield: 6.55 μmol, 66 %. UPLC‐MS: t_R_=3.71 min (3–30 % B in 6 min); m/z=1132.6 (C_48_H_74_N_15_O_15_S (M+H)^+^, calcd.: 1132.5), 566.9 (C_48_H_75_N_15_O_15_S (M+2H)^2+^, calcd.: 566.8); C_48_H_73_N_15_O_15_S (MW=1132.3 g mol^−1^).

**MMPyE‐VRAEYSGLG (41V)**: Yield: 6.15 μmol, 41 %. UPLC‐MS: t_R_=4.33 min (3–30 % B in 6 min); m/z=1117.7 (C_49_H_77_N_14_O_14_S (M+H)^+^, calcd.: 1117.6), 559.4 (C_49_H_78_N_14_O_14_S (M+2H)^2+^, calcd.: 559.3); C_48_H_74_N_14_O_13_S (MW=1117.3 g mol^−1^).

**Ligation of Model Peptides with Peptide Thioester**: To the ligation buffer (200 mM sodium hydrogen phosphate, 6 M Gdn HCl, 20 mM TCEP, pH=7.5) 3 vol.% thiophenol was added and the mixture was transferred to a lyophilized mixture of auxiliary peptide (1 equiv) and peptide thioester (1.5 equiv) to a final concentration of 5 mM auxiliary peptide. An argon atmosphere was applied to the reaction vessel. To monitor the progress of the reaction, aliquots were withdrawn from the ligation mixture, quenched with an aqueous solution of 0.1 % TFA or 0.1 % TFA, 2.5 % hydrazine, 30 mM TCEP and analyzed by UPLC‐MS. The progress of the ligation reaction was assessed by integration of the corresponding peak areas. After completion of the ligation, hydrazine and TCEP were added to the ligation solution. Ligated peptides showed partial rearrangement back to the thioester intermediate via *N* → *S* acyl transfer due to the acidic conditions used during and shortly after purification. Swift lyophilization after purification minimizes rearrangement. Alternatively, peptide solutions containing high amounts of rearranged thioesters can be treated with a solution of NH_4_Ac (0.1 M, pH=5) to a final pH of 5 to retrieve the amide.

**Ala‐Asn Ligation on pMPyE (20AN)**: 2 μmol of *para*‐MPyE peptide **18N** and 3 μmol of peptide thioester **19A** were allowed to react. The mixture was analyzed via UPLC‐MS after t=5 min and t=24 h (see Figure 2).

**Ala‐Asn Ligation on MMPyE (42AN)**: 2 μmol of MMPyE‐peptide **41N** and 3 μmol of peptide thioester **19A** were allowed to react for 72 h. Yield: 1.02 μmol, 51 %. UPLC‐MS: t_R_=4.04 min (3–30 % B in 6 min); m/z=854.0 (C_75_H_118_N_24_O_20_S (M+2H)^2+^, calcd.: 853.4), 569.9 (C_75_H_118_N_24_O_20_S (M+3H)^3+^, calcd.: 569.3); C_75_H_116_N_24_O_20_S (MW=1705.9 g mol^−1^).

**Leu‐Asn Ligation on MMPyE (42LN)**: 2 μmol of MMPyE‐peptide **41N** and 3 μmol of peptide thioester **19L** were allowed to react for 72 h. Yield: 1.14 μmol, 57 %. UPLC‐MS: t_R_ = 5.00 min (3–30 % B in 6 min); m/z=875.0 (C_78_H_124_N_24_O_20_S (M+2H)^2+^, calcd.: 874.5), 583.9 (C_78_H_125_N_24_O_20_S (M+3H)^3+^, calcd.: 583.3); C_78_H_122_N_24_O_20_S (MW=1748.0 g⋅mol^−1^).

**Ala‐Val Ligation on MMPyE (42AV)**: 2 μmol of MMPyE‐peptide **41V** and 3 μmol of peptide thioester **19A** were allowed to react for 72 h. Yield: 1.20 μmol, 60 %. UPLC‐MS: t_R_=6.07 min (3–30 % B in 8 min); m/z=846.7 (C_76_H_121_N_23_O_19_S (M+2H)^2+^, calcd.: 845.9), 565.1 (C_76_H_122_N_23_O_19_S (M+3H)^3+^, calcd.: 564.3); C_76_H_119_N_23_O_19_S (MW=1691.0 g mol^−1^).

**Leu‐Val Ligation on MMPyE (42LV)**: 2 μmol of MMPyE‐peptide **41V** and 3 μmol of peptide thioester **19L** were allowed to react for 120 h. Yield: 0.58 μmol, 29 %. UPLC‐MS: t_R_=5.86 min (3–35 % B in 6 min); m/z=867.5 (C_76_H_127_N_23_O_19_S (M+2H)^2+^, calcd.: 866.9), 578.9 (C_76_H_128_N_23_O_19_S (M+3H)^3+^, calcd.: 578.3); C_76_H_125_N_23_O_19_S (MW=1733.0 g mol^−1^).

**Ligation with Model Peptide Selenoester**: A lyophilized mixture of MMPyE peptide (1 equiv) and peptide selenoester (2 equiv) was dissolved in the ligation buffer (200 mM sodium hydrogen phosphate, 6 M Gdn HCl, 100 mM TCEP, 28 mM diphenyldiselenide (DPDS), pH=6.2) to a final concentration of 5 mM auxiliary peptide. An argon atmosphere was applied to the reaction vessel. To monitor the progress of the reaction, aliquots were withdrawn from the ligation mixture, quenched with an aqueous solution of 0.1 % TFA or 0.1 % TFA, 2.5 % hydrazine, 30 mM TCEP and analyzed by UPLC‐MS. The progress of the ligation reaction was assessed by integration of the corresponding peak areas. After completion of the ligation, hydrazine and TCEP were added to the ligation solution.

**Ala‐Asn Ligation on MMPyE (→42AN) with Selenoester 22A**: 1 μmol of MMPyE‐peptide **41N** and 2 μmol of peptide selenoester **22A** allowed to react for 24 h. Yield: 0.73 μmol, 73 %. UPLC‐MS: t_R_=4.05 min (3–30 % B in 6 min); m/z=854.4 (C_75_H_118_N_24_O_20_S (M+2H)^2+^, calcd.: 853.4), 570.1 (C_75_H_119_N_24_O_20_S (M+3H)^3+^, calcd.: 569.3); C_75_H_116_N_24_O_20_S (MW=1705.9 g mol^−1^).

**Leu‐Val Ligation on MMPyE (→42LV) with Selenoester 22A**: 0.5 μmol of MMPyE‐peptide **41V** and 1.0 μmol of peptide selenoester **22L** were allowed to react for 72 h. Yield: 0.24 μmol, 48 %. UPLC‐MS: t_R_=5.01 min (3–40 % B in 6 min); m/z=867.9 (C_76_H_127_N_23_O_19_S (M+2H)^2+^, calcd.: 866.9), 579.1 (C_76_H_128_N_23_O_19_S (M+3H)^3+^, calcd.: 578.3); C_76_H_125_N_23_O_19_S (MW=1733.0 g mol^−1^).

**Auxiliary Cleavage from Model Ligation Products**: Lyophilized ligation products were dissolved in the auxiliary cleavage mixture (0.5 M TCEP, 2 M morpholine, 5 mM MnCl_2_, 0.5 M 2‐acetylpyridine, pH=8.5) to a final concentration of 1 mM in plastic tubes. The closed lid was punctured to allow supply of oxygen. Note: Hydrazine is a known oxygen scavenger and auxiliary removal does not tolerate the presence of hydrazine.

**Removal of Auxiliary from Ligation Product AN (51AN)**: 500 nmol of ligated peptide **42AN** was incubated in the cleavage mixture for 3 h. Yield: 250 nmol, 50 %. UPLC‐MS: t_R_=4.44 min (3 % B for 1 min, then 3–30 % B in 6 min); m/z=770.7 (C_67_H_108_N_22_O_20_ (M+2H)^2+^, calcd.: 770.4), 514.2 (C_67_H_109_N_22_O_20_ (M+3H)^3+^, calcd.: 513.9); C_67_H_106_N_22_O_20_ (MW=1538.8 g mol^−1^).

**Removal of Auxiliary from Ligation Product AV (51AV)**: 500 nmol of ligated peptide **42AV** was incubated in the cleavage mixture for 6 h. Yield: 180 nmol, 36 %. UPLC‐MS: t_R_=3.85 min (then 3–30 % B in 6 min); m/z=763.2 (C_68_H_111_N_21_O_19_ (M+2H)^2+^, calcd.: 762.9), 509.2 (C_68_H_112_N_21_O_19_ (M+3H)^3+^, calcd.: 508.9); C_68_H_109_ N_21_O_19_ (MW=1523.8 g mol^−1^).

**Removal of Auxiliary from Ligation Product LN (51LN)**: 260 nmol of ligated peptide **42LN** was incubated in the cleavage mixture for 3 h. Yield: 104 nmol, 40 %. UPLC‐MS: t_R_=5.16 min (3 % B for 1 min, then 3–30 % B in 6 min); m/z=791.7 (C_70_H_114_N_22_O_20_ (M+2H)^2+^, calcd.: 791.4), 528.1 (C_70_H_115_N_22_O_20_ (M+3H)^3+^, calcd.: 527.9); C_70_H_112_N_22_O_20_ (MW=1580.8 g mol^−1^).

**Removal of Auxiliary from Ligation Product LV (51LV)**: 300 nmol of ligated peptide **42AN** was incubated in the cleavage mixture for 6 h. Yield: 109 nmol, 36 %. UPLC‐MS: t_R_=4.14 min (3–30 % B in 6 min); m/z=784.5 (C_71_H_117_N_21_O_19_ (M+2H)^2+^, calcd.: 783.9), 523.1 (C_71_H_118_N_21_O_19_ (M+3H)^3+^, calcd.: 523.0); C_71_H_115_N_21_O_19_ (MW=1566.8 g mol^−1^).

### Synthesis of the MUC5AC Peptide

**EIQALEEENAQLEQENAALEEEIAQLEY‐(APTTSTTS)_4_ Thioester (P1‐Muc5AC_4_, 53)**: The peptide was synthesized on a rink amide resin pre‐loaded with Hnb‐(S*t*Bu)Cys‐Gly via automated microwave assisted peptide‐synthesis (see 5.1, SuppI. Inf.). The peptide resin was treated with H_2_N−OH ⋅ HCl (3 M in NMP : DCM=5 : 1 v/v, 2x 30 min, 3 ml), washed and deprotected with TFA : phenol : H_2_O : TIS (88 : 5 : 5 : 2 v/v/v/v) for 2 h. The precipitated crude peptide was shaken at 37 °C in an aqueous buffer (0.7 ml, 100 mM TCEP, 200 mM sodium hydrogen phosphate, 6 M Gdn HCl, pH=6.5) containing 10 vol.% MPA. The reactions was quenched with 10 vol.% TFA. Preparative HPLC: 15–35 % B in 40 min. Yield: 0.43 μmol, 31 %, UPLC: t_R_=5.95 min (15–35 % B in 8 min). The product was submitted to ligation without further characterization.

**MMPyE‐(APTTSTTS)_4_‐KIAQLKQKIQALKQENQQLEEENAALEY (Muc5AC_4_‐P4, 54)**: Synthesis was performed on a rink amide resin via automated microwave assisted peptide‐synthesis (see 5.1, Suppl. Inf., loading 1.35 μmol). For reductive alkylation 10 equiv **40** and 30 equiv NaCNBH_3_ were allowed to react for 24 h. Preparative HPLC: 15–35 % B in 40 min. Yield: 771 nmol, 57 %. UPLC: t_R_=4.27 min (15–35 % B in 8 min). The product was submitted to the ligation without further characterization.

**Ligation of 53 and 54 (→55)**: Peptide thioester **53** (315 nmol) and auxiliary peptide **54** (210 nmol) were dissolved in ligation buffer (50 mM TCEP, 100 mM MPAA, 200 mM sodium hydrogen phosphate, 6 M Gdn HCl, pH=6.5) and allowed to react for 120 h. Yield: 53 nmol, 25 %. UPLC: t_R_=5.87 min (15–35 % B in 8 min). The product was submitted to auxiliary cleavage without further characterization.

**P1‐Muc5AC_8_‐P4 (56)**: Ligation product **55** (18 nmol) was incubated in the cleavage mixture for 4 h. Preparative HPLC: 15–35 % B in 40 min. Yield: 9.5 nmol, 53 %. UPLC: t_R_=6.6 min (15–35 % B in 8 min); HRMS: m/z=1786.9876 (C_522_H_862_N_139_O_214_ (M+11H)^7+^, calcd.: 1786.7282), 1563.5361 (C_522_H_863_N_139_O_214_ (M+11H)^8+^, calcd.: 1563.5131), 1389.9659 (C_522_H_864_N_139_O_214_ (M+11H)^9+^, calcd.: 1389.9013), 1251.0593 (C_522_H_865_N_139_O_214_ (M+11H)^10+^, calcd.: 1251.0119), 1137.4254 (C_522_H_866_N_139_O_214_ (M+11H)^11+^, calcd.: 1137.3751), 1042.6994 (C_522_H_867_N_139_O_214_ (M+12H)^12+^, calcd.: 1042.6778).

### Synthesis of hexaphosphorylated CTD peptide 60

**Synthesis of phosphorylated MMPyE‐CTD Peptide 57**: Synthesis was performed on a Rink amide resin via MW‐SPPS (see 5.1, Suppl. Inf., loading 10.0 μmol). For reductive alkylation 7 equiv **40** and 16 equiv NaCNBH_3_ were allowed to react for 18 h. The peptide was cleaved for 6 h with TFA:H_2_O:phenol:EDT (87.5 : 5 : 5 : 2.5 v/v/v/v, 2 mL/10 μmol peptide. (Note: TFA deprotection was stopped before complete removal of the trimethoxybenzyl (Tmb) protecting group to avoid formation of cleavage side‐product). Preparative HPLC: 3–40 % B in 40 min. Yield: 3.46 μmol, 35 %. UPLC‐MS: t_R_=3.45 min (3–30 % B in 4 min); m/z=1172.4, (C_104_H_148_N_23_O_37_S (M+3H)^3+^, calcd.: 1172.5), m/z=783.3, (C_104_H_148_N_23_O_37_S (M+4H)^4+^, calcd.: 783), C_104_H_150_N_23_O_46_P_3_S (MW=2343.01 g mol^−1^). UPLC‐MS: t_R_=2.53 min (3–30 % B in 4 min); m/z=862.0, (C_104_H_150_N_23_O_46_P_3_S (M+3H)^3+^, calcd.: 861.6), C_104_H_150_N_23_O_46_P_3_S (MW=2581.9 g mol^−1^).

**His_6_‐[(CH_2_)_2_O]_6_CH_2_CO‐(YpSPTSPS)_3_ Hydrazide (58)**: The peptide was assembled on a chlorotriyl resin pre‐loaded with Fmoc‐hydrazine via automated solid‐phase peptide synthesis (see 5.1 Suppl. Inf., loading 10.0 μmol). After introduction of the PEG_6_‐chain (Fmoc‐HN‐[(CH_2_)_2_O]_6_‐COOH (5 equiv), HATU (5.0 equiv, 0.3 M) and DIPEA (15 equiv) in DMF), capping and coupling of six histidines, the peptide was cleaved with TFA:H_2_O:phenol:EDT (87.5 : 5 : 5 : 2.5 v/v/v/v, 2 mL/10 μmol peptide) for 3 h. Preparative HPLC 3–40 % B in 40 min. Yield: 1.86 μmol, 19 %; **58**: 2.15 μmol, 22 %. UPLC‐MS (**58**): t_R_=2.08 min (3‐50 % B in 4 min); m/z=1197.3, (C_147_H_213_N_42_O_58_P_3_ (M+3H)^3+^, calcd.: 1196.8), 898.8 (C_147_H_210_N_42_O_49_ (M+4H)^4+^, calcd.: 897.9), 719.2 (C_147_H_210_N_42_O_49_ (M+5H)^5+^, calcd.: 718.5).

**Formation of Thioester 59, Ligation with 57 and Removal of MMPyE Group (60)**: Peptide hydrazide **58** (500 nmol, 1 equiv) was dissolved in H_2_O containing 200 mM MPAA and 6 M Gdn HCl (pH=3) and acetylacetone (1.5 equiv, 10 % in H_2_O) was added. After 1 h reaction at room temperature, the solution was transferred to a mixture of auxiliary peptide **57** (500 nmol, 1 equiv) in 100 mM TCEP, 200 mM Na_2_HPO_4_ and 6 M Gdn HCl (pH=8.5). NaOH (5 M) was added carefully by alternating addition over the vessel cap followed by vortexing to adjust the pH to 6.3. After 120 h, the ligation was quenched by the addition of 5 μl hydrazine solution (81 % in H_2_O) and 10 μl of TCEP (1 M in H_2_O) and the resulting mixture was subjected to an Amicon Ultra Mass Filter from *Merck Millipore* (0.5 ml, 3 kDa cut‐off) and washed twice. After lyophilization, the crude peptide mixture was dissolved in 100 μl phosphate buffer (0.2 M NaH_2_PO_4_, pH=7.4) and purified via a His‐Tag kit using Ni^2+^ Agarose‐NTA beads. The resulting peptide solution was lyophilized and used i without further purification. The peptide mixture obtained after lyophilization was dissolved in 200 μL of the auxiliary removal mixture (0.5 M TCEP, 2 M morpholine, 5 mM MnCl_2_, 0.5 M 2‐acetylpyridine, pH=8.5) for 24 h. Preparative HPLC: 3–40 % B in 40 min. Yield: 18 nmol, 4 % over 3 steps. UPLC‐MS: t_R_=5.47 min (5‐30 % B in 8 min); m/z=1195.1 (C_243_H_350_N_62_O_103_P_6_ (M+5H)^5+^, calcd.: 1195.1), 996.9 (C_243_H_350_N_62_O_103_P_6_ (M+6H)^6+^, calcd.: 996.0), m/z=854.1 (C_243_H_350_N_62_O_103_P_6_ (M+7H)^7+^, calcd.: 853.9) m/z=745.9 (C_243_H_350_N_62_O_103_P_6_ (M+8H)^8+^, calcd.: 747.3) m/z=664.3 (C_243_H_350_N_62_O_103_P_6_ (M+9H)^9+^, calcd.: 664.4), (C_243_H_350_N_62_O_103_P_6_ (MW=5970.3).

### Synthesis of head‐to‐tail cyclized peptide 65

**Synthesis of MMPyE‐peptide 62, Formation of Thioester 63 and Cyclization (64)**: Microwave assisted peptide‐synthesis was performed on a pre‐loaded chlorotrityl‐tentagel resin (see 5.1, Suppl. Inf., loading 10.0 μmol). For reductive alkylation 7 equiv of **40** and 16 equiv of NaCNBH_3_ were allowed to react for 18 h. Approx, 1.0 μmol MMPyE‐peptide **62** was cleaved from the resin with hexafluoroisopropanol/DCM (7 : 3 v/v) and thioester **63** was formed according to the general procedure (see 5.1, Suppl. Inf.). The peptide was cleaved for 18 h with TFA, H_2_O, phenol, thioanisole, EDT (82 : 5 : 5 : 5 : 3 v/v/v/v/v) and incubated in ligation buffer at 37 °C for 2 h. Preparative HPLC: 20–50 % B in 40 min. Yield: 89 nmol, 9 %. UPLC‐MS: t_R_=4.88 min (20–50 % B in 8 min); m/z=1138.2, (C_109_H_150_N_24_O_28_S (M+2H)^2+^, calcd.: 1138.0), 758.9 (C_109_H_151_N_24_O_28_S (M+3H)^3+^, calcd.: 759.0), C_109_H_148_N_24_O_28_S (MW=2274.55 g⋅mol^−1^).

**Cyclopeptide 65**: 55 nmol of ligation product **64** was incubated in the cleavage mixture for 12 h. Preparative HPLC: 20–50 % B in 40 min. Yield: 24 nmol, 44 %. UPLC‐MS: t_R_=3.93 min (20–50 % B in 8 min); m/z=1054.42 (C_101_H_141_N_23_O_27_ (M+2H)^2+^, calcd.: 1054.02), 703.01 (C_101_H_142_N_23_O_27_ (M+3H)^3+^, calcd.: 703.35), C_101_H_139_N_23_O_27_ (MW=2107.32). MALDI‐MS: m/z=2104 (C_101_H_140_N_23_O_27_ (M+H)^+^, calcd.: 2108).

More detailed procedures and characterization data is provided in the Supporting Information.

## Conflict of interest

The authors declare no conflict of interest.

1

## Supporting information

As a service to our authors and readers, this journal provides supporting information supplied by the authors. Such materials are peer reviewed and may be re‐organized for online delivery, but are not copy‐edited or typeset. Technical support issues arising from supporting information (other than missing files) should be addressed to the authors.

Supporting Information

## Data Availability

The data that support the findings of this study are available in the supplementary material of this article.
