# Supplementary material for: Enhancing Auxiliary‐Mediated Native Chemical Ligation at Challenging Junctions with Pyridine Scaffolds
Source: Chemistry. 2022 Oct 17;28(68):e202202065. doi: 10.1002/chem.202202065 (PMC10091703; doi:10.1002/chem.202202065)
Supplement: Supplementary file 1 — Supporting Information [file CHEM-28-0-s001.pdf]

Chemistry–A European Journal

Supporting Information

Enhancing Auxiliary-Mediated Native Chemical Ligation at Challenging Junctions with Pyridine Scaffolds

Sebastian Trunschke, Emanuele Piemontese, Olaf Fuchs, Skander Abboud, and Oliver Seitz*

Table of Contents

1. GENERAL INFORMATION 2

1.1 Materials and instruments 2

2. SYNTHESIS OF PRECURSOR FOR *P*MPYE AUXILIARY 3

3. SYNTHESIS OF PRECURSOR FOR MMPYE AUXILIARY 4

4. NMR SPECTROSCOPY 5

5. SYNTHESIS OF PEPTIDES 19

5.1 Fmoc-strategy 19
5.2 Boc-strategy for the Synthesis of Peptide Thioester 21
5.3 Determination of Peptide and Protein Concentration 22

6. SYNTHESIS OF PEPTIDE CHALKOESTER 23

6.1 Peptide Thioester 23
6.2 Peptide Selenoester 24

7. INTRODUCTION OF THE AUXILIARY 25

7.1 Introduction of the 2-Mercapto-2-(pyridin-4-yl)ethyl Auxiliary during SPPS 25
7.2 Introduction of the 2-Mercapto-2-(5-methoxypyridin-2-yl)ethyl Auxiliary during SPPS 26
7.3 Stability of MPyE- and MMPyE Auxiliary Peptides in Ligation Buffer and Acidic Solution 28

8. NATIVE CHEMICAL LIGATION 33

8.1 Ligation of *para*-MPyE- and MMPyE-Peptides with Peptide Thioesters 33
8.2 Ligation of MMPyE-Peptides with Peptide Selenoesters 37
8.3 Ligation of MMPyE-Peptides with Peptide Thioesters at Elevated Temperatures 39

9. AUXILIARY REMOVAL 42

9.1 Removal of the MMPyE Auxiliary 42
9.2 Comparison of Auxiliary Removal of MPyE- and MMPyE-Asn Peptide 47

10. SYNTHESIS OF P1-MUC5AC$_8$-P4 49

11. SYNTHESIS OF HIS$_6$-PEG$_6$-(Y*P*SPTSPS)$_6$ 53

12. SYNTHESIS OF CYCLOPEPTIDE 65 57

13. REFERENCES 61

1. General Information

1.1 Materials and instruments

Commercially available compounds were used without further purification. Dry solvents were taken from a *MBraun* Solvent Purification System *SPS 800*. Purification of compounds by flash chromatography was done on silica gel (0.060-0.2 mm, 60 Å) from *Acros Organics* using technical grade solvents. TLC silica gel plates *60 F254* from *Merck* were used for thin-layer chromatography. NMR-spectra were recorded on a *Bruker Avance II* 500 MHz Spectrometer and referenced to the residual protonated solvent signal. Elemental analysis was carried out on a *HEKAtech Eurovector3000*.

Preparative HPLC purifications were carried out by using an *Agilent 1100 Series* system and a *Nucleodur C18 Gravity* column (250 mm x 21 mm, 5 µm) from *Macherey-Nagel* with a binary mixture of A (0.1 % TFA, 1 % ACN, 98.9 % H_2O) and B (0.1 % TFA, 1 % H_2O, 98.9 % ACN) as a mobile phase (flow = 15 mL/min) in a linear gradient as described. For semi-preparative HPLC-purifications a Polaris C18-A column (250 x 10.0 mm) from *Varian* was used with a binary mixture of A (0.1 % TFA, 1 % ACN, 98.9 % H_2O) and B (0.1 % TFA, 1 % H_2O, 98.9 % ACN) as mobile phase (flow = 6.0 mL/min) in a linear gradient as described.

UPLC-MS measurements were performed by using an *Acquity* system from *Waters* and a *BEH130* C18 column (2.1 x 50 mm, 1.7 µm; heater set on 50 °C) with a binary mixture of A (0.1 % TFA, 1 % ACN, 98.9 % H_2O) and B (0.1 % TFA, 1 % H_2O, 98.9 % ACN) as a mobile phase (flow = 0.5 mL/min) in a linear gradient as described.

High-resolution ESI-MS measurements were performed on an Orbitrap XL-mass spectrometer from *Thermo Scientific* (Waltham, MA, USA) with a 1200-HPLC from *Agilent Technologies* using a hypersil 100-C18-column from *Thermo Scientific* with a binary mixture of A (0.1 % HCO_2H, 99.9 % H_2O) and B (0.1 % HCO_2H, 99.9 % ACN) as a mobile phase (flow = 1.3 mL/min) in a linear gradient from 5 - 100 % B. *Xcalibur* (*Thermo Scientific*) was used for the evaluation of the spectra.

2. Synthesis of Precursor for *p*MPyE Auxiliary

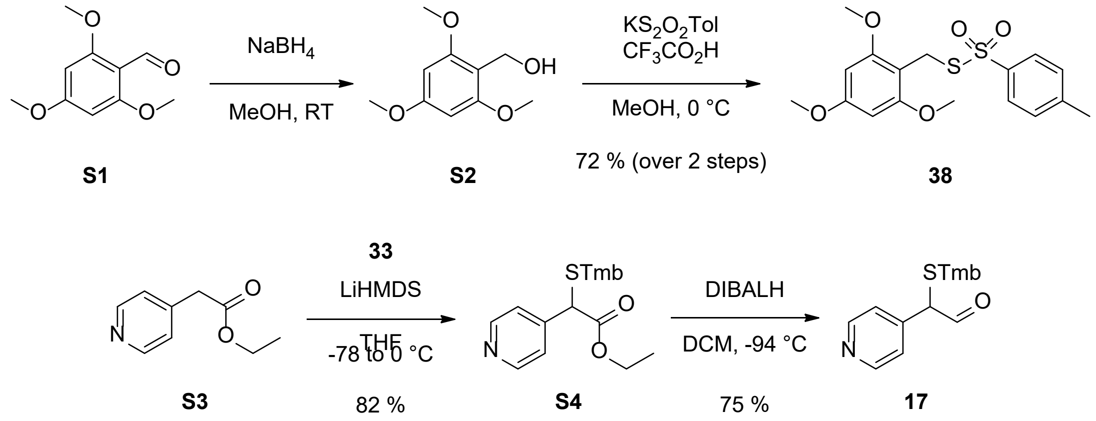

Scheme S1: Synthesis of thiosulfonate **38** in 2 steps and synthesis of aldehyde **17**, used for the introduction of *para*-MPyE group by reductive amination, in 2 steps.

S-2,4,6-trimethoxybenzyl 4-methylbenzenesulfonothioate (38)

Alcohol **S2** and thiosulfonate **38** were synthesized according to the published procedure.[1] The NMR data obtained for **38** is in agreement with the literature data.

^{1}H NMR ($CDCl_3$, 500 MHz): δ [ppm] = 7.85 (d, J = 8.3 Hz, 2H), 7.32 (d, J = 8.3 Hz, 2H), 6.01 (s, 2H), 4.29 (s, 2H), 3.77 (s, 3H), 3.69 (s, 6H), 2.45 (s, 3H).

^{13}C NMR ($CDCl_3$, 126 MHz): δ [ppm] = 161.57, 159.24, 144.07, 142.57, 129.56, 127.26, 102.63, 90.49, 77.41, 77.16, 76.90, 55.79, 55.47, 29.24, 21.72.

3. Synthesis of Precursor for MMPyE Auxiliary

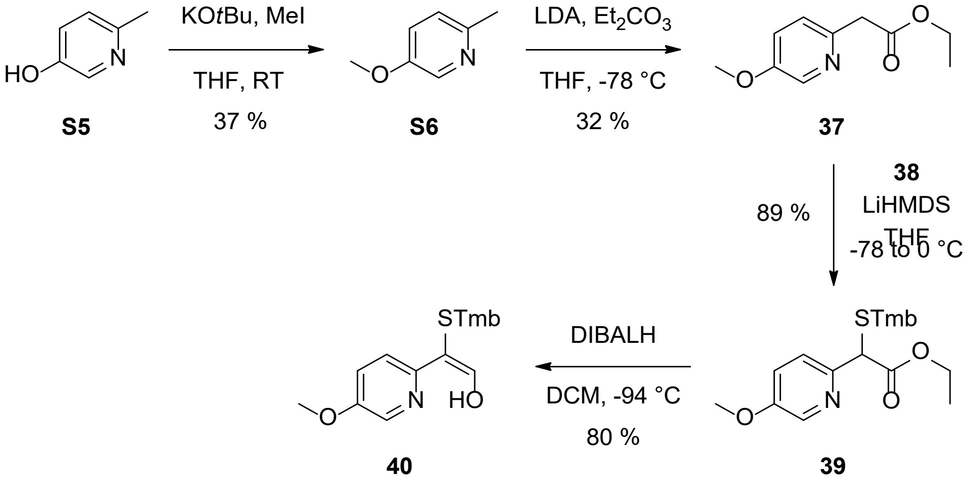

Scheme S2: Synthesis of enol **40** over 4 steps for the introduction of MMPyE group by reductive amination.

5-Methoxy-2-methylpyridine (S6)

Picoline **S6** was synthesized according to the published procedure.[2] The crude methyl ether was purified by fractionated distillation to give **S6** in 37 % yield. The NMR data obtained for **S6** is in agreement with the literature data.

^{1}H NMR ($CDCl_3$, 300 MHz): δ [ppm] = 8.17 (d, *J* = 2.9 Hz, 1H), 7.07 (m, 2H), 3.82 (s, 3H), 2.48 (s, 3H).
^{13}C NMR ($CDCl_3$, 75 MHz): δ [ppm] = 153.91, 150.21, 135.66, 123.63, 122.08, 55.77, 23.16.
HRMS: m/z = 124.0756 ($C_7H_{10}NO$ $(M+H)^+$, calcd.: 124.0757).

4. NMR Spectroscopy

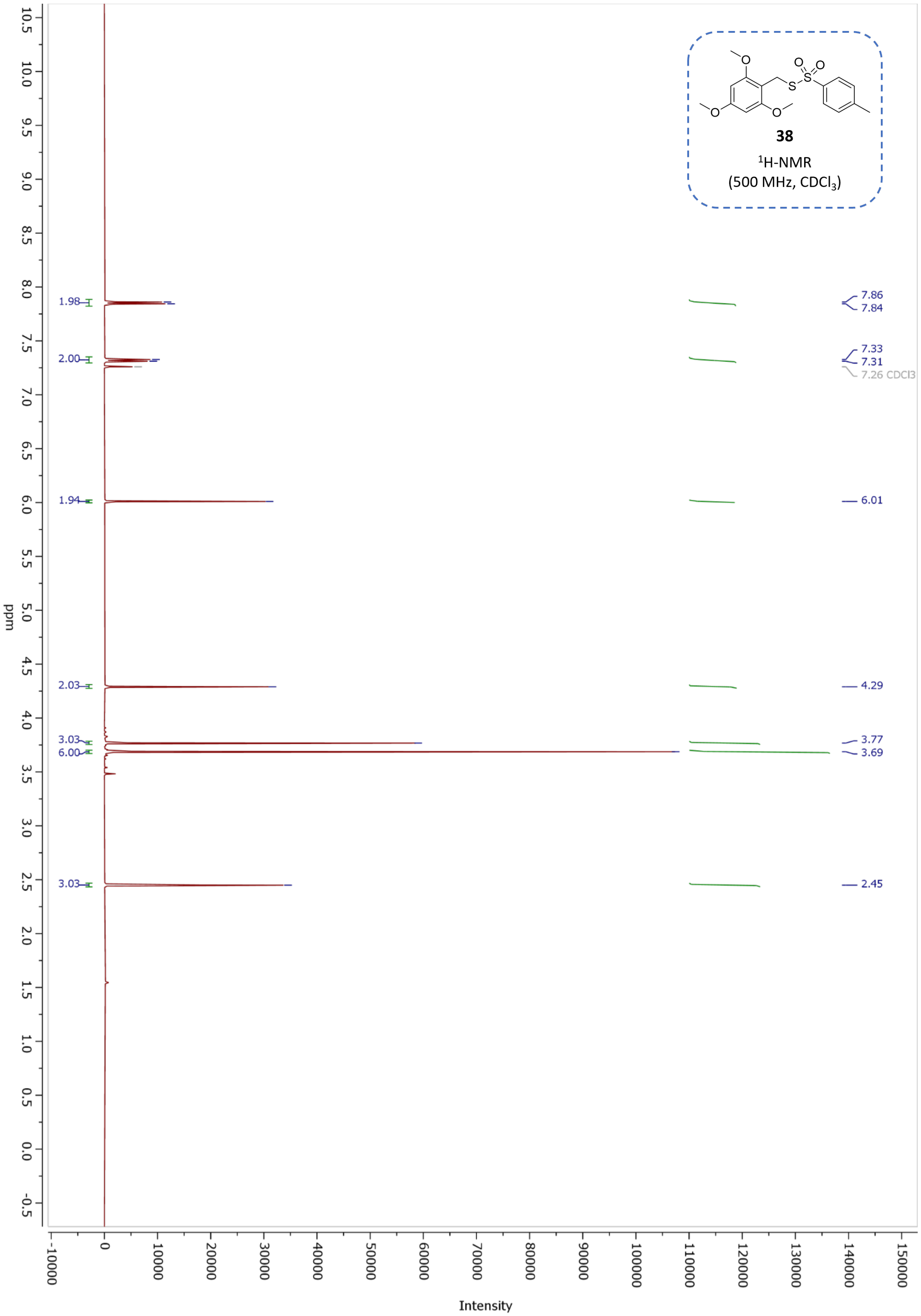

Figure S1: ^{1}H NMR spectrum (500 MHz) of thiosulfonate **38** in $CDCl_3$.

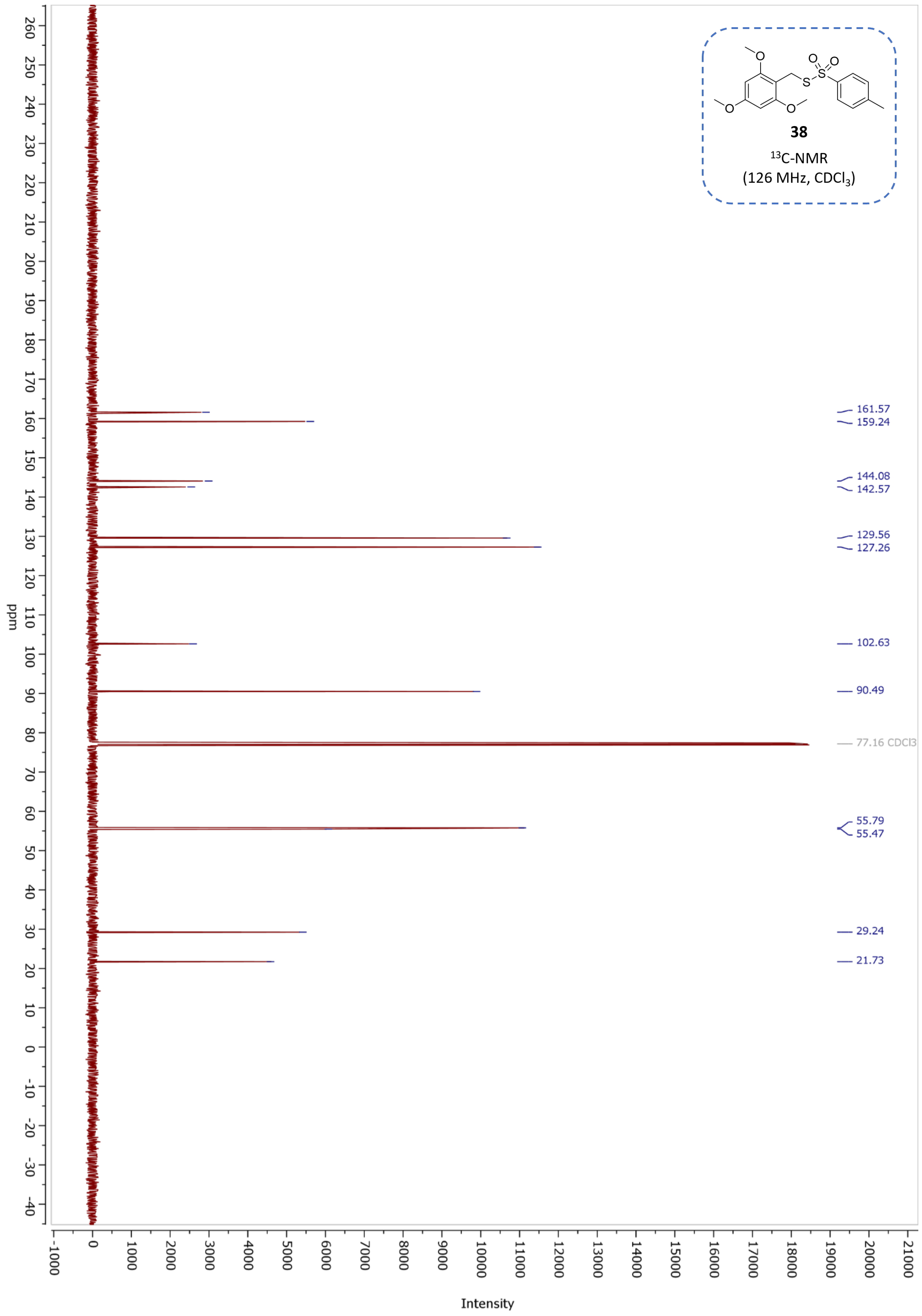

Figure S2: ^{13}C NMR spectrum (125 MHz) of thiosulfonate **38** in $CDCl_3$.

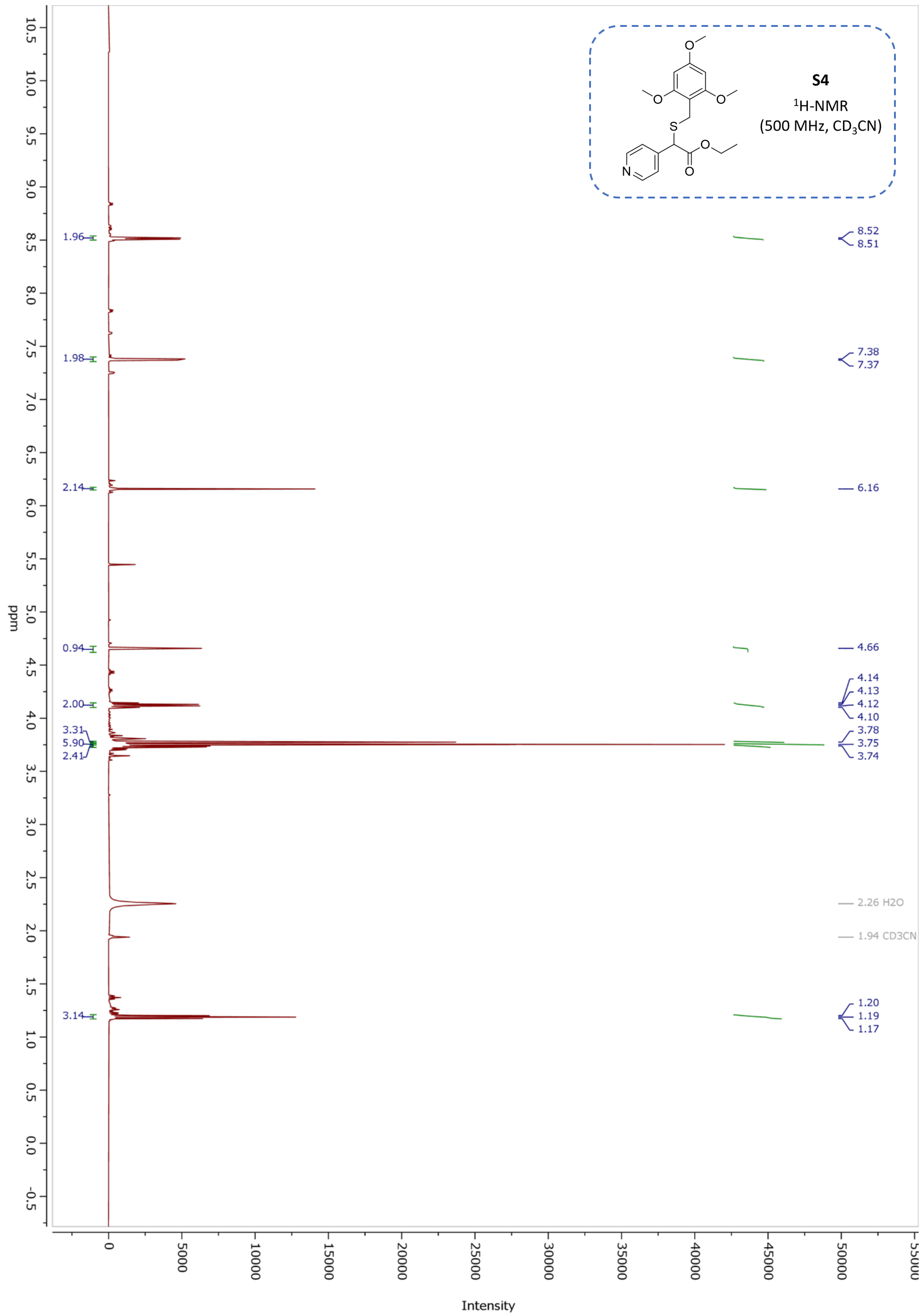

Figure S3: ^{1}H NMR spectrum (500 MHz) of ester **S4** in CD_3CN.

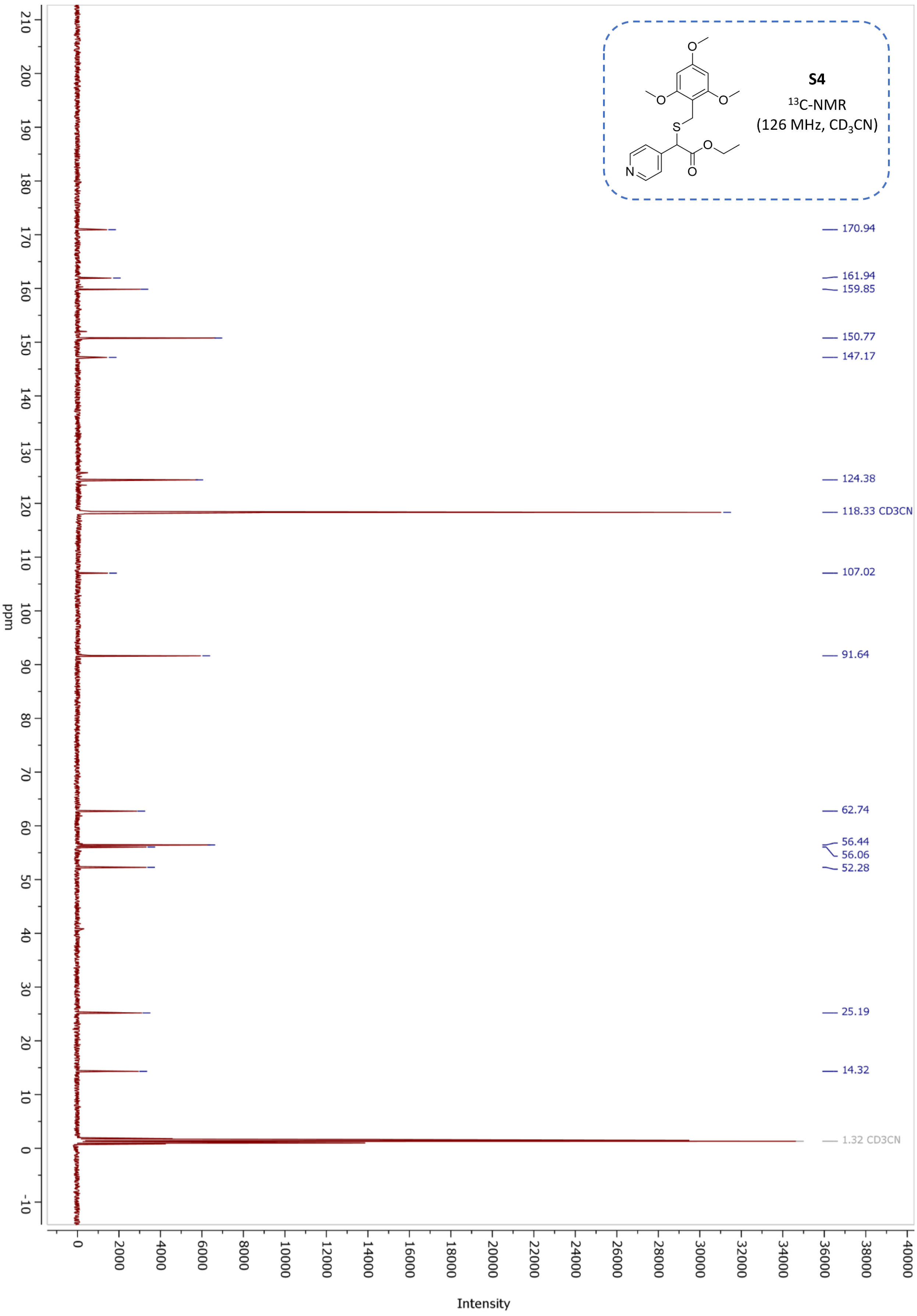

Figure S4: ^{13}C NMR spectrum (126 MHz) of ester **S4** in CD_3CN.

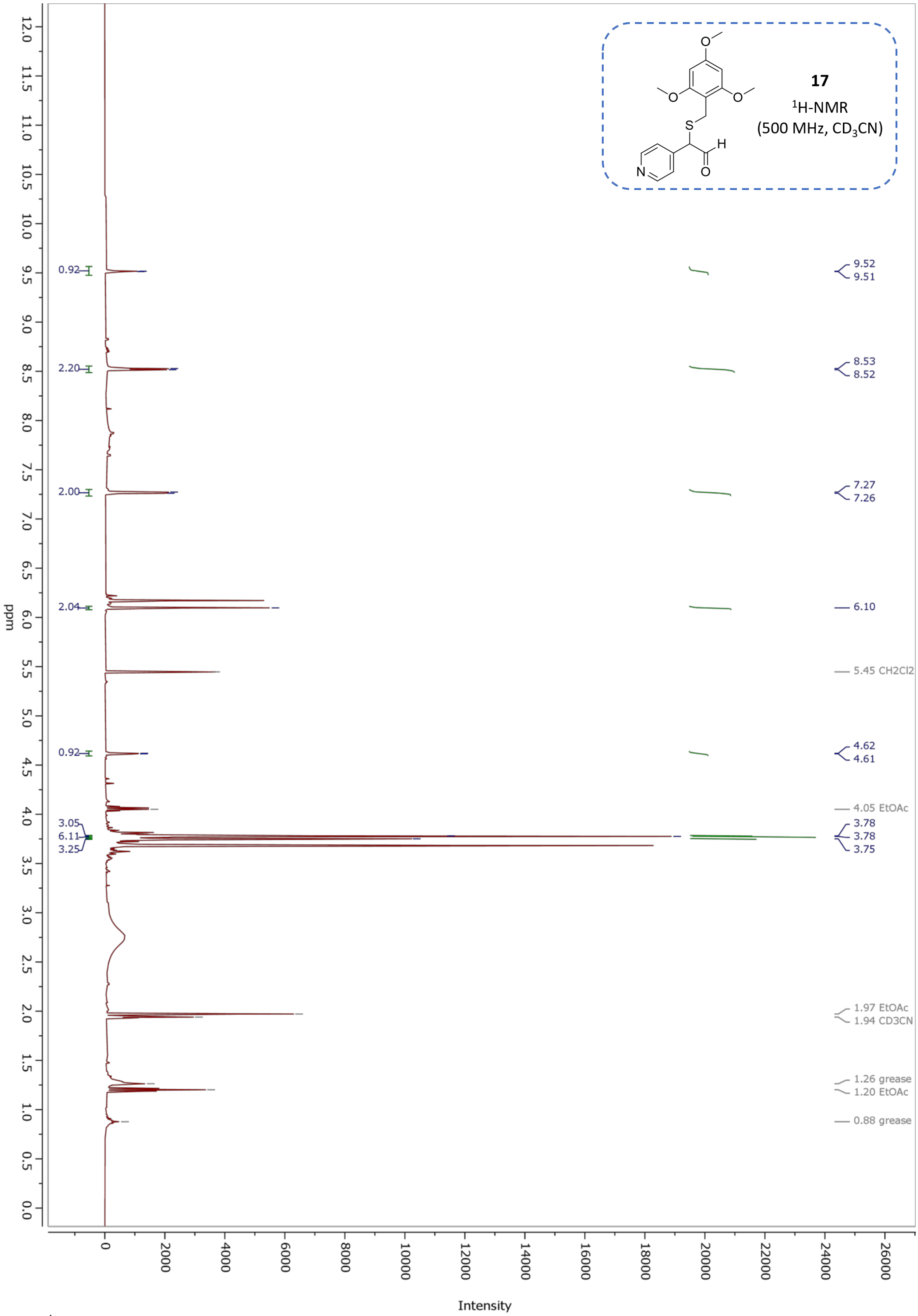

Figure S5: ^{1}H NMR spectrum (500 MHz) of aldehyde **17** in CD_3CN.

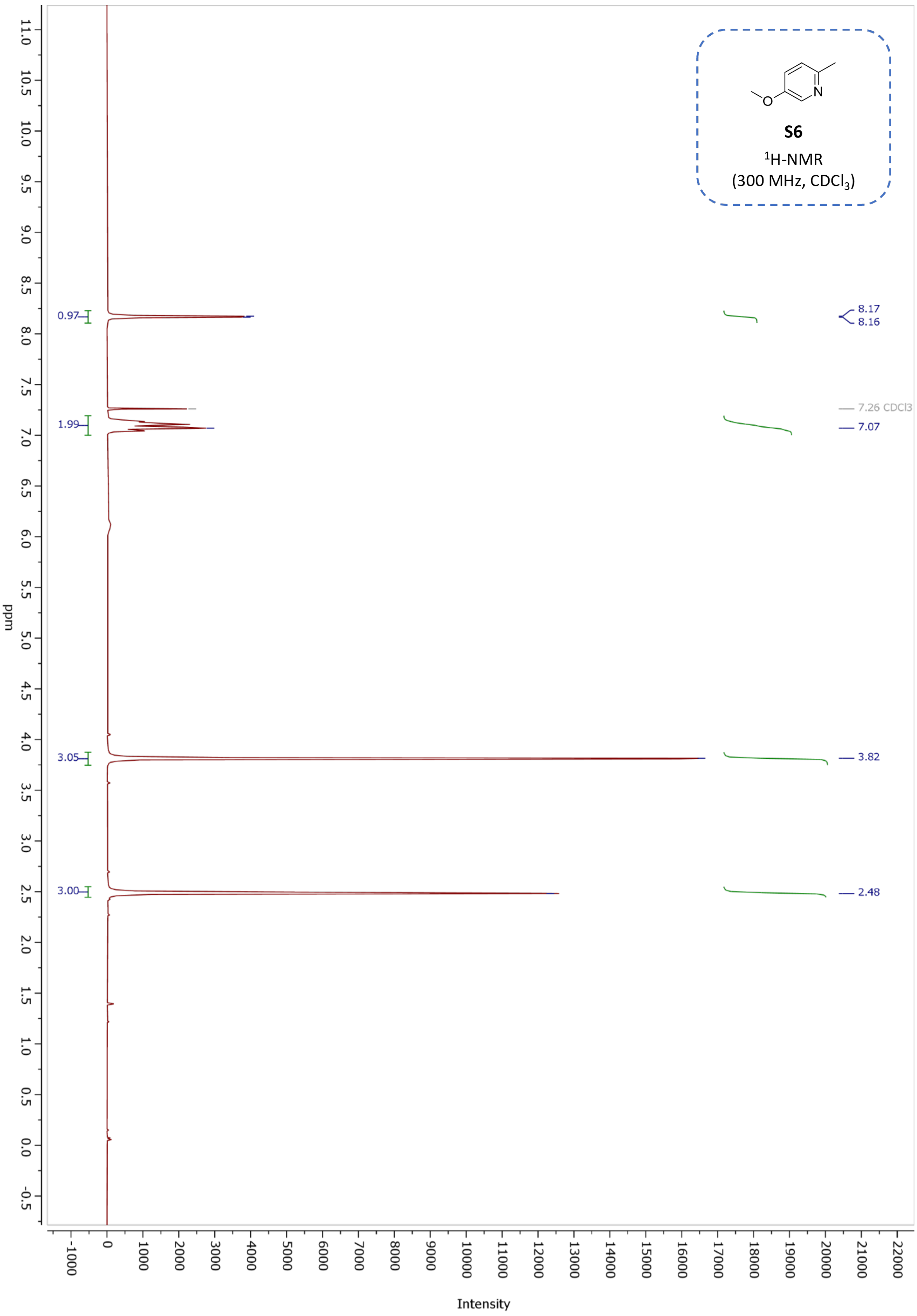

Figure S6: ^{1}H NMR spectrum (300 MHz) of 5-Methoxy-2-methylpyridine (**S6**) in $CDCl_3$.

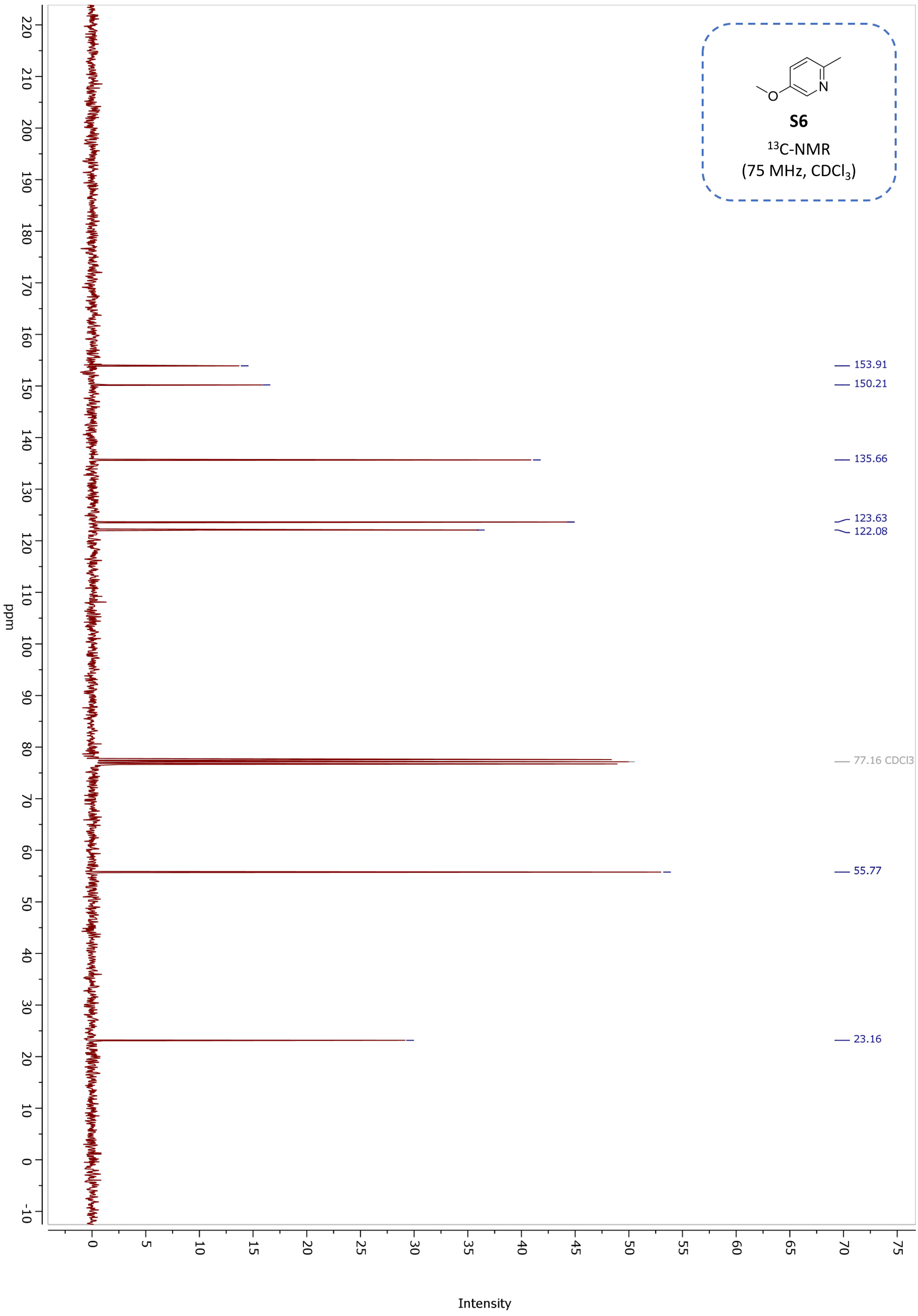

Figure S7: ^{13}C NMR spectrum (75 MHz) of 5-Methoxy-2-methylpyridine (**S6**) in $CDCl_3$.

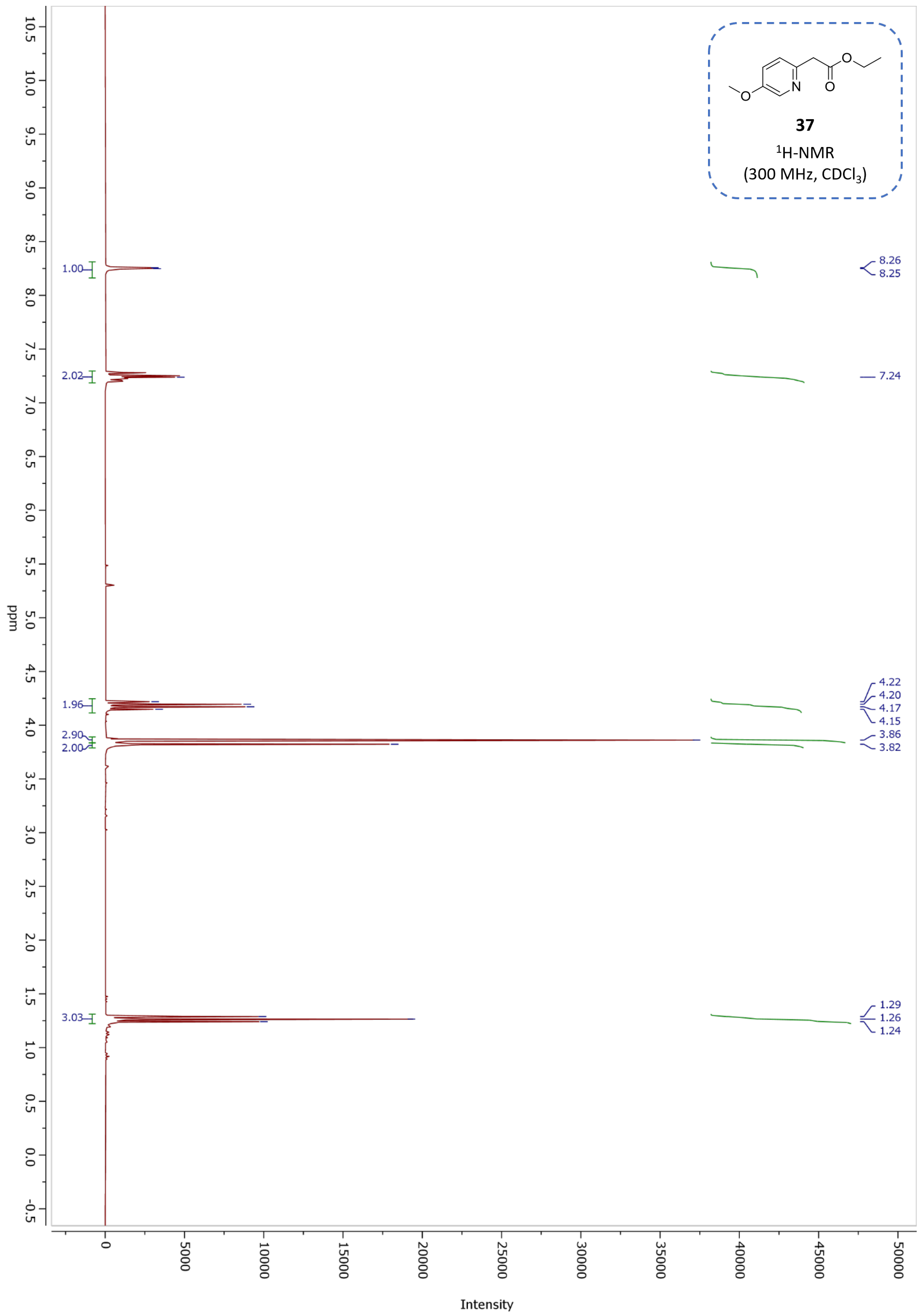

Figure S8: ^{1}H NMR spectrum (300 MHz) of ester **37** in $CDCl_3$.

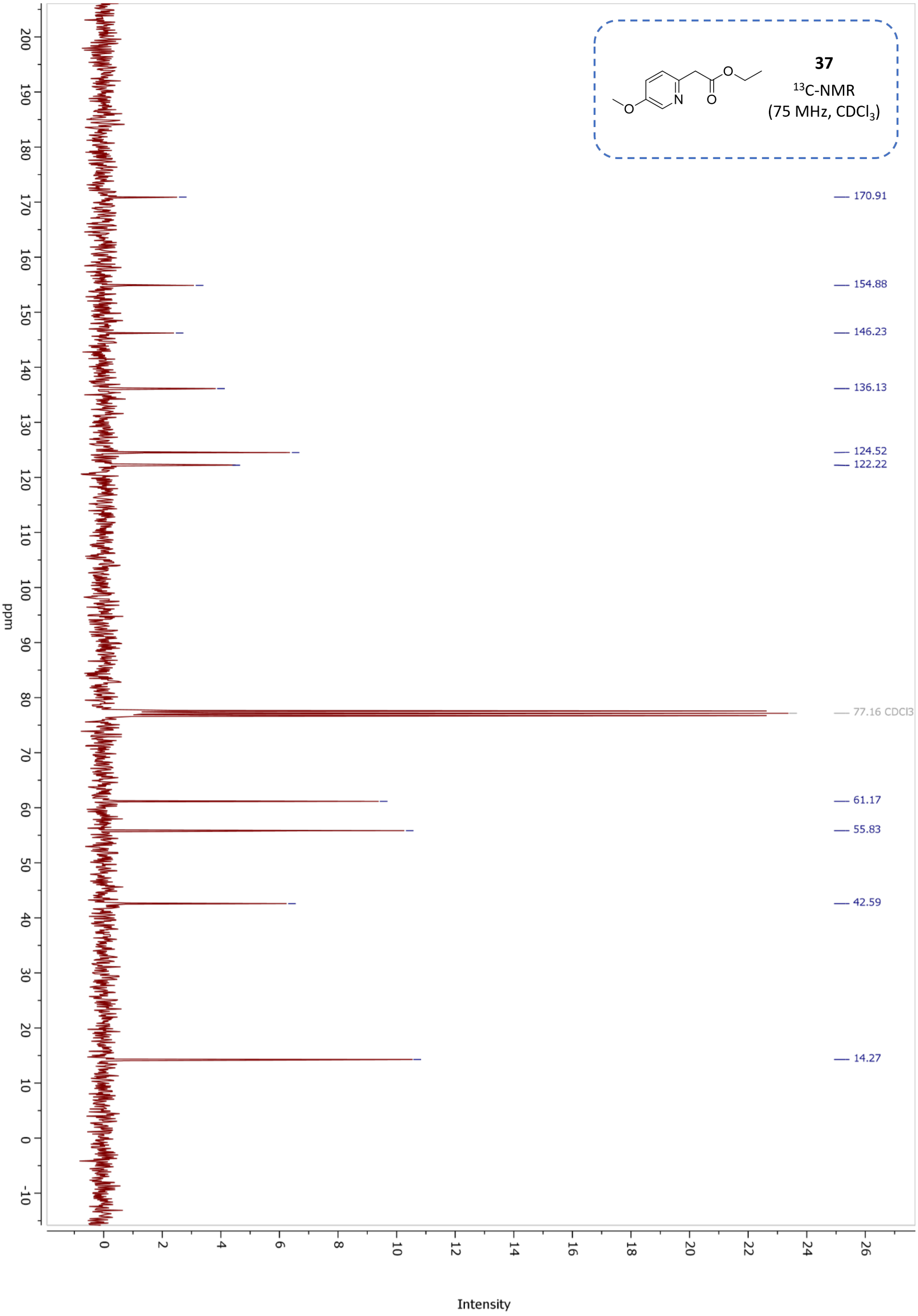

Figure S9: ^{13}C NMR spectrum (75 MHz) of ester **37** in $CDCl_3$.

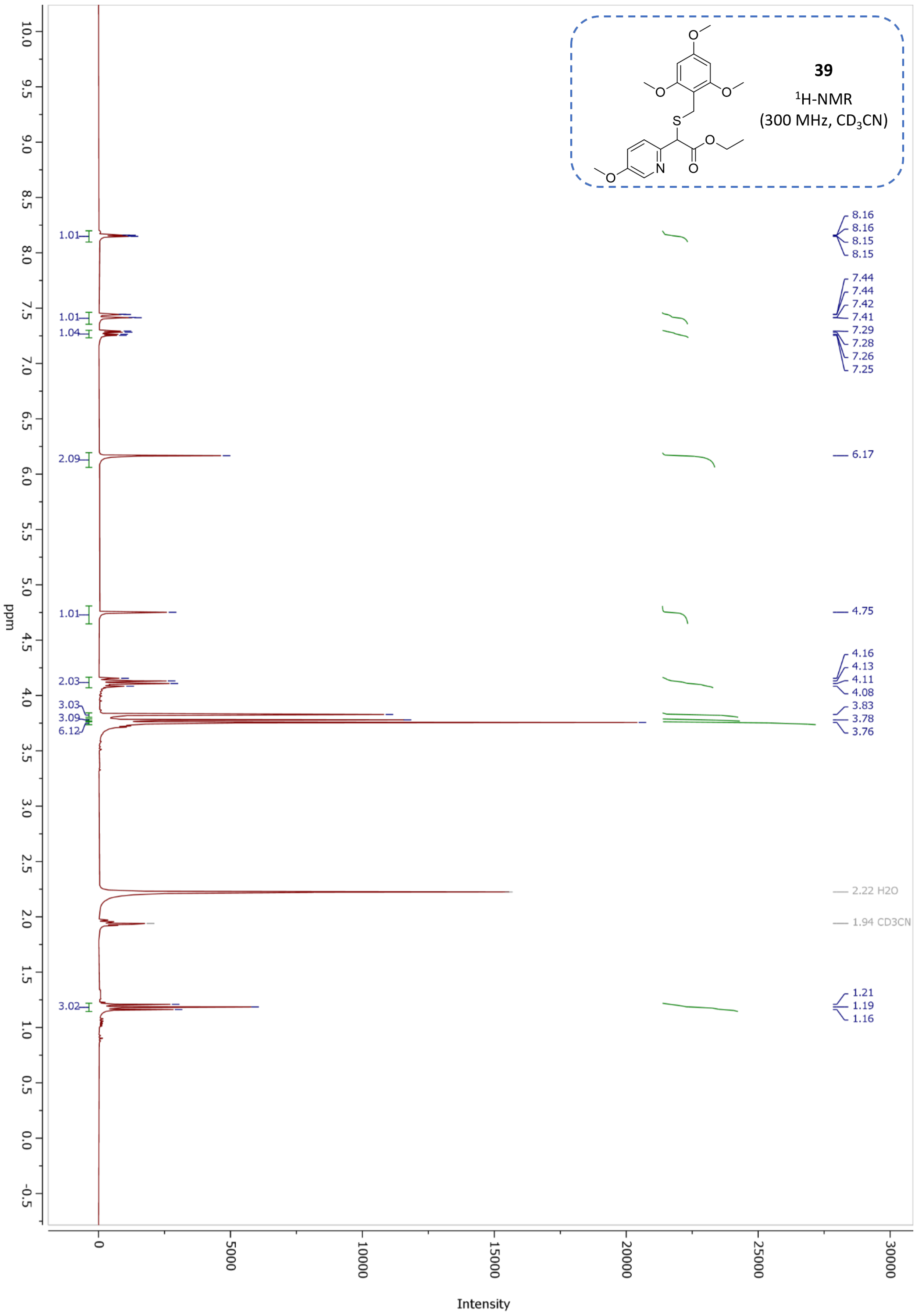

Figure S10: ^{1}H NMR spectrum (300 MHz) of ester **39** in CD_3CN.

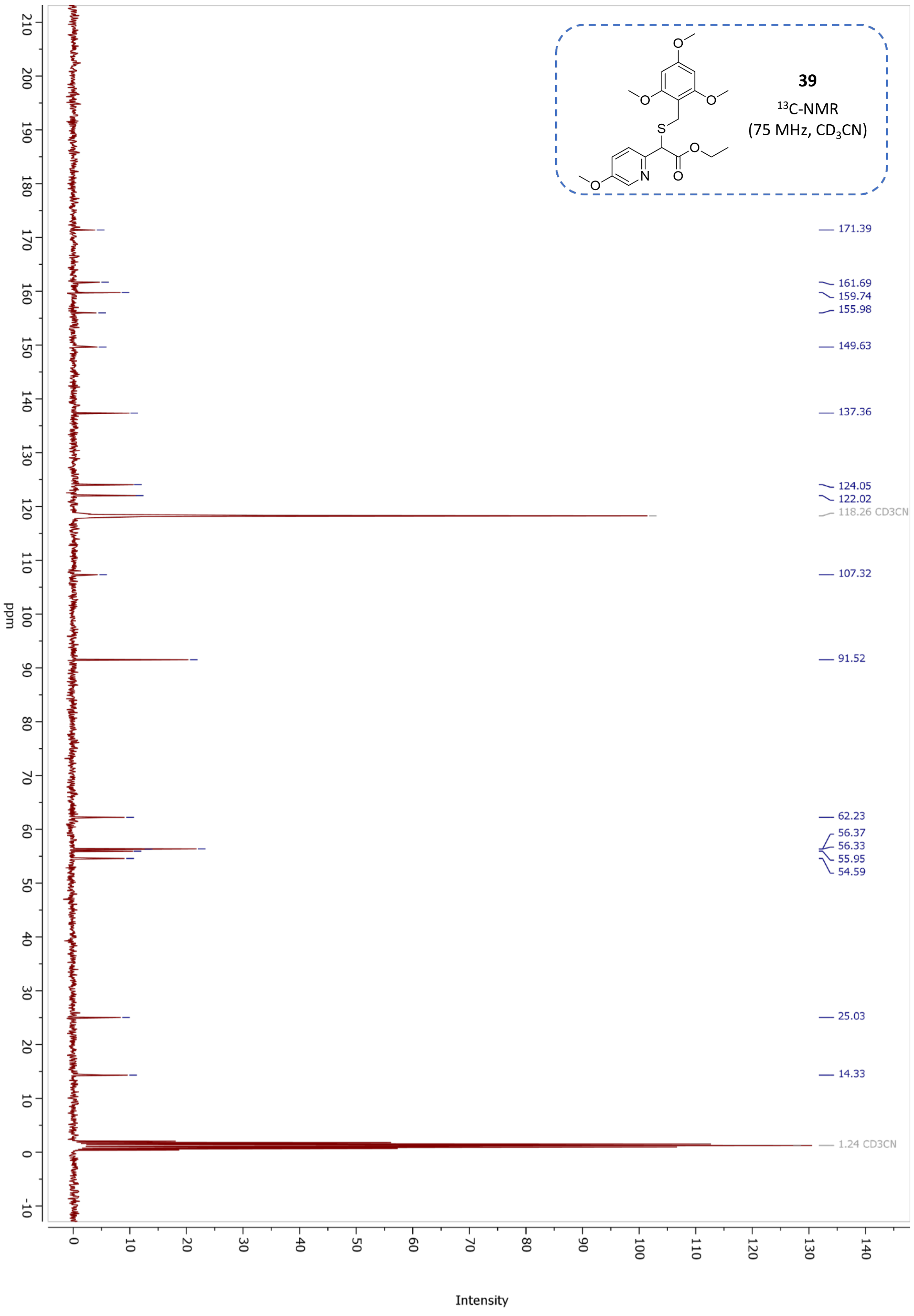

Figure S11: ^{13}C NMR spectrum (75 MHz) of ester **39** in CD_3CN.

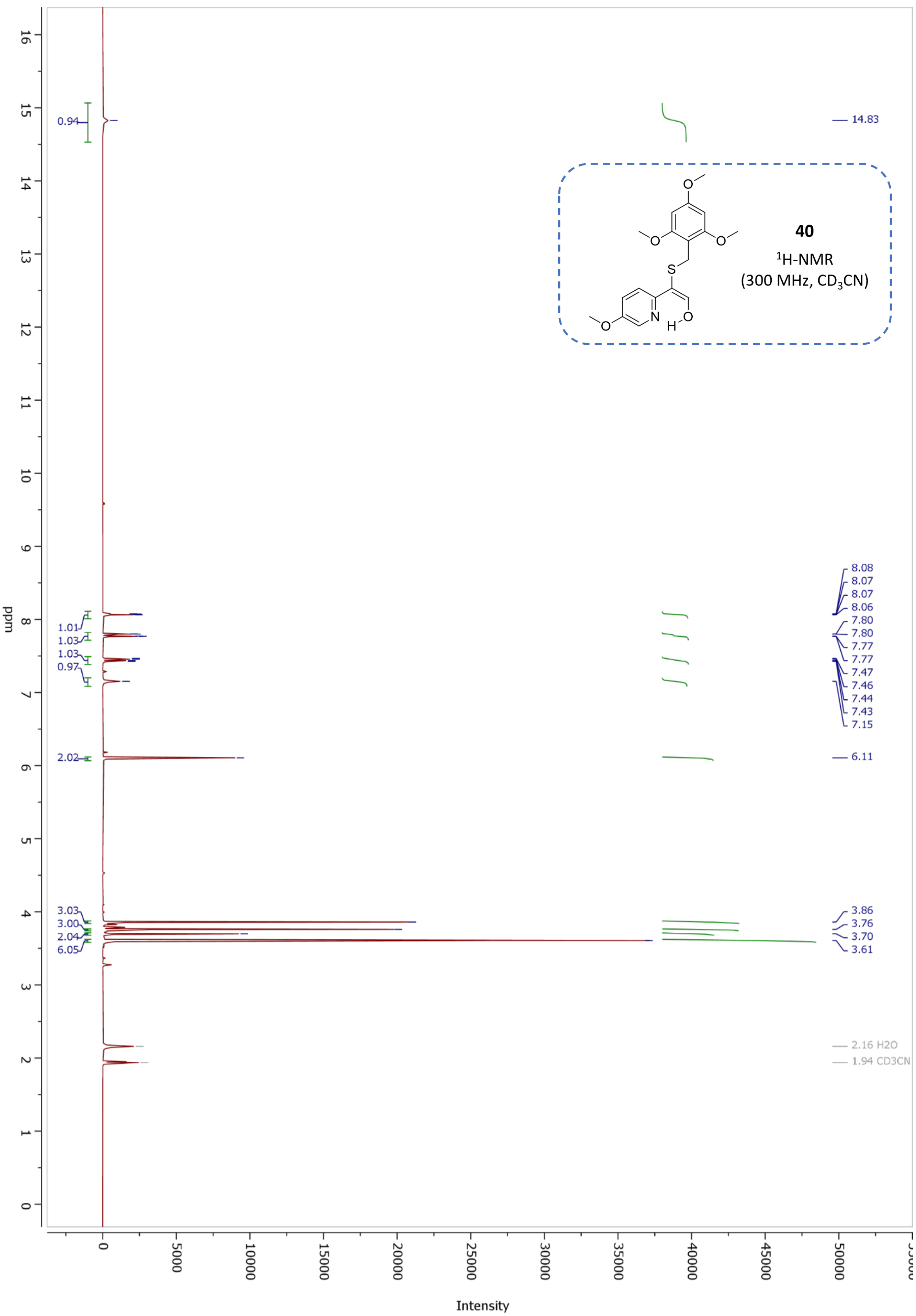

Figure S12: ^{1}H NMR spectrum (300 MHz) of enol **40** in CD_3CN.

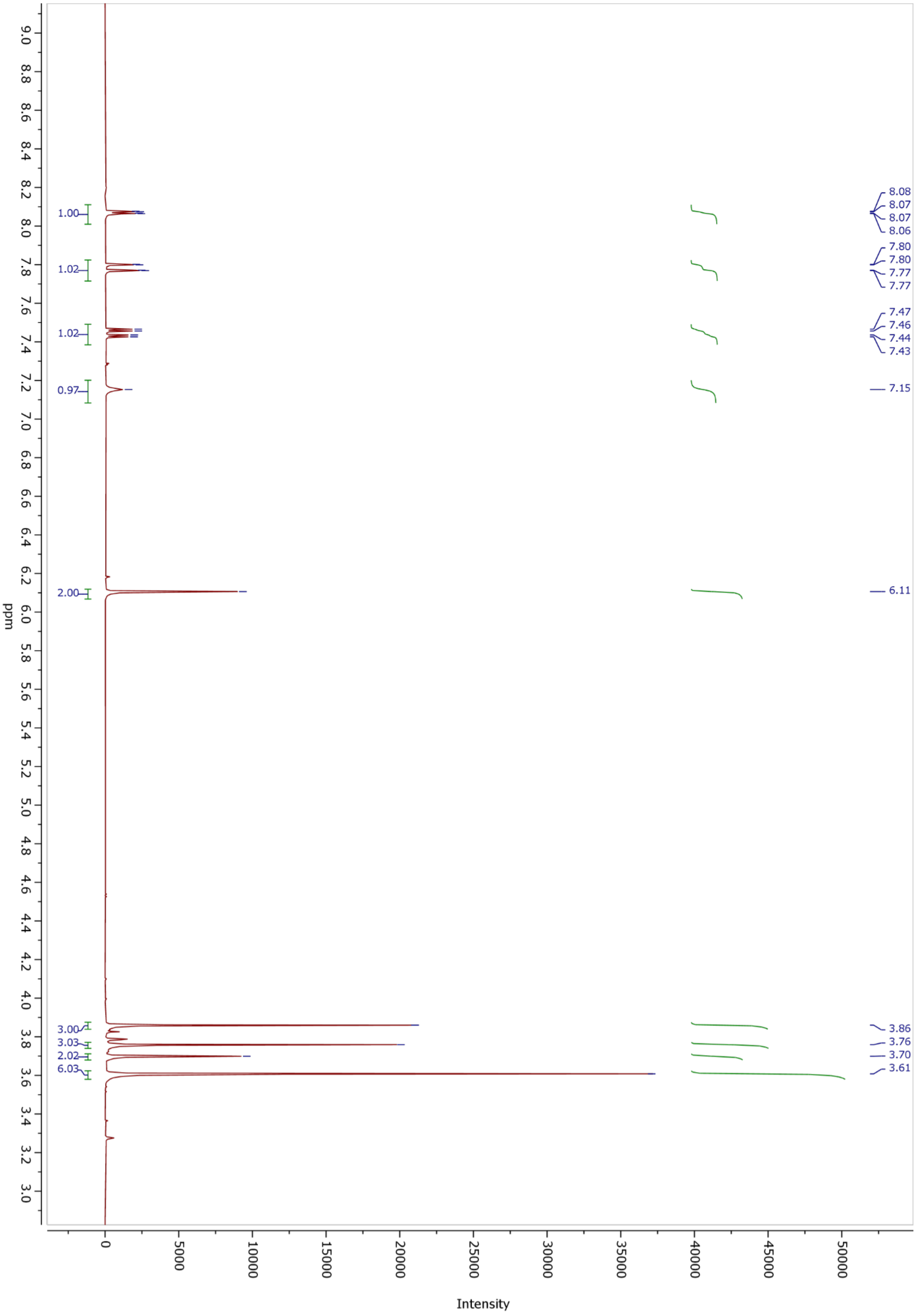

Figure S13: ^{1}H NMR spectrum (300 MHz) of enol **40** from 3.0 to 9.0 ppm in CD_3CN.

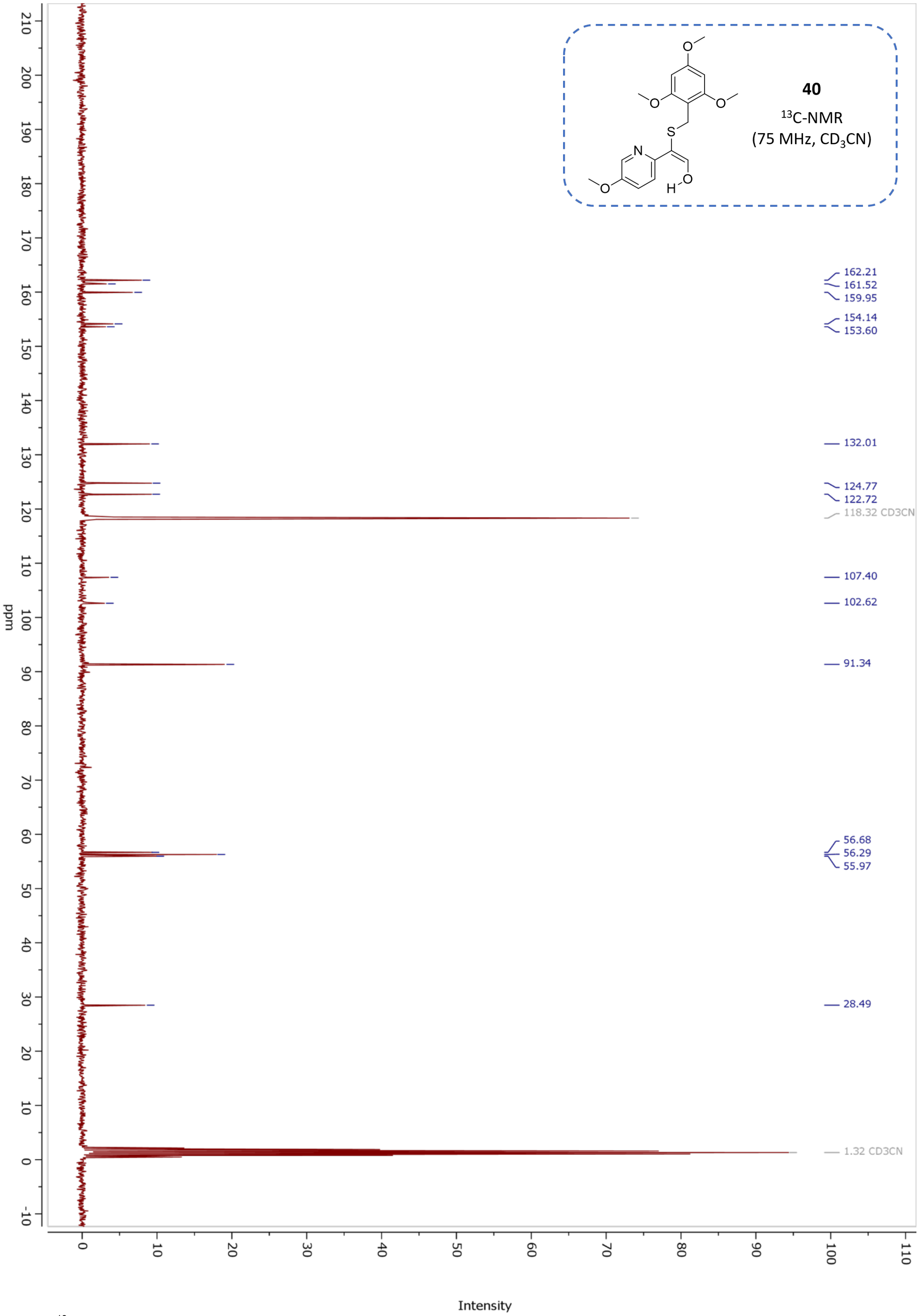

Figure S14: ^{13}C NMR spectrum (75 MHz) of enol **40** in CD_3CN.

5. Synthesis of peptides

5.1 Fmoc-strategy

Loading of Tentagel Rink Amide Resin

The resin (~0.18 µmol/mg) was transferred into a syringe equipped with a filter frit and swollen in DMF (10 min). The Fmoc-group was removed by treatment of the resin with a solution of 20 % piperidine in DMF (2x 5 min). The resin was washed (3x DMF, 3x DCM, 3x DMF) and the Fmoc-protected amino acid (4 eq., c = 0.2 M in DMF) coupled in presence of PyBOP (4 eq.) and DIPEA (12 eq.). After 1 h the resin was washed (3x DMF, 3x DCM, 3x DMF), acetylated with DMF:Ac_2O:Lutidine (89:5:6 v/v/v, 5 min) and then washed (3x DMF, 3x DCM, 3x DMF). The Fmoc-group was removed by treatment of the resin with a solution of 20 % piperidine in DMF (2x 5 min) and the initial loading estimated by quantification of the piperidine-fulvene adduct (λ = 301 nm, ε = 7800 M^{-1} cm^{-1}). Afterwards the resin was washed (3x DMF, 3x DCM, 3x DMF).

Loading of Tentagel Rink Amide Resin with Hnb-(S*t*Bu)Cys-Gly

The Fmoc-deprotected resin (~0.18 µmol/mg) was allowed to swell in DMF (10 min) and Fmoc-Gly-OH and Fmoc-(S*t*Bu)Cys-OH were coupled to the resin successively. Fmoc group was deprotected by two successive treatments with 20% piperidine in NMP respectively for 3 min and 5 min. The H-Cys(StBu)-Gly-Rink-resin was washed with 1:1 DMF/MeOH (4x) and 2-Hydroxy-5-nitrobenzaldehyde (10 equiv.) in DMF/MeOH/AcOH (1:1:0.01 v/v/v, 125 mM) was then added. After shaking for 5 min, the resin was washed with DMF/MeOH (1:1 v/v, 3x) and DMF (3x). A fresh solution of sodium borohydride (20 equiv.) in DMF (250 mM) was added immediately and the reactor was shaken for 20 min. The resin was washed with DMF (4x), treated with 20 vol.% piperidine in NMP (3 min, 3x) and washed again with NMP (3x), DCM (3x) and NMP (3x).

Loading of Tentagel 2-Chlorotrityl Resin

The resin (~0.17 µmol/mg) was transferred into a syringe equipped with a filter frit and allowed to swell in anhydrous DCM (10 min). Hydrolysed trityl residues were reactivated by addition of $SOCl_2$ in anhydrous DCM (1:9 v/v) for 1 h. The resin was washed (3x anhydrous DCM, 3x DIPEA in anhydrous DCM (1:19 v/v), 3x anhydrous DCM) and the Fmoc-protected amino acid (5 eq., c = 0.4 M in anhydrous DCM) was coupled in presence of DIPEA (20 eq.). After 1 h the resin was washed (10x DCM), capped with a solution of DIPEA (50eq.) in MeOH:DCM (1:3 v/v) for 10 min and then washed again (5x DCM, 3x DMF). The Fmoc-group was removed by treatment of the resin with a solution of 20 % piperidine in DMF (2x 5 min) and the initial loading estimated by the UV-absorbance of the piperidine-fulvene adduct (λ = 301 nm, ε = 7800 M^{-1} cm^{-1}). Afterwards the resin was washed (3x DMF, 3x DCM, 3x DMF).

Loading of Tentagel 2-Chlorotrityl Resin with Hydrazide Peptides

Reactivated trityl resin was allowed to swell in DCM:DMF (1:1 v/v, 30 min) and successively treated with Fmoc-Hydrazine (10 eq.) in DCM:DMF (1:1 v/v) in presence of DIPEA (20 eq.). After 24 h the resin was washed (10x DCM), capped with a solution of DIPEA (50eq.) in MeOH:DCM (1:3 v/v) for 10 min and then washed again (5x DCM, 3x DMF). The Fmoc-group was removed by treatment of the resin with a solution of 20 % piperidine in DMF (2x 5 min), the initial loading estimated and the resin was washed (3x DMF, 3x DCM, 3x DMF).

Automated Solid Phase Peptide Synthesis was performed by using a MultiPep RS from *Intavis*. Amino acids were dissolved in NMP. Automated SPPS was executed using the following protocol:

- Coupling: Fmoc-protected amino acids (4 eq.) were activated with HCTU/OxymaPure (8 eq. each, c = 0.6 M) and transferred to the resin with NMM (24 eq.) (coupling time = 30 min, 2x). For Fmoc-Ser(PO_3BzlH)-OH, activation was conducted with HOBt (0.6 M) and DIPEA (24 eq.).
- Capping: The resin was treated with DMF:Ac_2O:lutidine (89:5:6 v/v/v) for 5 min.
- Deprotection: The resin was treated with 20 % piperidine in DMF for 1x 8 min and 1x 6 min.
- Final cleavage: The resin was washed with DCM and dried under vacuum. Then a mixture of TFA:TIS:H_2O (95:2.5:2.5 v/v/v, 3 mL/10 µmol peptide) was added to the resin. After 2 h the cleavage cocktail was collected by filtration, the resin was washed with TFA (3x 0.5 mL) and the combined filtrates were concentrated (~ 1 mL) under argon flow.
- Peptide precipitation: To the remaining residue cold Et_2O (~ 8-10-fold volume) was added and the suspension was centrifuged (4000 rpm, 15 min). Afterwards the ether phase was decanted. The remaining peptide pellet was dissolved in H_2O:ACN:TFA (1:1:0.001 v/v/v) and purified by preparative HPLC as indicated.

Microwave-assisted Solid Phase Peptide Synthesis was performed by using an Initiator+ Alstra from *Biotage*. All amino acids were dissolved in NMP. Automated SPPS was executed using the following protocol:

- *Coupling:* Fmoc-protected amino acids, DIC and OxymaPure (2.5 eq. each, c = 0.1 M) were transferred to the resin and coupled for 5 min at 75 °C or 10 min at 40 °C for Fmoc-protected His, Cys and Arg.
- *Capping:* Acetic anhydride (1 ml, 5 M in DMF) and DIPEA (2.5 ml, 2 M in NMP) were mixed and transferred to the resin (capping time = 10 min).
- *Deprotection:* The resin was treated with 20 % piperidine in DMF for 1x 3 min and 1x 5 min.
- *Final cleavage:* The resin was washed with DCM and dried under vacuum. Then a mixture of TFA:TIS:H_2O (95:2.5:2.5 v/v/v, 3 mL/10 µmol peptide) was added to the resin. After 2 h the cleavage cocktail was collected by filtration, the resin was washed with TFA (3x 0.5 mL) and the combined filtrates were concentrated (~ 1 mL) under argon flow.
- *Peptide precipitation:* To the remaining residue ice cold Et_2O (~ 8-10-fold volume) was added and centrifuged (4000 rpm, 15 min, 4 °C). Afterwards the ether phase was decanted. The remaining peptide pellet was dissolved in H_2O:ACN:TFA (1:1:0.001 v/v/v) and purified by preparative HPLC as indicated.

Thioester Formation after SPPS on Trityl Resin

After final Fmoc-deprotection the resin was treated with a solution of Boc-anhydride (50 eq., c = 0.4 M) and DIPEA (10 eq.) in DMF for 1 h to protect the N-terminal amino group. The peptides were cleaved from the trityl resin by treatment with 30 vol.% HFIP in DCM (2 mL/10 µmol peptide) for 2 h and the cleavage solution was collected by filtration and transferred into a round bottom flask. The resin was washed with DCM (2x 1 mL) and the combined filtrates were concentrated in vacuo. To the resulting residue, a mixture of *N*,*N'*-diisopropylcarbodiimide (30 eq., c = 0.15 M), OxymaPure (30 eq.) and DIPEA (40 eq.) in DMF was added. Thiophenol (30 eq.) was added and the mixture was heated to 50 °C under an argon atmosphere. After 1 h the solvent was reduced at high vacuum and ice cold Et_2O (~8-10-fold volume) was added. The suspension was centrifuged (4000 rpm, 15 min, 4 °C) and the ether phase decanted. The remaining peptide pellet was air dried and a mixture of

TFA:Phenol:H_2O:Thioanisole:EDT (82:5:5:5:3 v/v/v/v/v, 2 ml/10 µmol peptide) was added to remove the protecting groups. After 1 h the cleavage cocktail was reduced under a stream of Argon. Ice cold Et_2O (~8-10-fold volume) was added to the remaining solution, the suspension centrifuged (4000 rpm, 15 min, 4 °C) and the ether decanted. Afterwards the precipitate was suspended a second time in ether, centrifuged, decanted, and the pellet dissolved in H_2O:ACN:TFA (1:1:0.001 v/v/v) and purified by preparative HPLC as indicated.

Selenoester Formation after SPPS on 2-Chlorotrityl Resin

After final Fmoc-deprotection the resin was treated with a solution of Boc-anhydride (50 eq.) and DIPEA (10 eq.) in DMF (0.4 M) for 1 h to protect the N-terminal amino group. The peptides were cleaved from the 2-chlorotrityl resin by treatment with 30 vol.% HFIP in DCM (2 mL/10 µmol peptide) for 2 h, the cleavage solution was collected by filtration and transferred into a round bottom flask. The resin was washed with DCM (2x 1 mL) and the combined filtrates were concentrated in vacuo. The resulting residue was dissolved in anhydrous DMF (300 µl/10 µmol peptide) and cooled to 0 °C. Diphenyldiselenide (30 eq., c = 1 M) in anhydrous DMF was added to the solution followed by *n*Bu_3P (30 eq.). The reaction was allowed to warm to room temperature and proceed for 3 h, after which time the solvent was removed in vacuo. The crude material was cooled to 0 °C and the protecting groups removed via treatment with TFA:TIS:H_2O (95:2.5:2.5 v/v/v; 3 mL/10 µmol peptide). After 1 h the cleavage cocktail was reduced under a stream of Argon. Ice cold Et_2O (~8-10-fold volume) was added, the remaining suspension was centrifuged (4000 rpm, 15 min, 4 °C) and the ether decanted. Afterwards the precipitate was suspended a second time in ether, centrifuged, decanted and the pellet dissolved in H_2O:ACN:TFA (1:1:0.001 v/v/v) and purified by preparative HPLC as indicated.

5.2 Boc-strategy for the Synthesis of Peptide Thioester

Loading of MBHA Resin with MPA-Gly

The resin (~0.67 µmol/mg) was transferred into a syringe equipped with a filter frit and allowed to swell in DCM (10 min). The resin was treated with a solution of DCM:DIPEA (9:1 v/v) and subsequently washed (10x DCM, 5x DMF). Fmoc-Gly-OH (4 eq., c = 0.2 M in DMF) was coupled in presence of PyBOP (4 eq.) and DIPEA (12 eq.). After 1 h the resin was washed (3x DMF, 3x DCM, 3x DMF), acetylated with DMF:Ac_2O:lutidine (89:5:6 v/v/v, 5 min) and washed again (3x DMF, 3x DCM, 3x DMF). The Fmoc-group was removed by treatment of the resin with a solution of 20 % piperidine in DMF (2x 5 min) and the initial loading estimated by the UV-absorbance of the piperidine fulvene adduct (λ = 301 nm, ε = 7800 $M^{-1}cm^{-1}$). Afterwards the resin was washed (3x DMF, 3x DCM, 3x DMF) and *S*-trityl-3-mercaptopropionic acid (4 eq., c = 0.2 M in DMF) was coupled in presence of PyBOP (4 eq.) and DIPEA (12 eq.). After 1 h the resin was washed (3x DMF, 3x DCM, 3x DMF), acetylated with DMF:Ac_2O:lutidine (89:5:6 v/v/v, 5 min) and washed (5 x DMF, 10 x DCM). The trityl group was removed by treatment of the resin with a solution of TFA:TIS (95:5 v/v; 2 x 5 min). Afterwards the resin was washed (10x DCM, 5x DMF) and the first Boc-protected amino acid (4 eq., c = 0.2 M in DMF) coupled in presence of PyBOP (4 eq.) and DIPEA (12 eq.) for 1 h. The subsequent synthesis was done, as described below.

Manual Solid Phase Peptide Synthesis (Boc-strategy) was performed by using the following protocol:

- *Coupling*: Boc-protected amino acids (4 eq.) were activated with HCTU/OxymaPure (4 eq. each), DIPEA (12 eq.) in DMF (final concentration = 0.2 M) and transferred to the resin (coupling time = 20 min).
- *Capping*: The resin was treated with DMF:Ac_2O:lutidine (89:5:6 v/v/v, 5 min) for 3 min.
- *Deprotection*: The resin was treated 5 minutes with TFA:*m*Cresol (95:5 v/v).
- *Final cleavage*: The resin was washed with DCM and dried under vacuum. Then a mixture of TFA:TFMSA:*m*Cresol (16:3:1 v/v/v; 3 mL/10 µmol peptide) was added to the resin. After 2 h the cleavage cocktail was collected by filtration, the resin washed with TFA (3x 0.5 mL) and the combined filtrates were concentrated (~ 1 mL).
- *Peptide precipitation*: To the remaining residue Et_2O (~8-10-fold volume) was added, the suspension was cooled (in dry ice ~30 min) and centrifuged (4000 rpm, 15 min, 4 °C). Afterwards the ether phase was decanted, the remaining peptide was dissolved in H_2O/ACN/TFA (1:1:0.001 v/v/v) and purified by preparative HPLC as indicated.

5.3 Determination of Peptide and Protein Concentration

Spectroscopic determination: Concentrations of peptides containing Tyr (ε_{280} = 1490 M^{-1} cm^{-1}), Phe (ε_{280} = 200 M^{-1} cm^{-1}) or Trp (ε_{280} = 5690 M^{-1} cm^{-1}) residue were determined by dissolving the peptides in a defined volume of H_2O/ACN/TFA (1:1:0.001 v/v/v) and measuring the absorbance (λ = 280 nm) on a *NanoDrop ND-1000* spectrometer considering the molar extinction coefficient of the peptide. In cases of auxiliary-modified peptides, the absorbance of the 2-mercaptopyridine moiety was factored as ε_{280} = 1750 M^{-1} cm^{-1} [3] and the absorbance of the 2-mercapto-(5-methoxypyridine) moiety was determined as ε_{280} = 4860 M^{-1} cm^{-1}. For peptides containing a 2-Hydroxy-5-nitrobenzyl group the molar extinction coefficient ε_{280} = 2400 M^{-1} cm^{-1} was considered.

Gravimetric determination: Concentrations of peptides bearing a *para*-MPyE moiety or no Tyr or Trp residue were determined by weighing of the lyophilized peptide and subsequent addition of a certain volume. Basic amino acid residues (Arg, Lys, His) and free N-terminus were assumed to be present in their corresponding TFA salts and considered for the MW of the weighed peptide.

6. Synthesis of Peptide Chalkoester

6.1 Peptide Thioester

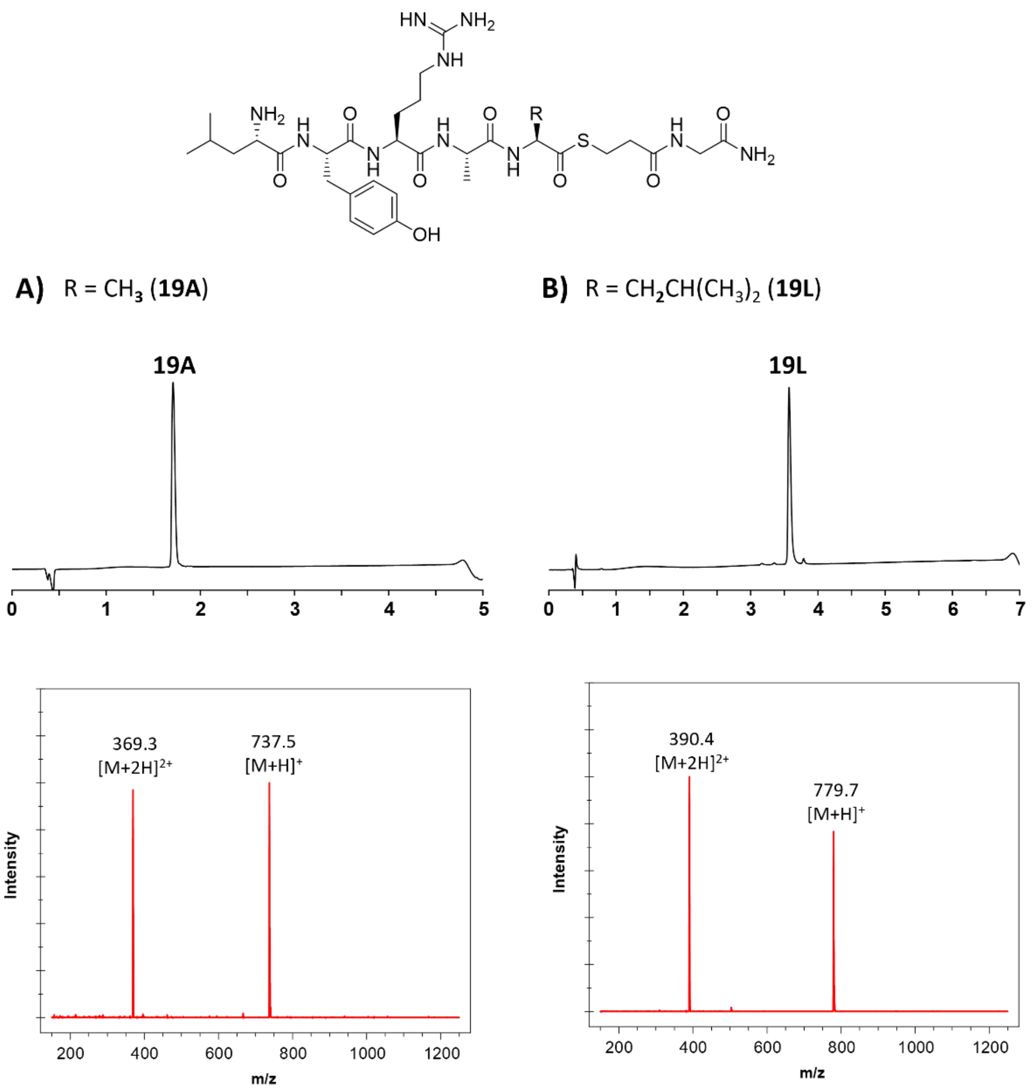

Figure S15: UPLC-trace and ESI-MS spectrum of purified peptide A) Ala-thioester **19A** and B) Leu-thioester **19L**. UPLC analysis: A) 3 - 30 % B in 4 min, λ = 210 nm; B) 3 - 35 % B in 6 min, λ = 210 nm.

6.2 Peptide Selenoester

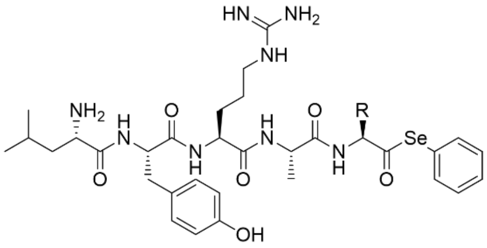

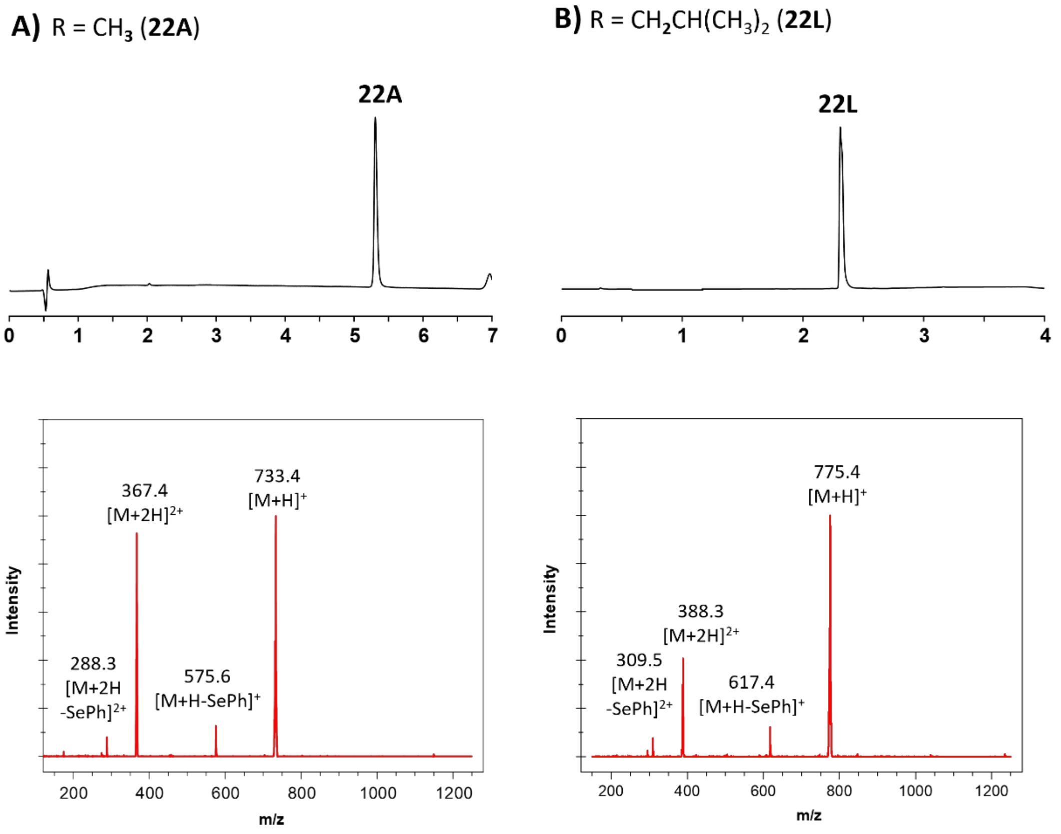

Figure S16: UPLC-trace and ESI-MS spectrum of purified peptide A) Ala-selenoester **22A** and B) Leu-selenoester **22L**. UPLC analysis: 3 - 30 % B in 6 min, λ = 210 nm; 3 - 60 % B in 4 min, λ = 210 nm.

7. Introduction of the Auxiliary

7.1 Introduction of the 2-Mercapto-2-(pyridin-4-yl)ethyl Auxiliary during SPPS

*p*MPyE-NRAEYSGLG (18N)

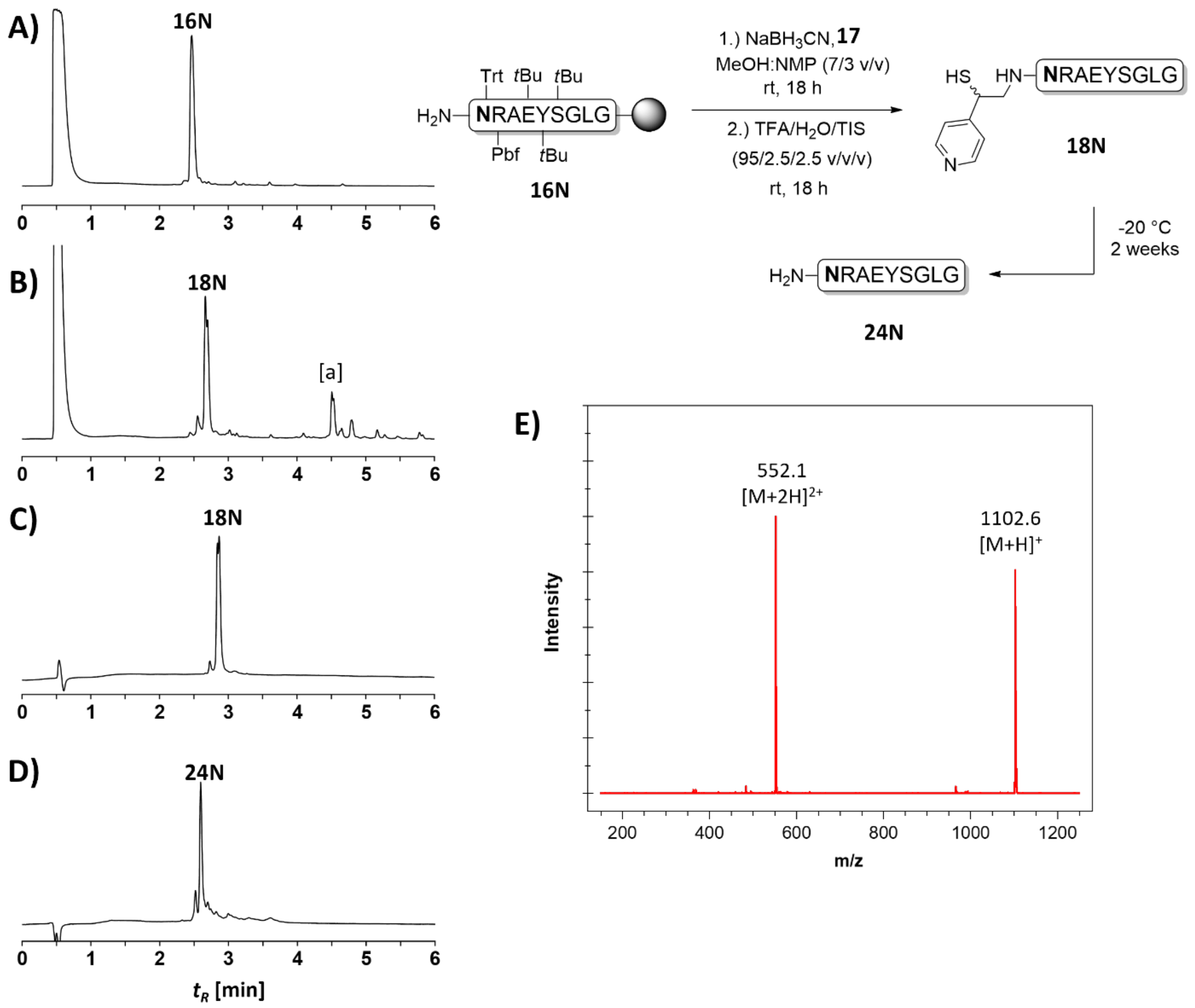

Figure S17: UPLC analysis of crude peptide obtained A) before and B) after reductive alkylation of **18N**. C) UPLC and E) ESI-MS analysis of purified product. D) UPLC trace of after storage of **18N** for 2 weeks at -20 °C as dry lyophilizate. UPLC analysis: 3 - 30 % B in 6 min, λ = 210 nm. [a] Tmb-protected **18N**.

7.2 Introduction of the 2-Mercapto-2-(5-methoxypyridin-2-yl)ethyl Auxiliary during SPPS

MMPyE-NRAEYSGLG (41N)

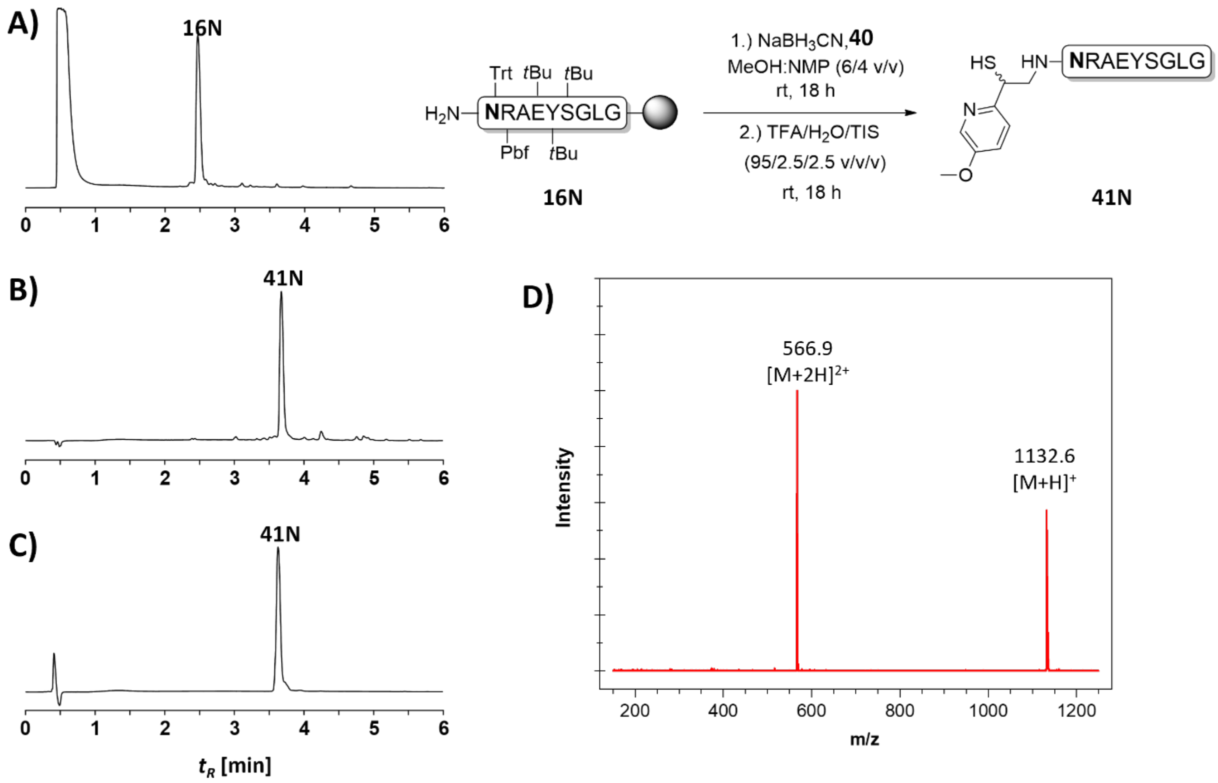

Figure S18: UPLC analysis of crude peptide obtained A) before and B) after reductive alkylation of **16N**. C) UPLC and D) ESI-MS analysis of purified product. UPLC analysis: 3 - 30 % B in 6 min, λ = 210 nm.

MMPyE-VRAEYSGLG (41V)

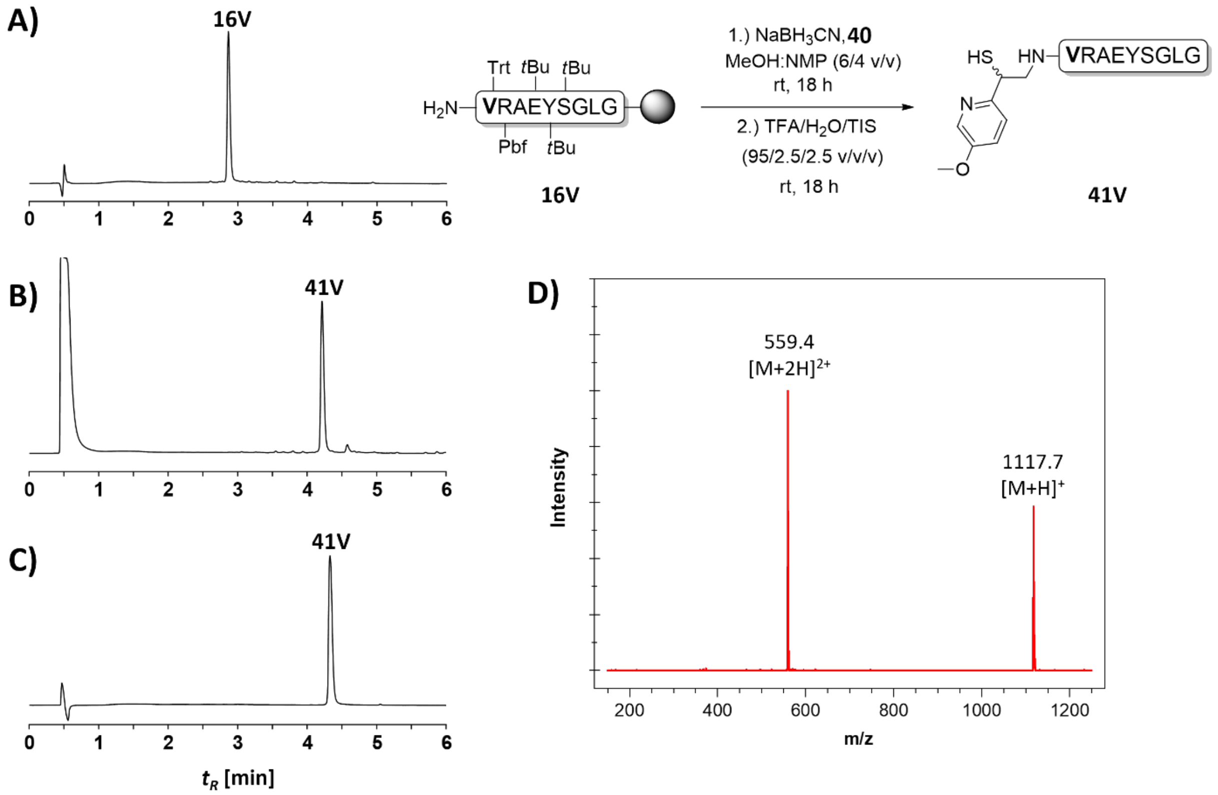

Figure S19: UPLC analysis of crude peptide obtained A) before and B) after reductive alkylation of **16V**. C) UPLC and D) ESI-MS analysis of purified product. UPLC analysis: 3 - 30 % B in 6 min, λ = 210 nm.

7.3 Stability of MPyE- and MMPyE Auxiliary Peptides in Ligation Buffer and Acidic Solution

Peptides with a 2-mercapto-2-(pyridin-2-yl)ethyl moiety decompose when stored in aqueous solution at pH range 2 – 6 at room temperature (Fig 3, Fig S20). For MPyE peptides, auxiliary cleavage can be observed even at pH 6.2 for longer ligation reactions (Fig. S20, A and B). At pH > 7 decomposition is less pronounced, however still noticeable for difficult junctions (Fig. S20, C - E).

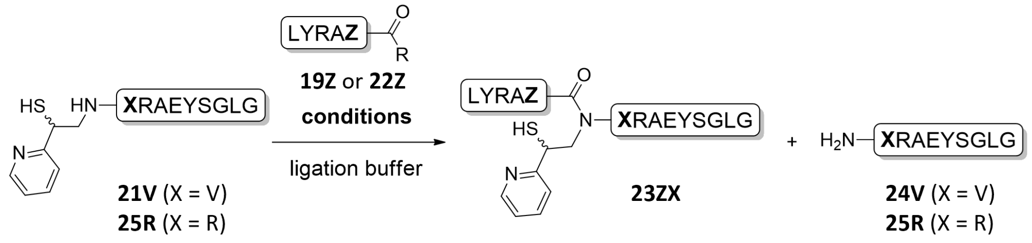

Conditions 1: R = -SePh, DPDS, TCEP, pH 6.2, 24 h

Conditions 2: R = $-(CH_2)_2CO$-Gly-NH_2, PhSH, TCEP, pH 7.5, 24 h

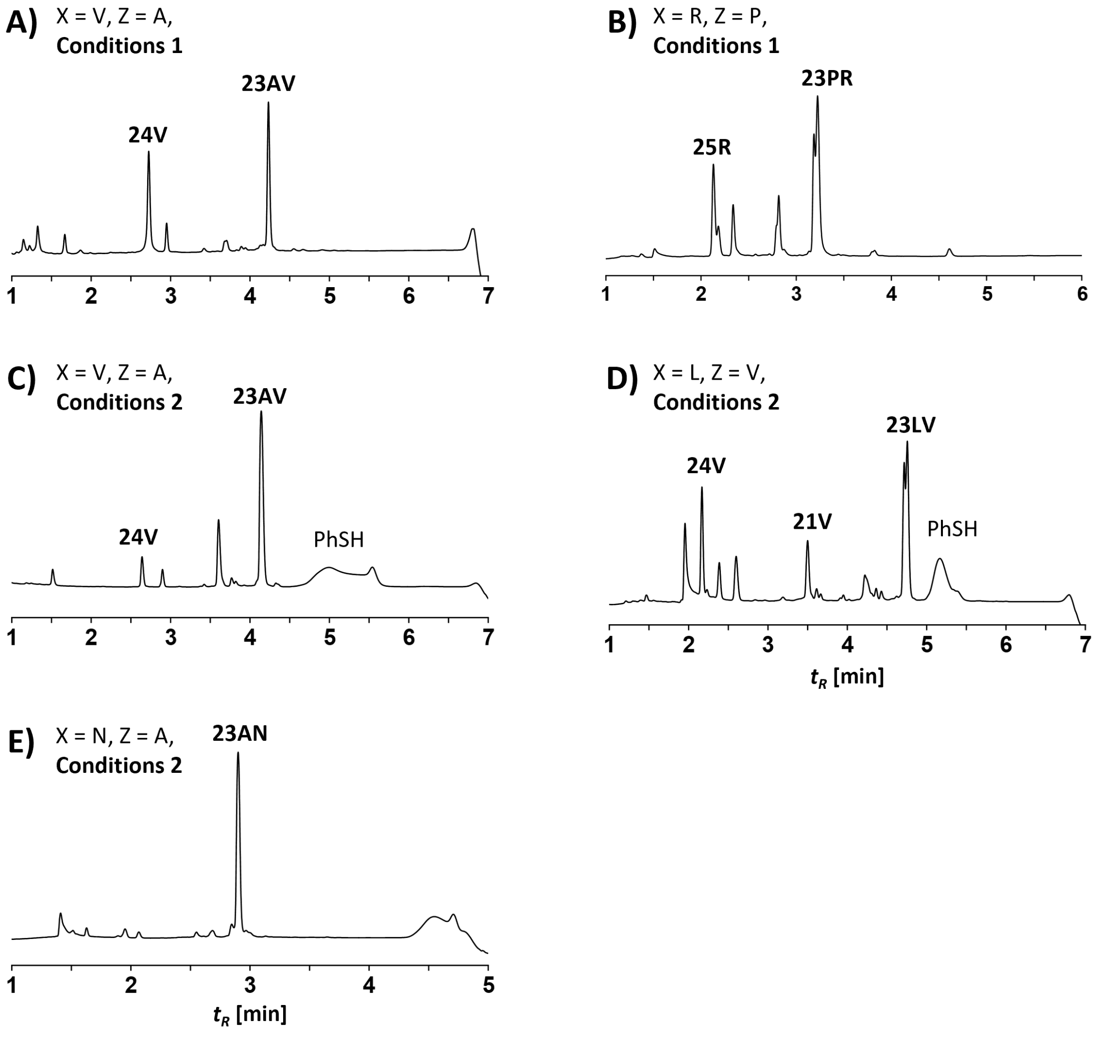

Figure S20: Auxiliary cleavage from MPyE-peptides **21V** and **25R** during ligation using selenoester A) at the A-V junction for 24 h and B) at the P-R junction for 24 h. UPLC trace of the MPyE ligation using thioesters C) at the A-V junction for 24 h, D) at the L-V junction for 24 h and E) at the A-N junction for 24 h.

For the MMPyE-Asn peptide, a putative auxiliary-free peptide **S8N** could not be detected when stored in acidic solution (pH 2) at 4 °C for 48 hours (see figure S21). Only a small fraction of **S8N** was observed when the auxiliary peptide **41N** was incubated in acidic solution at room temperature for 48 h.

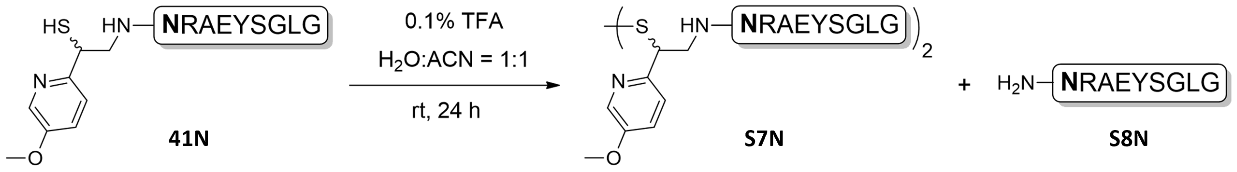

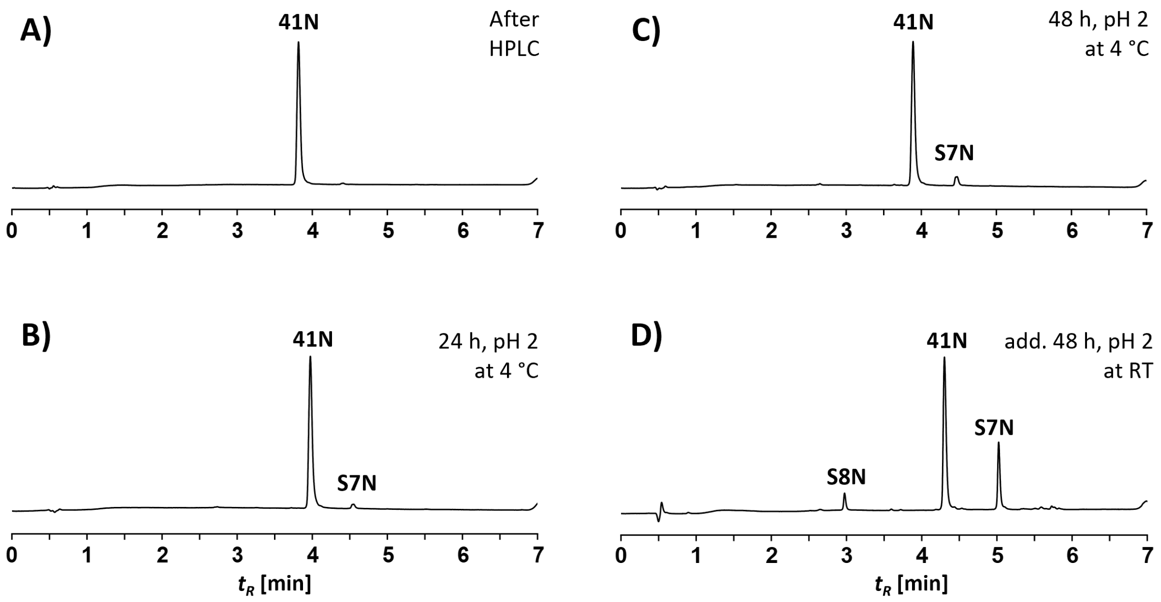

Figure S21: Measurements of the stability of the MMPyE auxiliary on peptide **41N** in acidic aqueous medium (pH ≃ 2) A) after HPLC purification, B) after 24 h at 4 °C, C) after 48 h at 4 °C and D) additional 48 h stored at room temperature. UPLC analysis: 3 - 30 % in 6 min, λ = 210 nm.

Incubation of MPyE-peptide **41V** at pH 2 for 24 h at room temperature resulted in < 2 % cleavage (see figure S22).

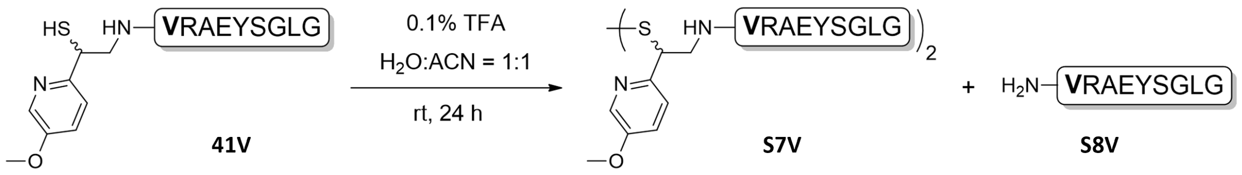

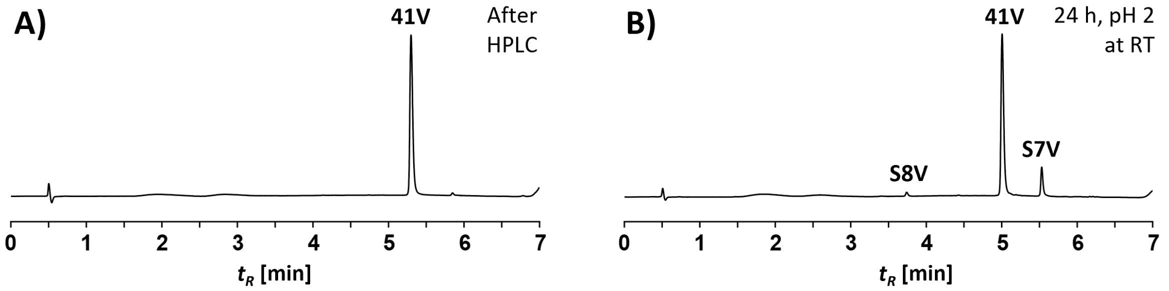

Figure S22: Measurements of the stability of the MMPyE auxiliary on peptide **41V** in acidic aqueous medium (pH ≃ 2) A) after HPLC purification and B) after 24 h at room temperature. UPLC analysis: 3 - 30 % in 6 min, λ = 210 nm.

Additionally, MMPyE-peptide **41V** was incubated in different ligation mixtures specified in Fig. S23 at room temperature to elucidate its stability during ligation.

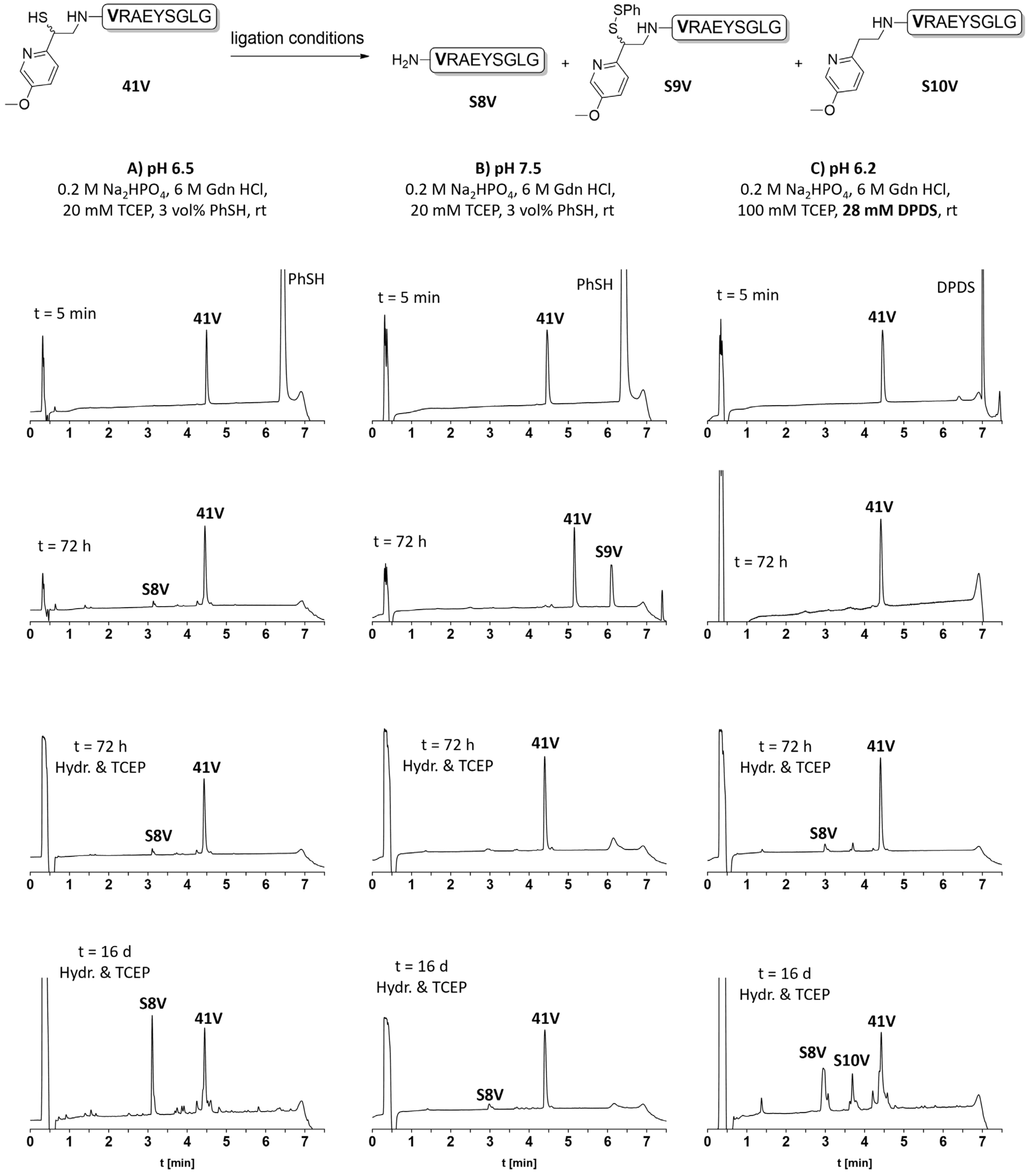

Figure S23: Measurements of the stability of the MMPyE auxiliary on peptide **41V** in different ligation buffer. UPLC analysis: 3 - 30 % in 6 min, λ = 210 nm.

MMPyE-peptide **41Y** was treated with a ligation mixture, optimized for the synthesis of His$_6$-Peg$_6$-(Y*p*SPTSPS)$_6$ (see 11).

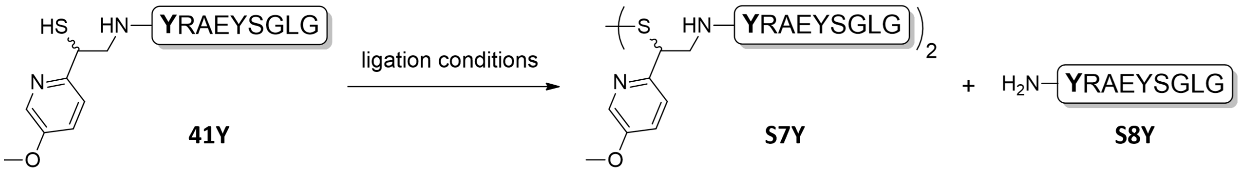

Conditions: 50 mM TCEP, 100 mM MPAA, 0.1 M Na_2HPO_4, 6 M Gdn HCl, pH 6.3, rt, 5 days

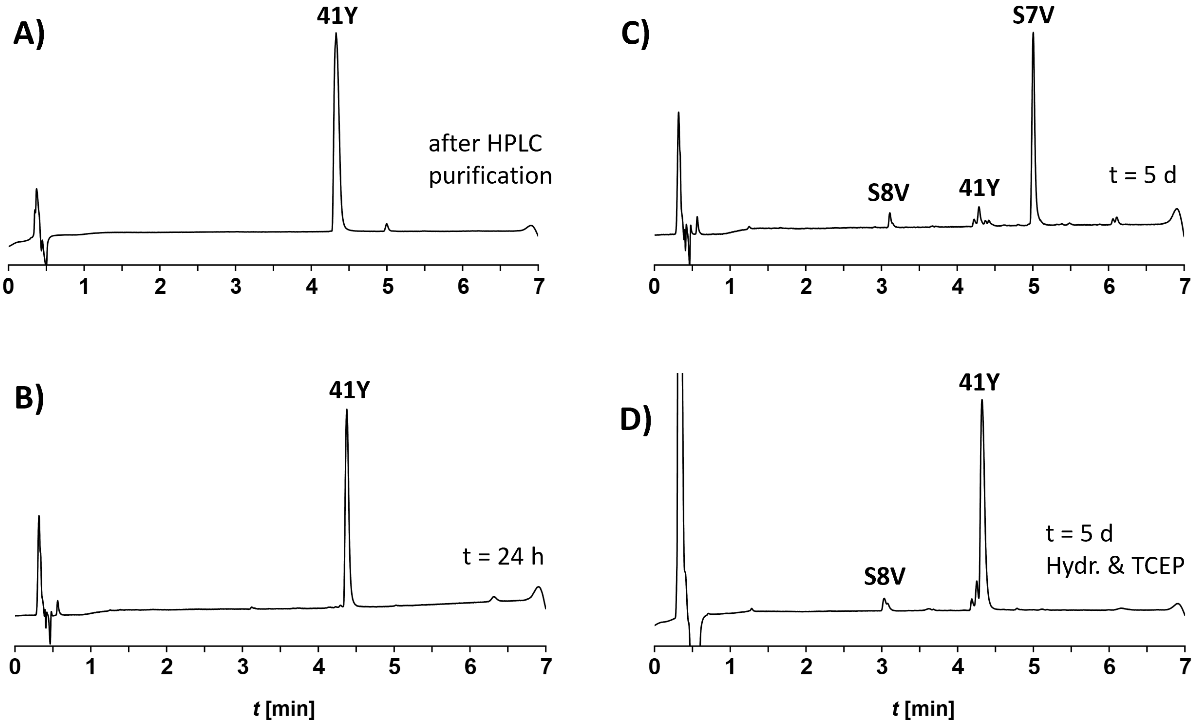

Figure S24: Measurements of the stability of the MMPyE auxiliary on peptide **41Y** in ligation buffer (pH = 6.3). UPLC analysis: 3 - 30 % in 6 min, λ = 210 nm.

8. Native Chemical Ligation

8.1 Ligation of *para*-MPyE- and MMPyE-Peptides with Peptide Thioesters

Ala-Asn Ligation on MMPyE (42AN)

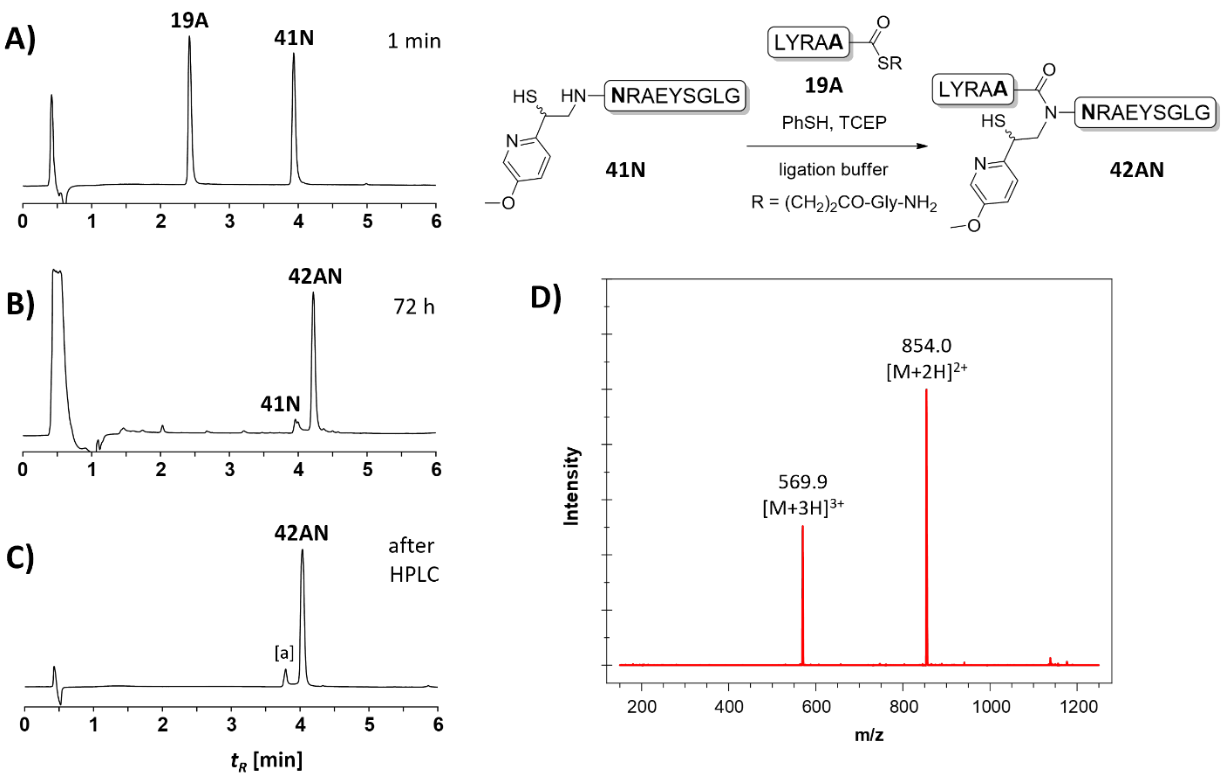

Figure S25: UPLC analysis of aliquots withdrawn at A) t = 1 min and B) t = 72 h (aliquots quenched with 0.1 % TFA, 2.5 % hydrazine and 30 mM TCEP in H_2O). C) UPLC- and D) ESI-MS analysis of purified ligation product **42AN**. Conditions: 3 - 30 % B in 6 min, λ = 210 nm. [a] thioester formed by *N*→*S* rearrangement in acidic medium.

Leu-Asn Ligation on MMPyE (42LN)

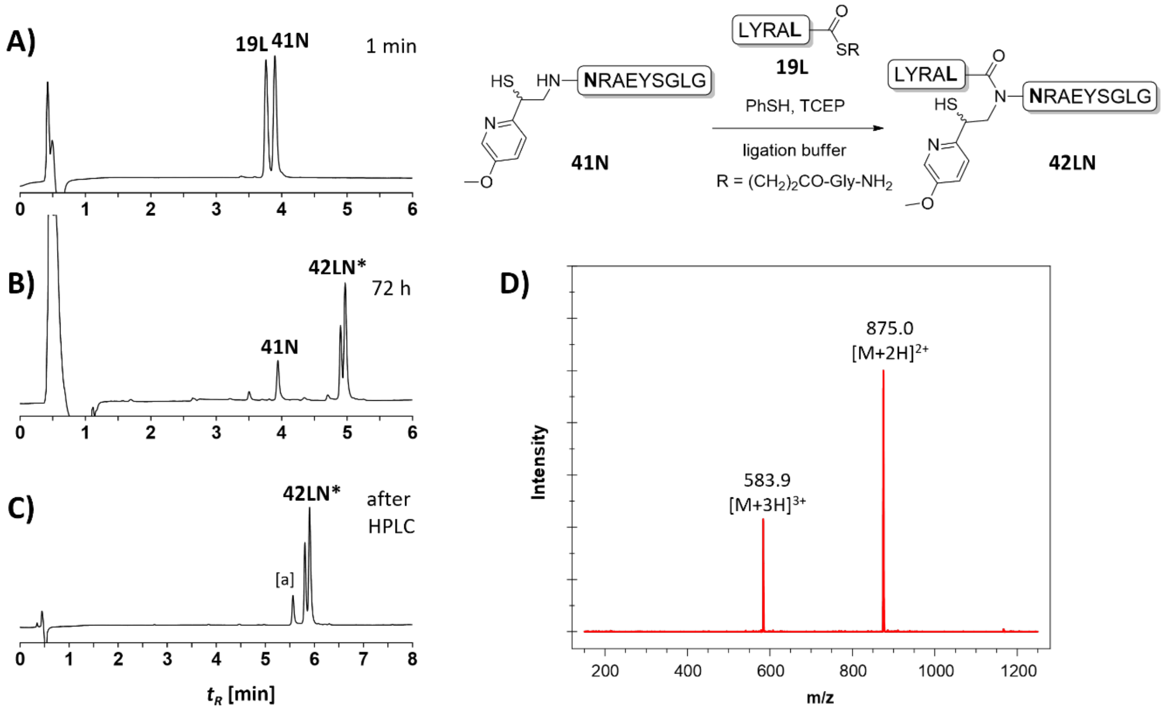

Figure S26: UPLC analysis of aliquots withdrawn at A) t = 1 min and B) t = 72 h (aliquots quenched with 0.1 % TFA, 2.5 % hydrazine and 30 mM TCEP in H_2O). C) UPLC- and D) ESI-MS analysis of purified ligation product **42LN**. Conditions: A), B) 3 - 30 % B in 6 min, λ = 210 nm; C) 3 - 30 % B in 8 min, λ = 210 nm. Pep^N = N-terminal fragment. [a] thioester formed by *N→S* rearrangement in acidic medium. *Mixture of stereoisomers of **42LN**. The fact that isomerism disappears upon auxiliary cleavage (see 9.) is indicative of diastereomers at the auxiliary's stereogenic center.

Ala-Val Ligation on MMPyE (42AV)

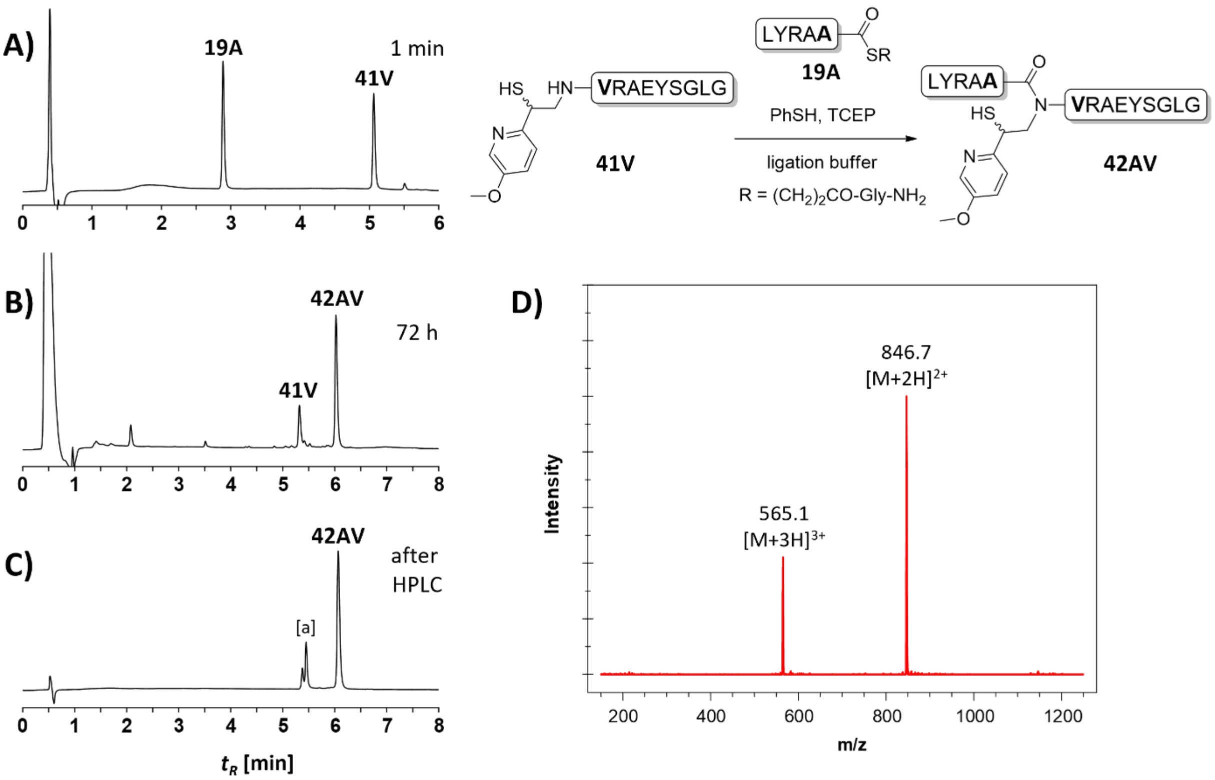

Figure S27: UPLC analysis of aliquots withdrawn at A) t = 1 min and B) t = 72 h (aliquots quenched with 0.1 % TFA, 2.5 % hydrazine and 30 mM TCEP in H_2O). C) UPLC- and D) ESI-MS analysis of purified ligation product **42AV**. Conditions: A) 3 - 30 % B in 6 min, λ = 210 nm; B), C) 3 - 30 % B in 8 min, λ = 210 nm. [a] thioester formed by *N*→*S* rearrangement in acidic medium.

Leu-Val Ligation on MMPyE (42LV)

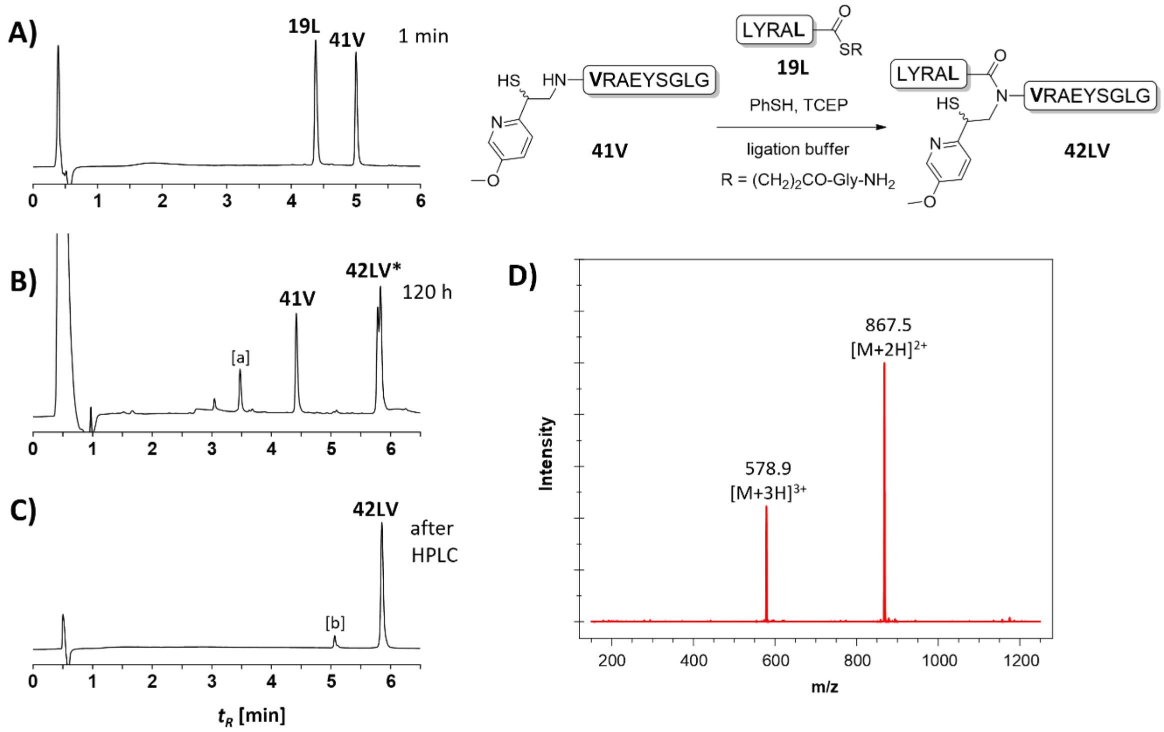

Figure S28: UPLC analysis of aliquots withdrawn at A) t = 1 min and B) t = 120 h (aliquots quenched with 0.1 % TFA, 2.5 % hydrazine and 30 mM TCEP in H_2O). C) UPLC- and D) ESI-MS analysis of purified ligation product **42LV**. Conditions: A) 3 - 30 % B in 6 min, λ = 210 nm; B), C) 3 - 35 % B in 6 min, λ = 210 nm. [a] hydrolysed **19L**. [b] thioester formed by *N→S* rearrangement in acidic medium. *in some cases, separation of the stereoisomers was visible. The fact that isomerism disappears upon auxiliary cleavage (see 9.) is indicative of diastereomers at the auxiliary's stereogenic center.

8.2 Ligation of MMPyE-Peptides with Peptide Selenoesters

Ala-Asn Ligation on MMPyE (→ 42AN) with Selenoester 22A

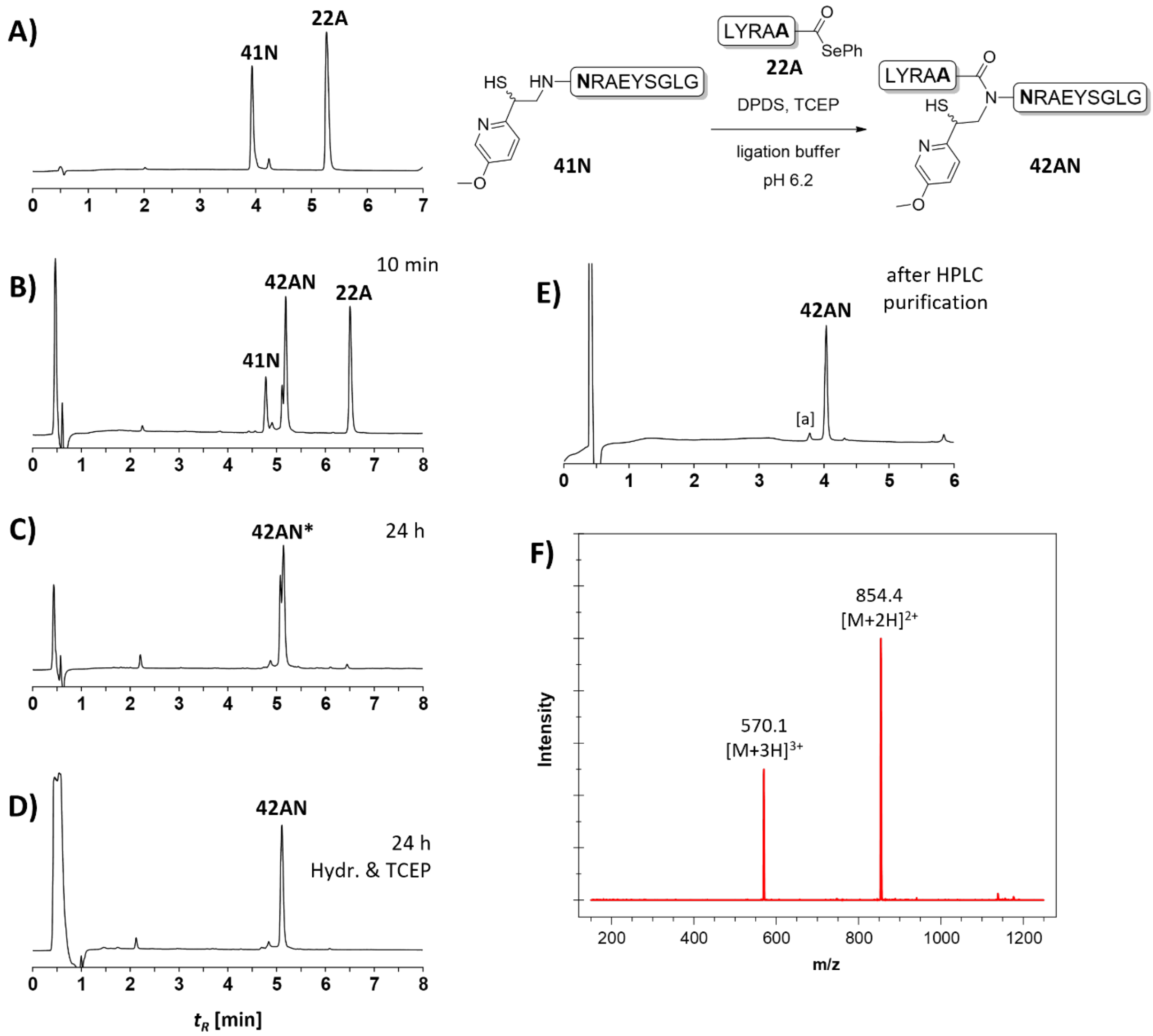

Figure S29: UPLC analysis of A) a mixture of **41N** and **22A** and B), C), D) aliquots withdrawn from reactions after B) t = 10 min, C) t = 24 h and D) t = 24 h with hydrazine (2.5 %) and TCEP (30 mM) added. F) ESI-MS analysis of purified ligation product **42AN**. Conditions: A) 3 - 30 % B in 6 min, λ = 210 nm; B) - D) 3 - 30 % B in 8 min, λ = 210 nm.

Leu-Val Ligation on MMPyE (→ 42LV) with Selenoester 22A

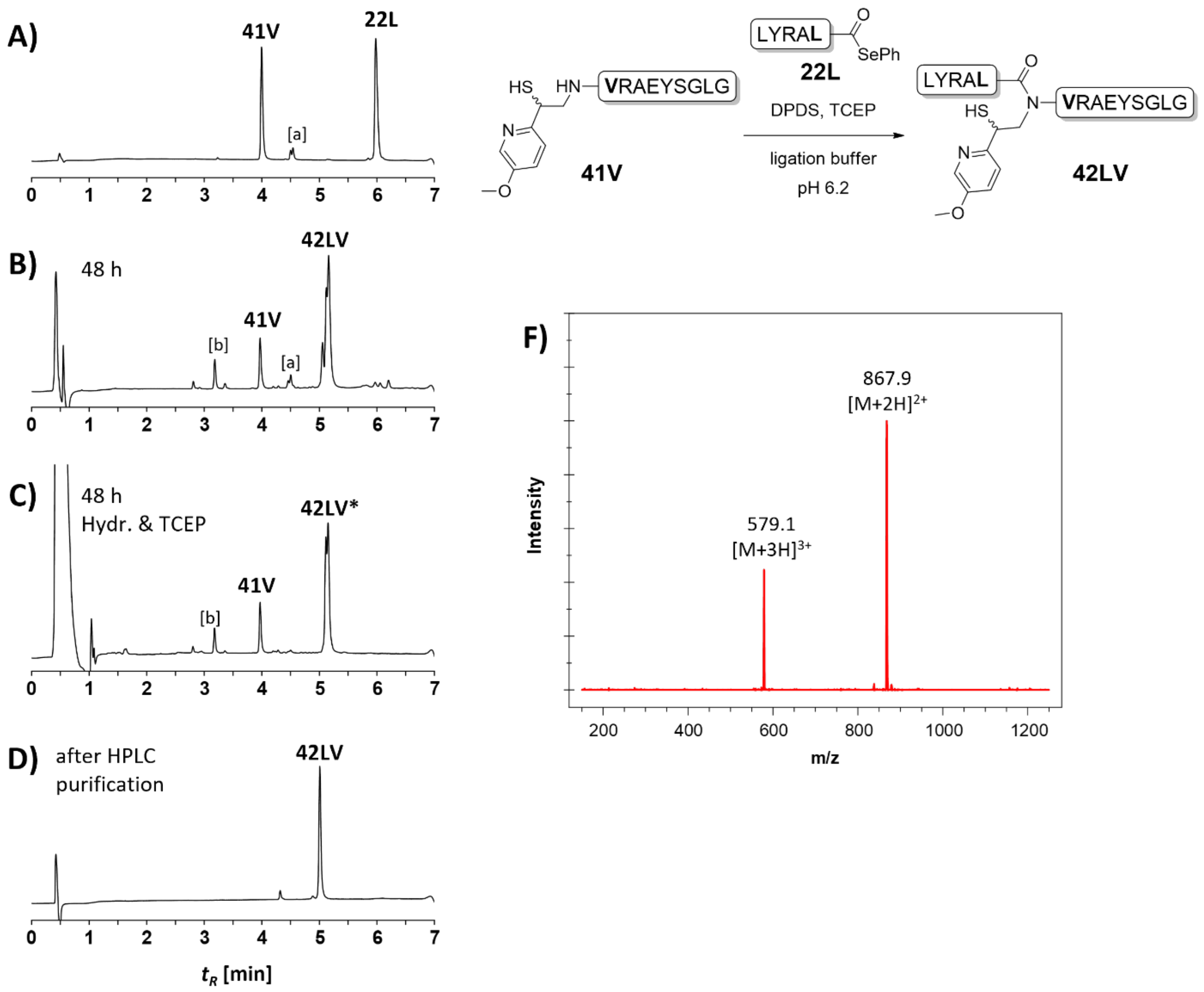

Figure S30: UPLC analysis of A) a mixture of **41V** and **22L** and B), C), D) aliquots withdrawn from reactions after B) t = 48 h and C) t = 48 h reaction with hydrazine (2.5 %) and TCEP (30 mM) added. F) ESI-MS analysis of purified ligation product **42AV**. Conditions: 3 - 35 % B in 6 min, λ = 210 nm. [a] **42LV** before *S*→*N* rearrangement. [b] hydrolysed **22L**.

8.3 Ligation of MMPyE-Peptides with Peptide Thioesters at Elevated Temperatures

MMPyE peptides were allowed to react with thioesters at elevated temperatures following the general procedure for Introduction of MMPyE Auxiliary (see Experimental Section) with the temperature specified in figure S31.

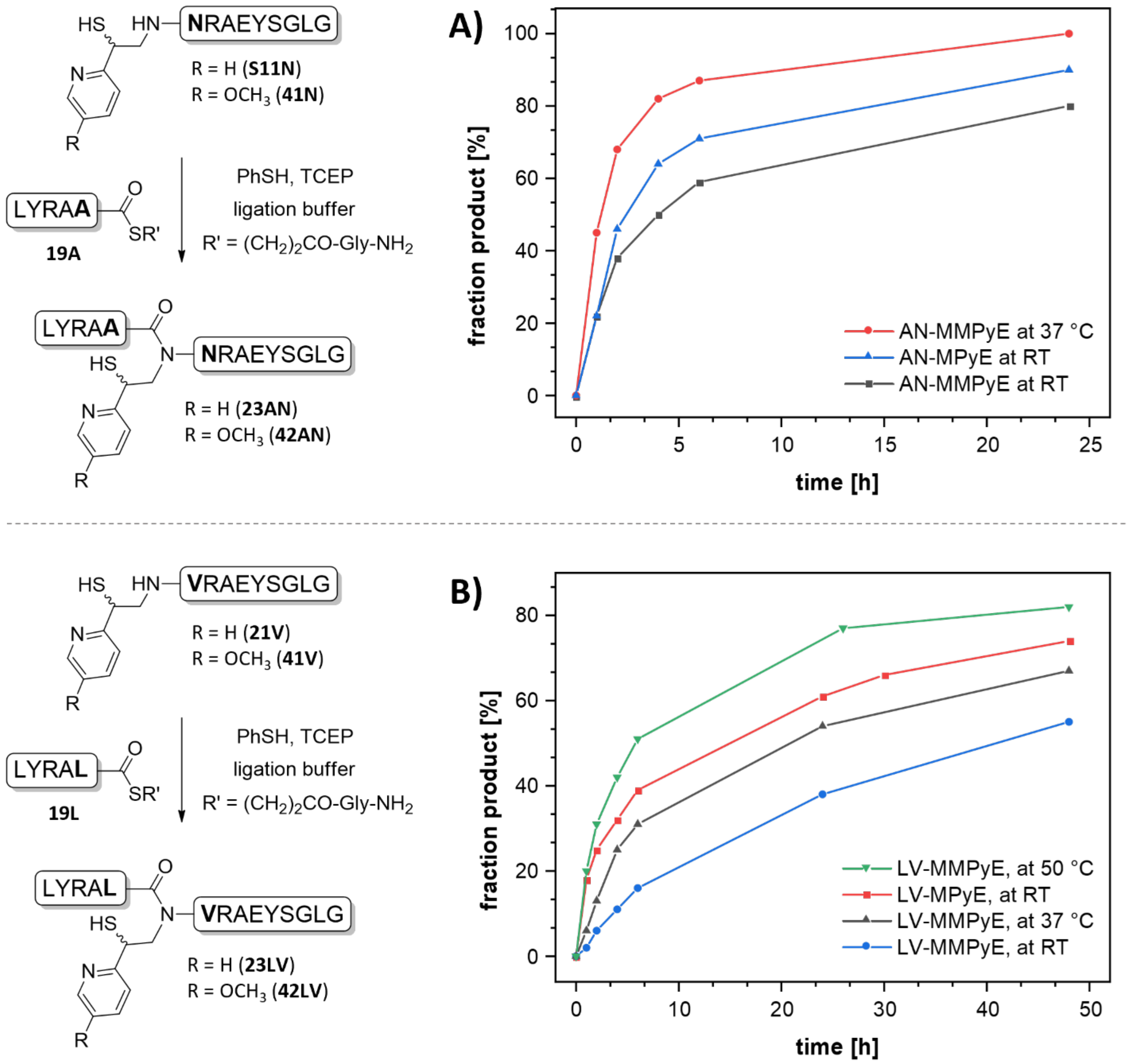

Figure S31: A) Time course of MMPyE ligation at the AN junction at RT and 37 °C compared to the MPyE ligation at room temperature. B) Time course of MMPyE ligation at the LV junction at RT, 37 °C and 50 °C compared to the MPyE ligation at room temperature.

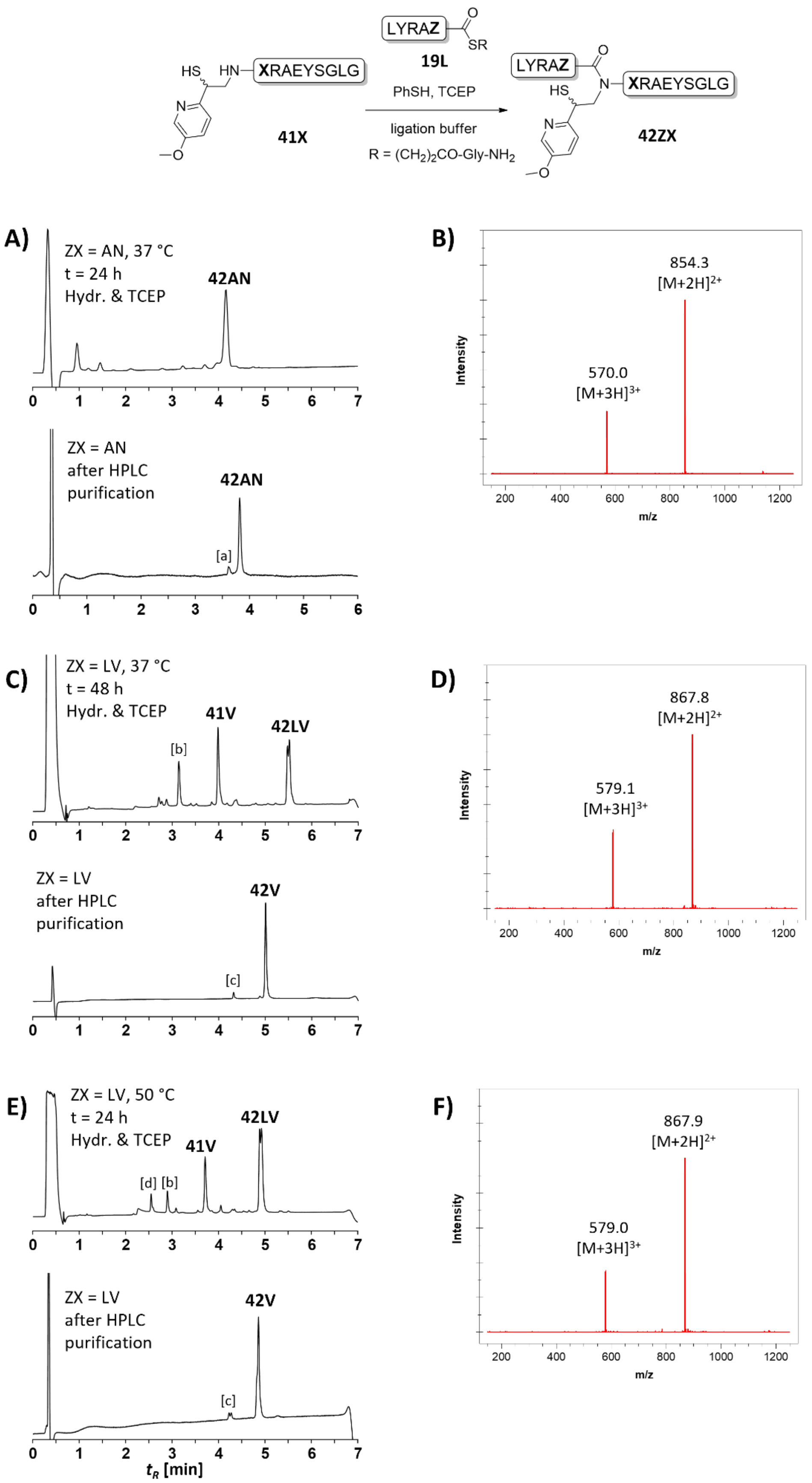

Figure S32: Ligation of peptides **41X** and **19Z** at elevated temperatures. UPLC analysis of aliquots withdrawn after quenching with hydrazine (2.5 %) and TCEP (30 mM) and after HPLC purification for A) AN ligation at 37 °C for 24 h, C) LV ligation at 37 °C for 48 h and E) LV ligation at 50 °C for 24 h. ESI-MS analysis of purified ligation product B) **42AN** from 37 °C reaction, D) **42LV** from 37 °C reaction and F) **42AN** from 50 °C reaction. Thioester formed by *N*→*S* rearrangement in acidic medium for [a] **42AN** and [c] **42LV**. [b] hydrolysed **19L**. [d] **41V** with cleaved auxiliary.

Ala-Asn Ligation on MMPyE (→ 37AN) at 37 °C

500 nmol of MMPyE-peptide **36N** and 750 nmol of peptide thioester **19A** were dissolved in 100 µL ligation buffer (see 8.1) and allowed to shake at 37 °C under argon atmosphere for 24 h. The reaction was quenched by addition of 5 µL of 51 % aqueous hydrazine solution and subsequently 10 µL of 1 M TCEP solution. The crude ligation product was purified by preparative HPLC using a linear gradient from 3-30 % B in 40 min. The desired ligation product **37AN** was isolated as a white solid after lyophilization, which was dissolved in H_2O/ACN/TFA (1:1:0.001 v/v/v) for a spectroscopic determination of the synthesis yield (A_{280} = 0.52, V = 0.5 mL, 330 nmol, 66 %). Retention times and mass spectrometry data were consistent with the obtained compound of thioester ligation (see 8.1).

Leu-Val Ligation on MMPyE (→ 37LV) at 37 °C

410 nmol of MMPyE-peptide **36V** and 615 nmol of peptide thioester **19L** were dissolved in 80 µL ligation buffer (see 8.1) and allowed to shake at 37 °C under argon atmosphere for 48 h. The reaction was quenched by addition of 5 µL of 51 % aqueous hydrazine solution and subsequently 10 µL of 1 M TCEP solution. The crude ligation product was purified by preparative HPLC using a linear gradient from 3-30 % B in 40 min. The desired ligation product **37LV** was isolated as a white solid after lyophilization, which was dissolved in H_2O/ACN/TFA (1:1:0.001 v/v/v) for a spectroscopic determination of the synthesis yield (A_{280} = 0.36, V = 0.3 mL, 139 nmol, 34 %). Retention times and mass spectrometry data were consistent with the obtained compound of thioester ligation (see 8.1).

Leu-Val Ligation on MMPyE (→ 37LV) at 50 °C

250 nmol of MMPyE-peptide **36V** and 375 nmol of peptide thioester **19L** were dissolved in 50 µL ligation buffer (see 8.1) and allowed to shake at 37 °C under argon atmosphere for 24 h. The reaction was quenched by addition of 5 µL of 51 % aqueous hydrazine solution and subsequently 10 µL of 1 M TCEP solution. The crude ligation product was purified by preparative HPLC using a linear gradient from 3-30 % B in 40 min. The desired ligation product **37LV** was isolated as a white solid after lyophilization, which was dissolved in H_2O/ACN/TFA (1:1:0.001 v/v/v) for a spectroscopic determination of the synthesis yield (A_{280} = 0.272, V = 0.3 mL, 104 nmol, 41 %). Retention times and mass spectrometry data were consistent with the obtained compound of thioester ligation (see 8.1).

9. Auxiliary Removal

9.1 Removal of the MMPyE Auxiliary

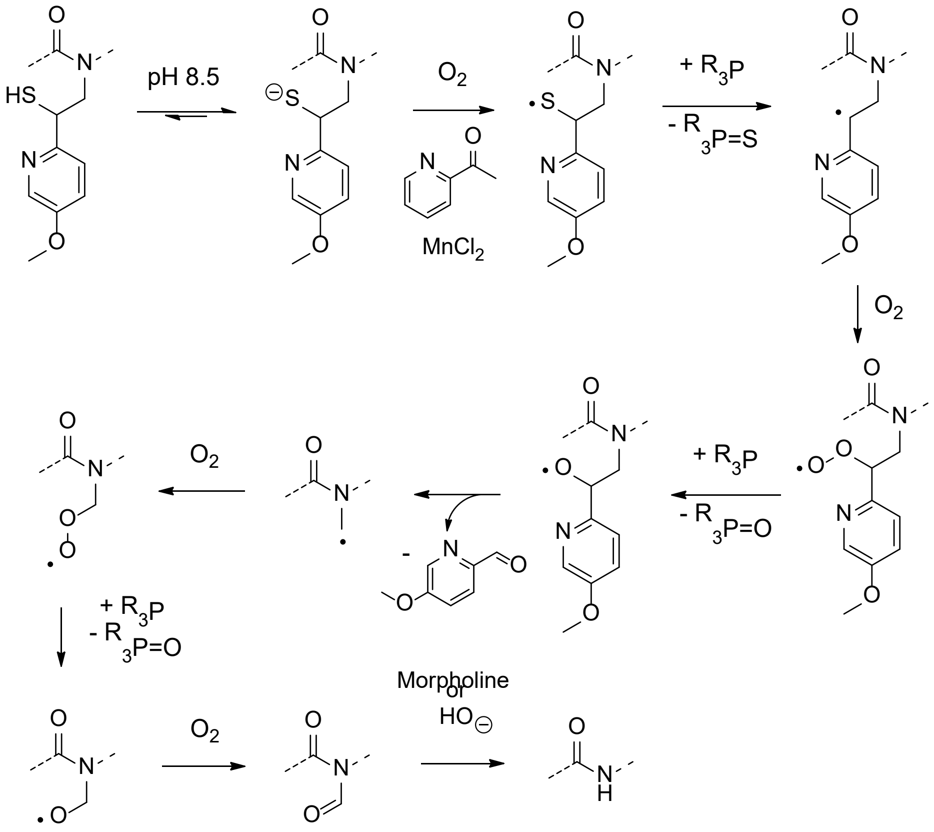

Figure S33: Putative reaction mechanism for the cleavage of the MMPyE auxiliary from a ligated peptide.

Removal of Auxiliary from Ligation Product AN (51AN)

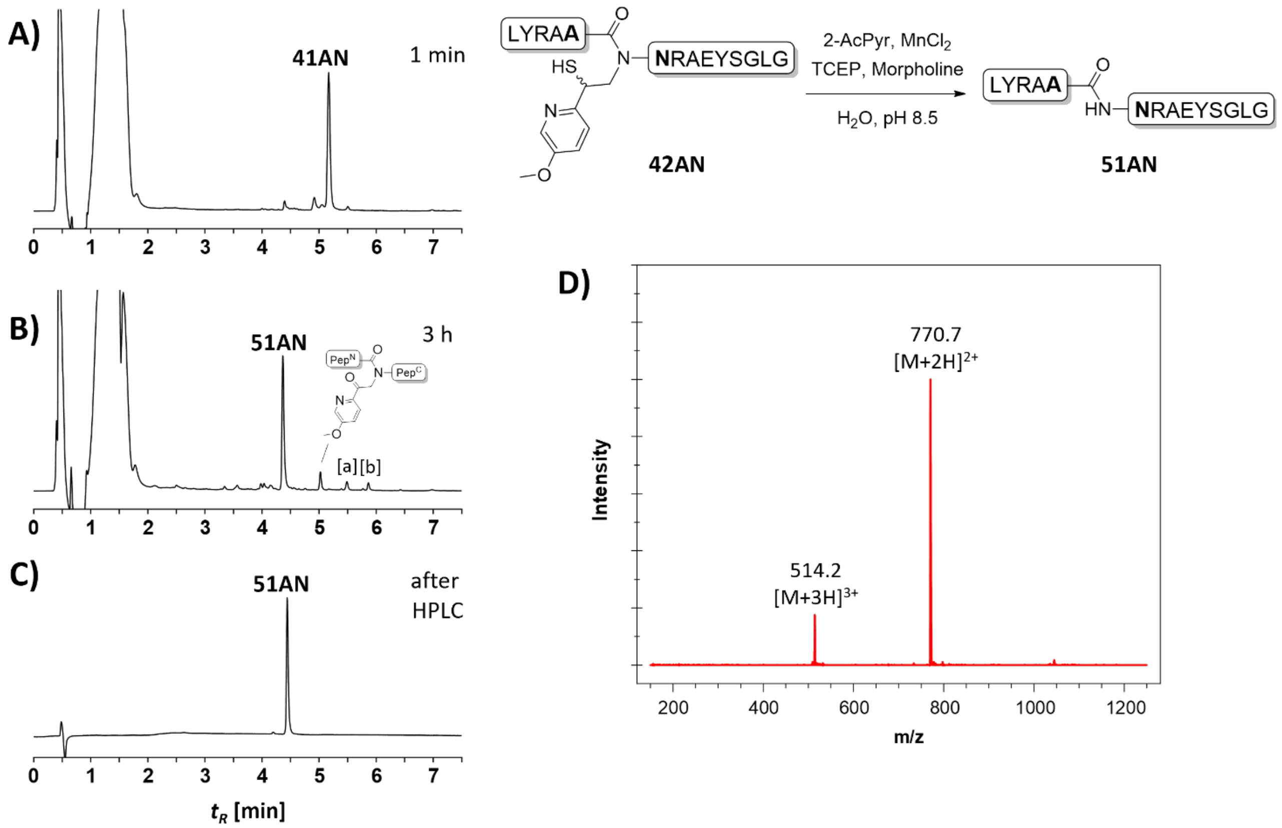

Figure S34: Auxiliary cleavage from **42AN**. UPLC analysis of aliquots withdrawn after A) 1 min, B) 3 h reaction time and C) of purified peptide **51AN**. D) ESI-MS of purified **51AN**. Conditions: 3 % B for 1 min, then 3 - 30 % B in 6 min, λ = 210 nm. [a] methylated **51AN**; [b] formylated **51AN**.

Removal of Auxiliary from Ligation Product AV (51AV)

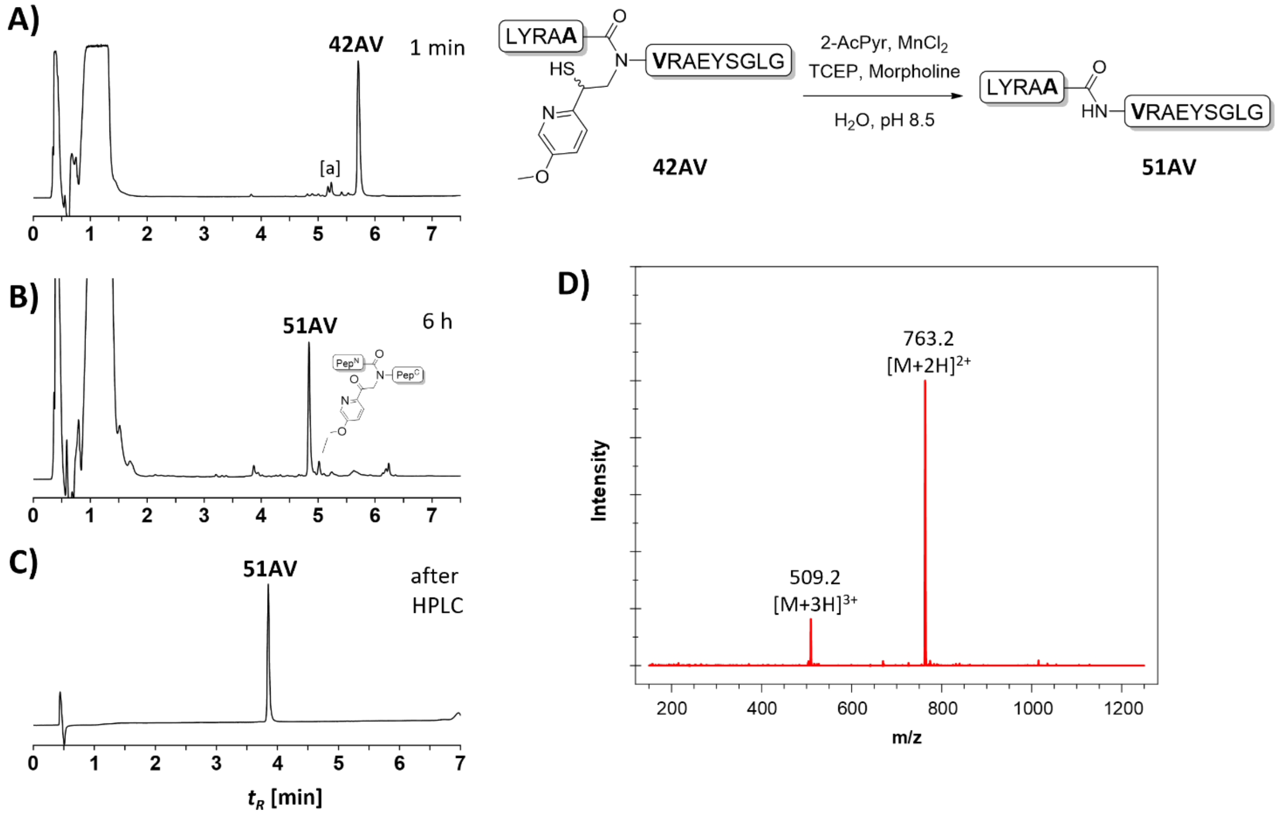

Figure S35: Auxiliary cleavage from **42AV**. UPLC analysis of aliquots withdrawn after A) 1 min , B) 6 h reaction and C) of purified peptide **51AV**. D) ESI-MS of purified **51AV**. Conditions: A), B) 3 % B for 1 min, then 3 - 30 % B in 6 min, λ = 210 nm; C) 3-30 % B in 6 min, λ = 210 nm. [a] thioester formed by *N*→*S* rearrangement.

Removal of Auxiliary from Ligation Product LN (51LN)

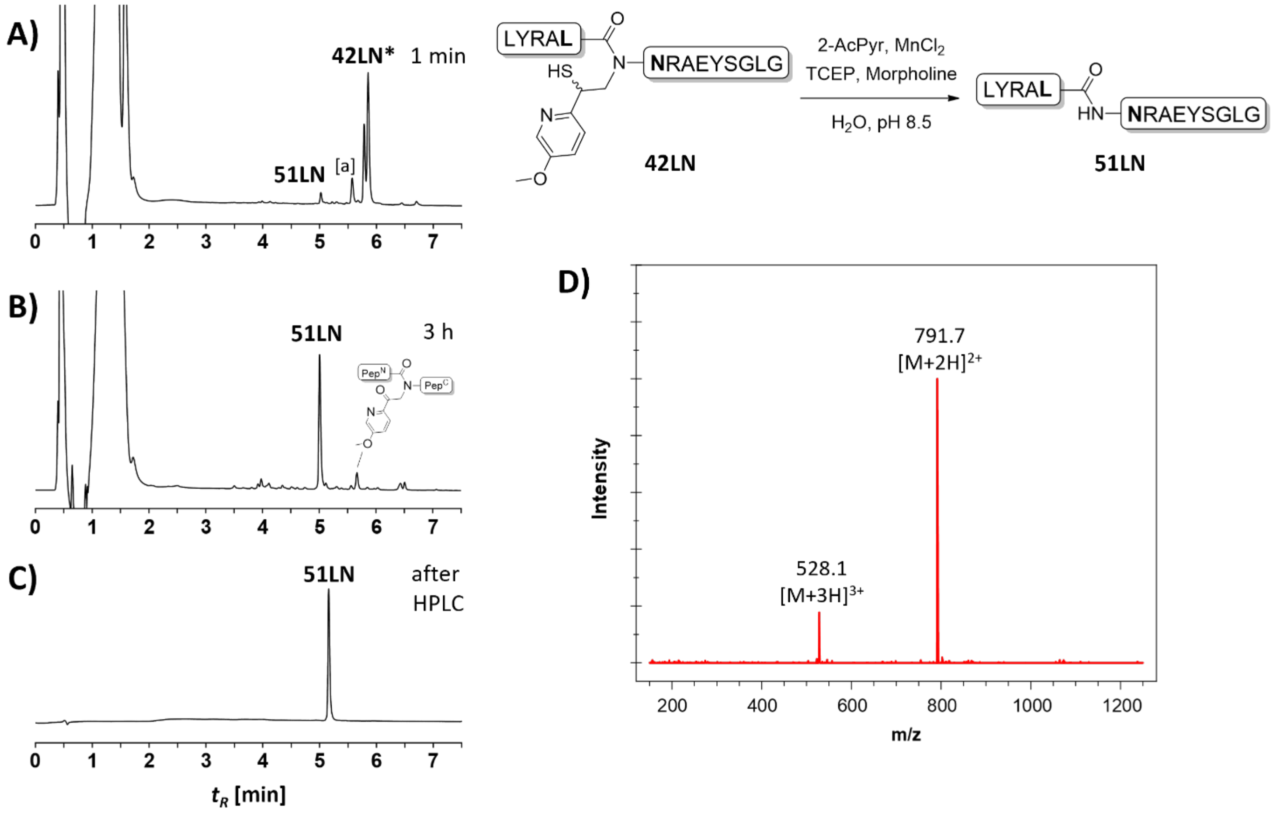

Figure S36: Auxiliary cleavage from **42LN**. UPLC analysis of aliquots withdrawn after A) 1 min, B) 3 h reaction and C) of purified peptide **42LN**. D) ESI-MS of purified **51LN**. Conditions: 3 % B for 1 min, then 3 - 30 % B in 6 min, λ = 210 nm. [a] thioester formed by *N→S* rearrangement.

Removal of Auxiliary from Ligation Product LV (51LV)

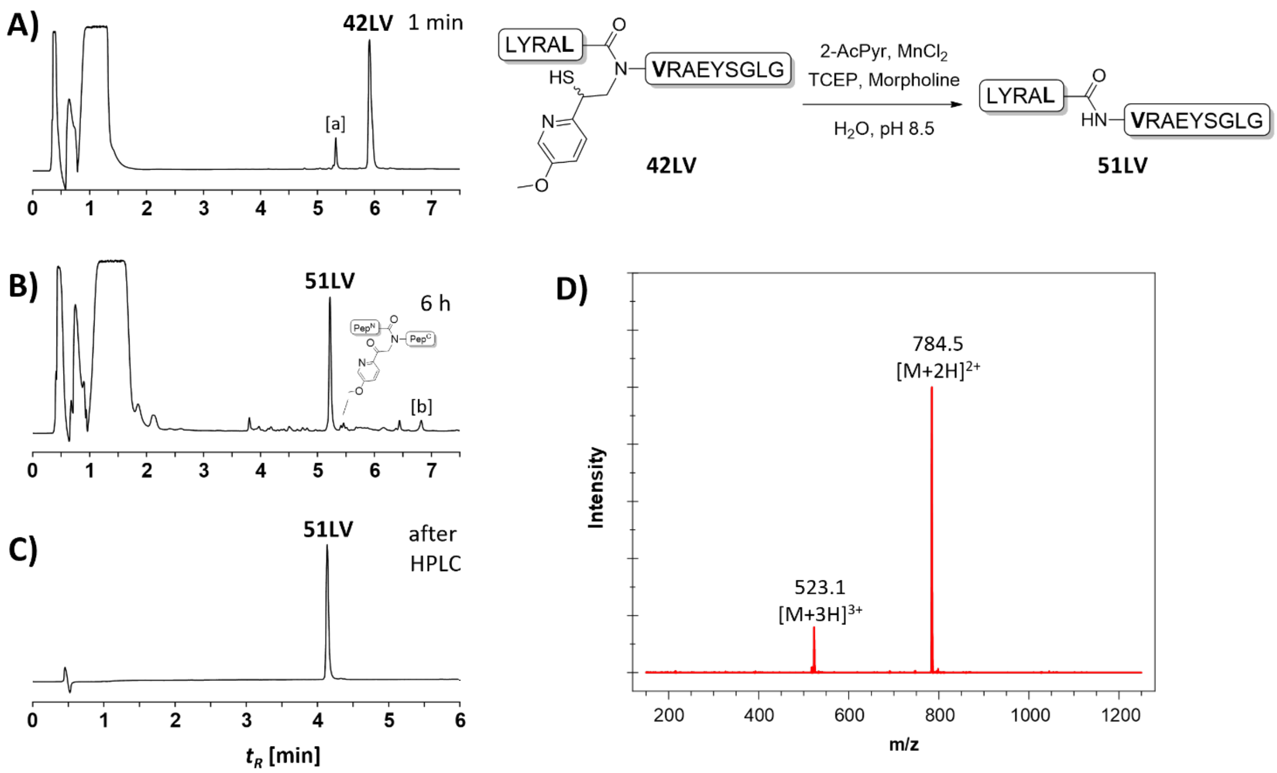

Figure S37: Auxiliary removal from **42LV**. UPLC analysis of aliquots withdraw n from cleavage reactions after A) 1 min, B) 6 h and C) of purified peptide **51LV**. D) ESI-MS of purified **51LV**. Conditions: A), B) 3 % B for 1 min, then 3 - 30 % B in 6 min, λ = 210 nm; C) 3-30 % B in 6 min, λ = 210 nm. [a] thioester formed by *N*→*S* rearrangement; [b] methylated **51LV**.

9.2 Comparison of Auxiliary Removal of MPyE- and MMPyE-Asn Peptide

For a side-by-side comparison of removal rates, ligated MPyE peptide **23AN** (50 nmol) and MMPyE peptide **42AN** (50 nmol) were lyophilized from stock solution and dissolved in 100 µL auxiliary cleavage mixture (see 9.1). Aliquots were withdrawn quenched with Ascorbic acid (0.1 M in H_2O) and analyzed by UPLC-MS to monitor the progress of the reaction. The progress of the ligation reaction was assessed by integration of the corresponding peak areas (Fig. 7).

Product Formed by Removal Mixture

Incubation of the removal cocktail for t > 6 h can lead to the accumulation of a non-peptidic product with m/z of 475. This compound was also observed with the MPyE auxiliary and has not been observed to be detrimental for removal reaction.[3] Separation from polar peptides can be accomplished by prolonged rinsing during HPLC purification, extraction, size-exclusion chromatography or other purification methods.

10. Synthesis of P1-Muc5AC$_8$-P4

EIQALEEENAQLEQENAALEEEIAQLEY-(APTTSTTS)$_4$ Thioester (P1-Muc5AC$_4$, 53)

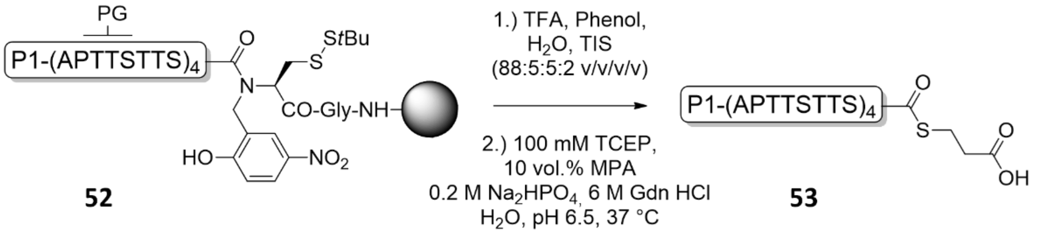

52 **53**

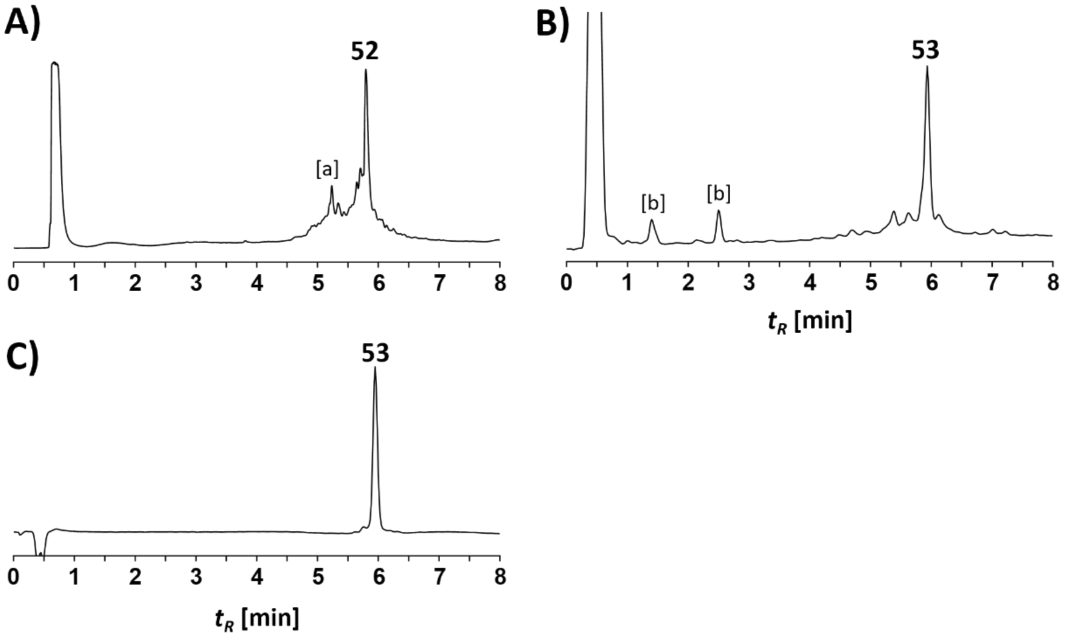

Figure S38: UPLC trace of A) crude **52** after a test cleavage, B) crude **53** after reaction with TCEP (100 mM) and MPA (10 vol.%) and C) purified **52**. Conditions: 15 - 35 % B in 8 min, λ = 210 nm. [a] truncations from SPPS. [b] nonpeptidic material.

$(APTTSTTS)_4$-KIAQLKQKIQALKQENQQLEEENAALEY Auxiliary Peptide (Muc5AC$_4$-P4, 54)

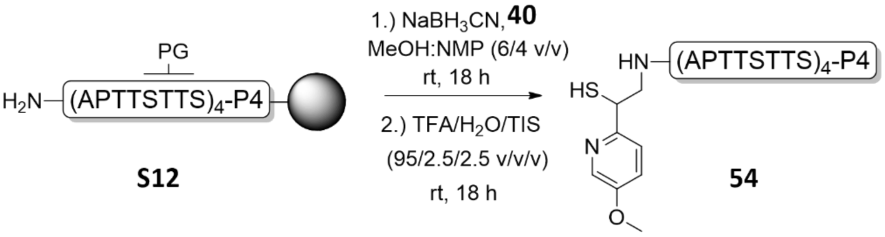

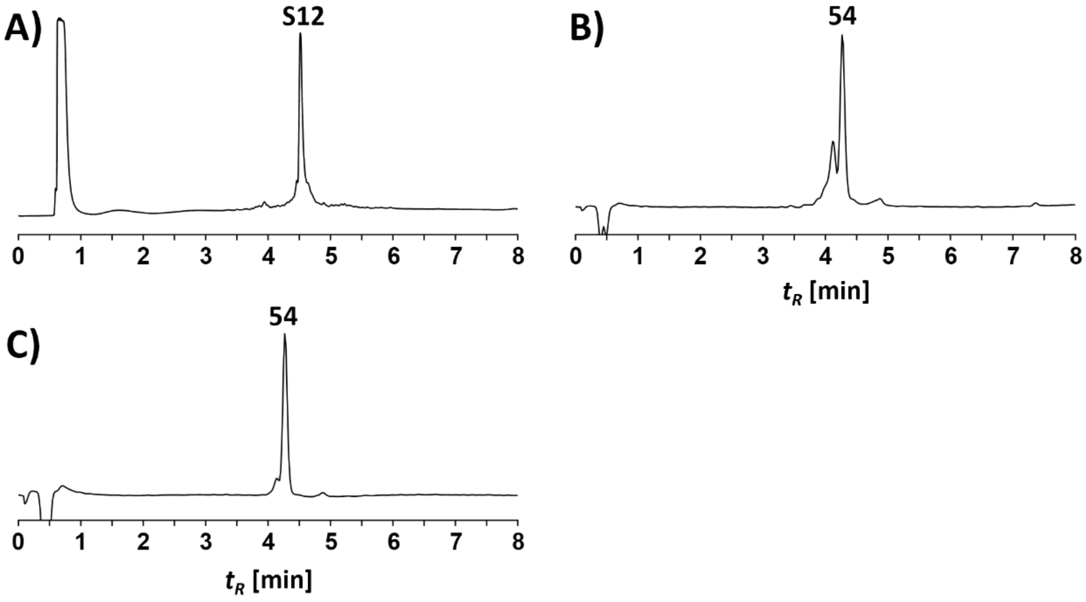

Figure S39: UPLC analysis of crude **S12** A) before and B) after reductive alkylation. C) Purified MMPyE-peptide **54** analyzed by UPLC. Conditions: 15 - 35 % B in 8 min, λ = 210 nm.

Ligation of 54 and 53 (55)

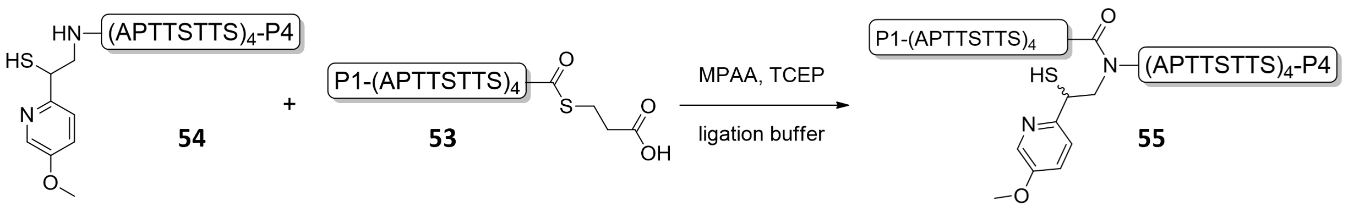

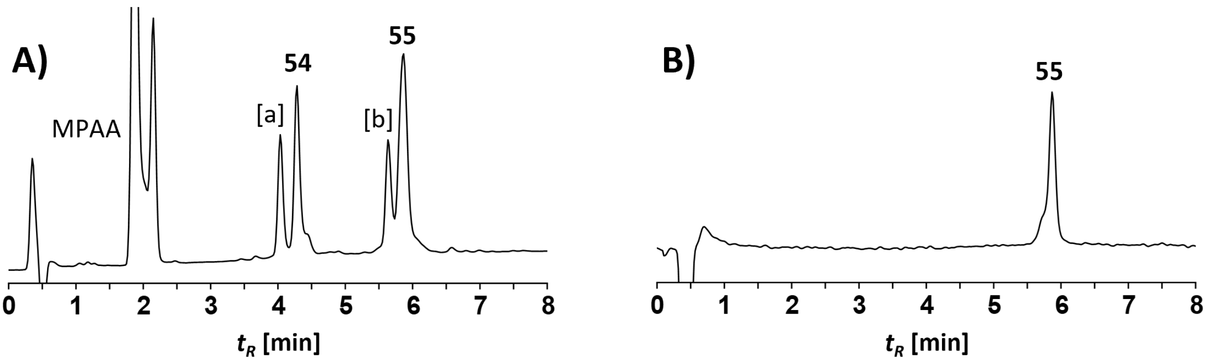

Figure S40: A) UPLC trace of ligation of peptide **53** and **54** at t = 5 d and treatment with hydrazine (2.5 vol.%). B) Purified ligation product **55** analyzed by UPLC-trace. Conditions: 15 - 35 % B in 8 min, λ = 210 nm. [a] **54** with cleaved auxiliary. [b] peptide hydrazide of **53**.

P1-Muc5AC$_8$-P4 (51)

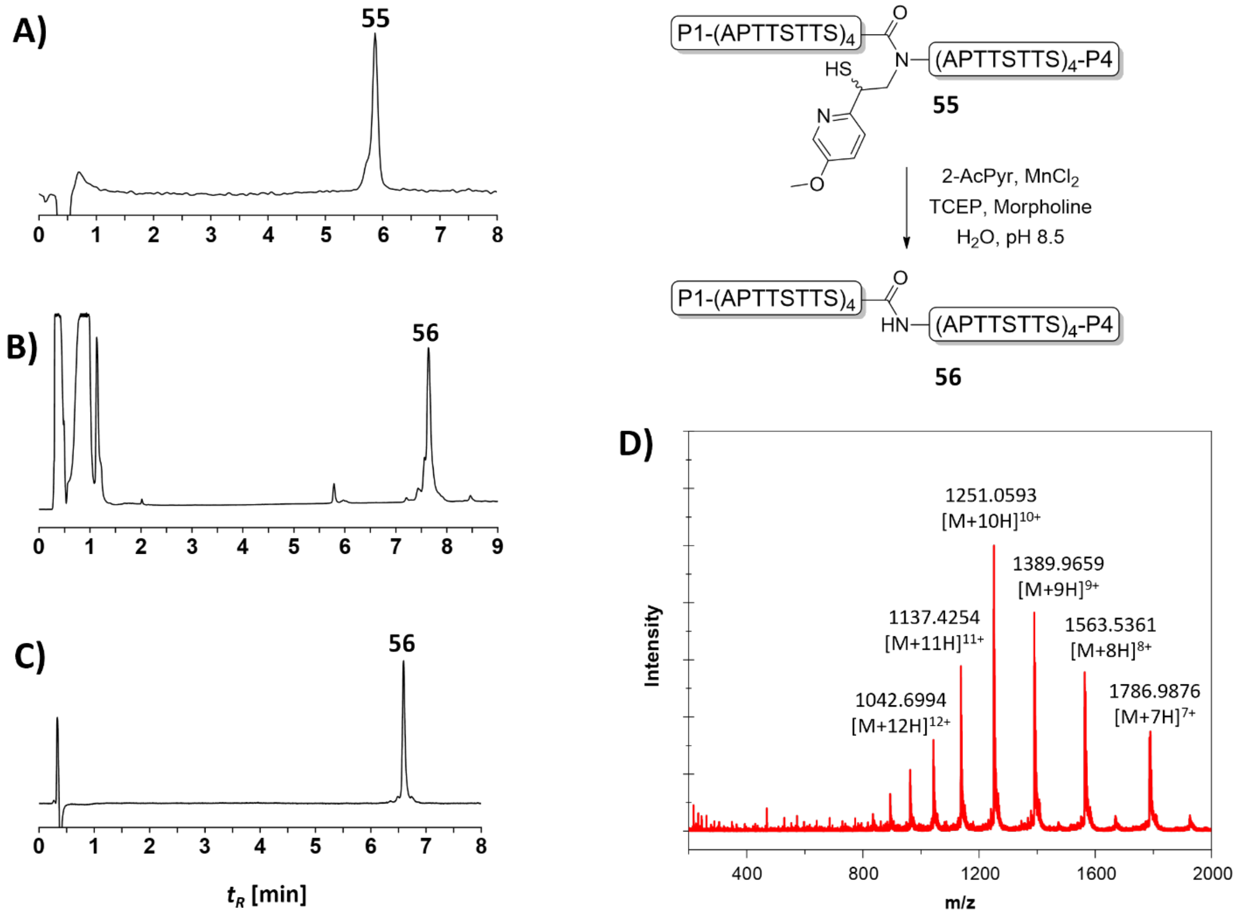

Figure S41: UPLC trace of A) purified starting material **55** and B) crude **56** obtained after 4 h incubation with the removal mixture. Purified **56** analyzed by C) UPLC and D) ESI-HRMS. Conditions: A), C) 15 - 35 % B in 8 min, λ = 210 nm; B) 15 % B in 1 min, then 15 - 35 % B in 8 min, λ = 210 nm.

11. Synthesis of His$_6$-Peg$_6$-(Y*p*SPTSPS)$_6$

His$_6$-[(CH$_2$)$_2$O]$_6$CH$_2$CO-(YXPTSPS)$_3$ Hydrazide [S15 (X = S), 58 (X = *p*S)]

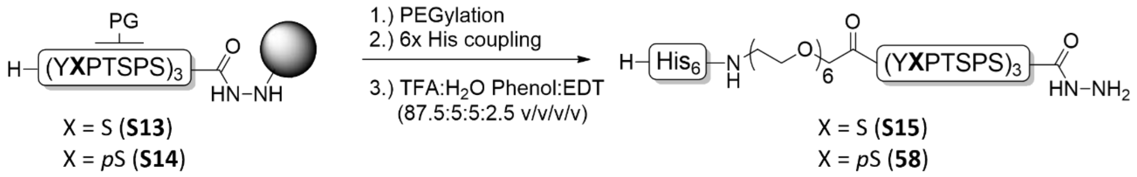

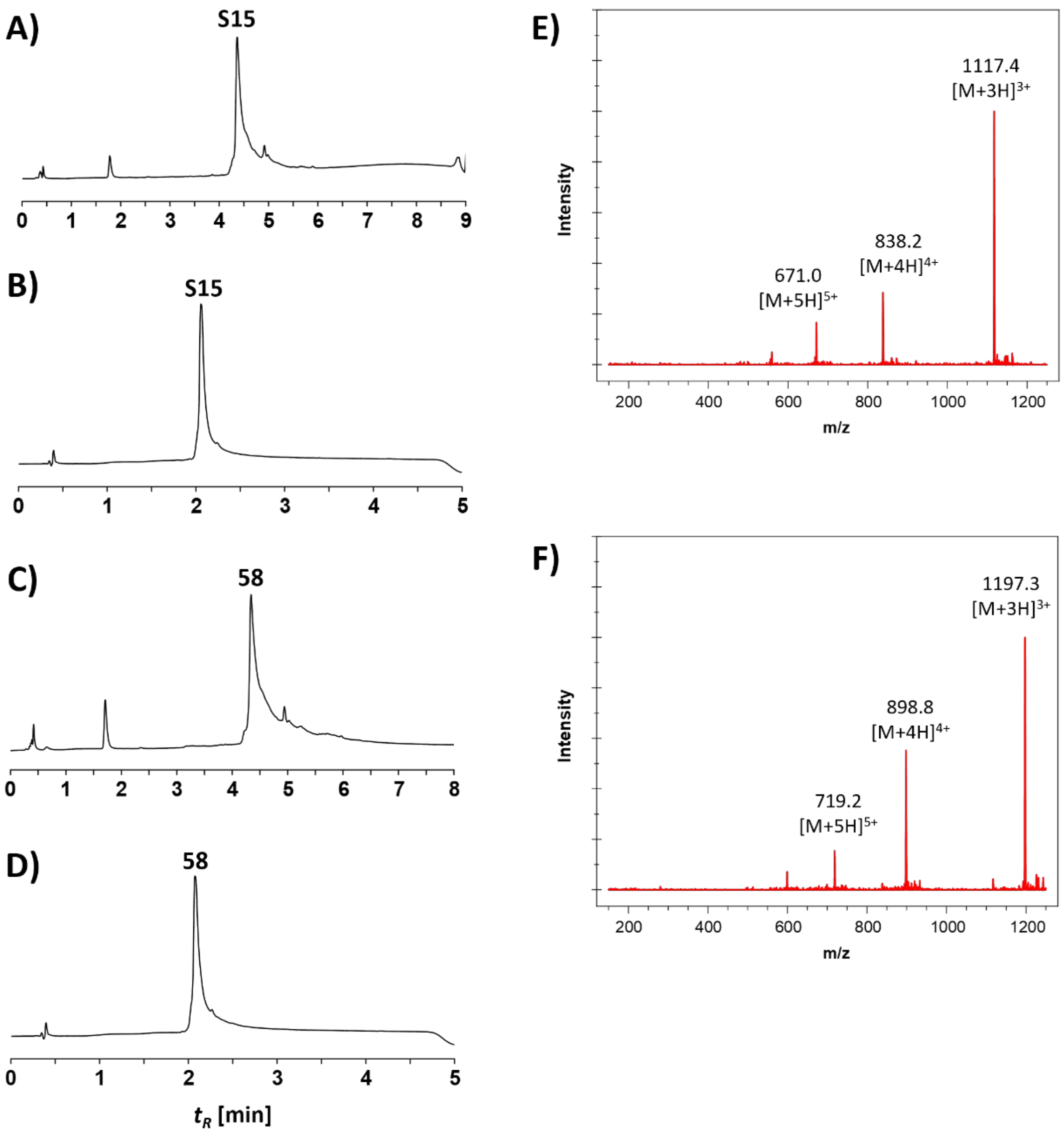

Figure S42: UPLC analysis of A) crude **S15** and C) crude **58** after SPPS, introduction of PEG$_6$-chain and six histidine couplings. Purified **S15** analyzed by B) UPLC and E) ESI-MS and purified **58** analyzed by D) UPLC and F) ESI-MS. Conditions: A), C) 3 - 30 % B in 8 min, λ = 210 nm; B), D) 3 - 30 % B in 4 min, λ = 210 nm. Tailing is commonly observed with peptide hydrazides.

Introduction of MMPyE-Auxiliary [S18 (X = S), 57 (X = pS)]

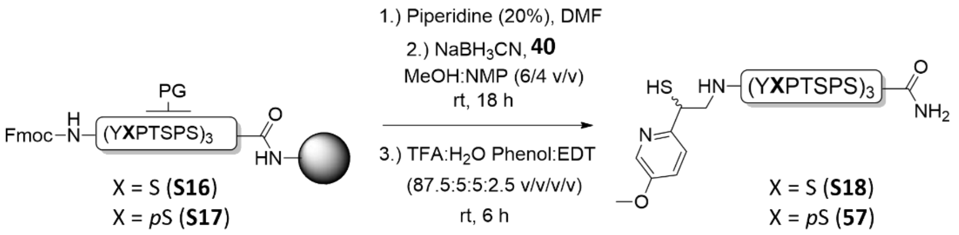

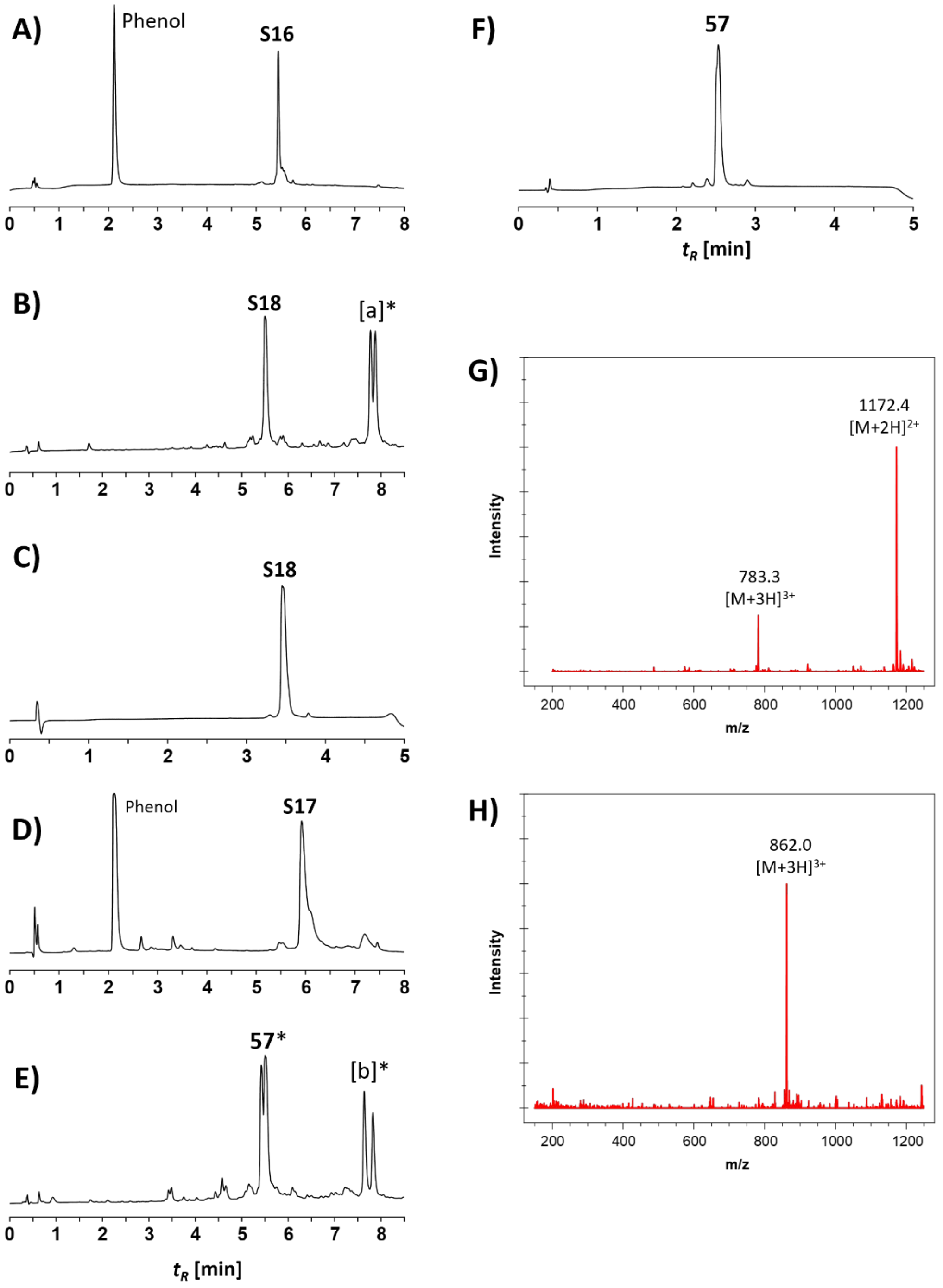

Figure S43: UPLC analysis of A) crude **S16** after SPPS before final Fmoc deprotection and B) after Fmoc-cleavage, 18 h of reductive alkylation and 6 h TFA cleavage and D) crude **S17** after SPPS before final Fmoc deprotection and E) after Fmoc-cleavage, 18 h of reductive alkylation and 6 h TFA cleavage. Purified **S18** analyzed by C) UPLC and G) ESI-MS and purified **57** analyzed by F) UPLC and H) ESI-MS. Conditions: A), D) 3 - 60 % B in 8 min, λ = 210 nm; B), E) 3 - 30 % B in 8 min, λ = 210 nm. C), F) 3 - 30 % B in 4 min, λ = 210 nm. [a] Tmb-protected **S18**. [b] Tmb-protected **57**. *Mixture of Stereoisomers.

Formation of Thioester S19, Ligation with S18 and Removal of MMPyE Group from S20 (S21)

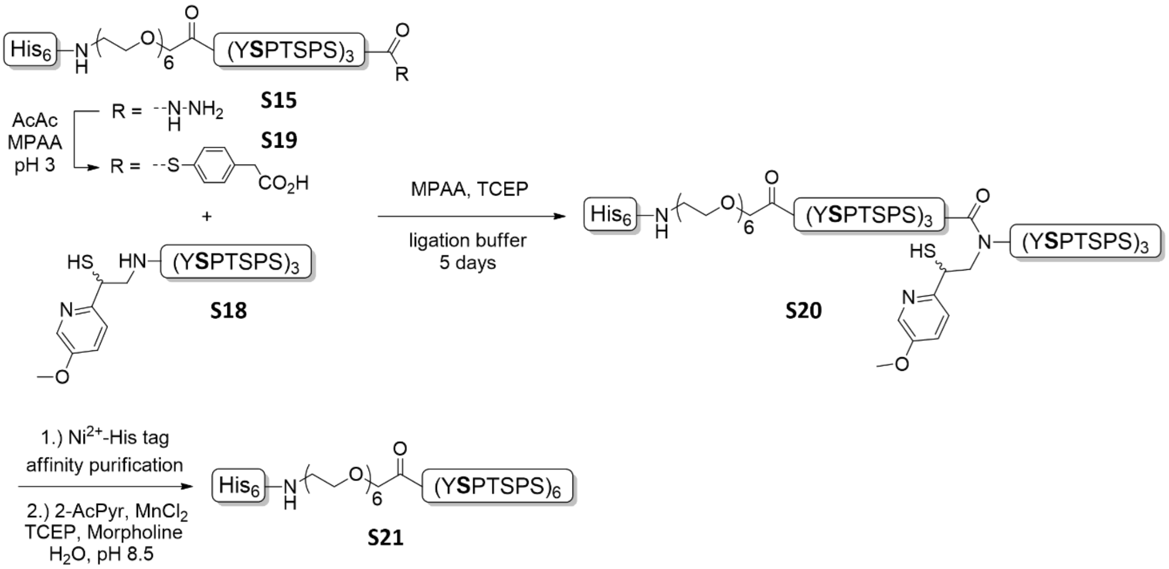

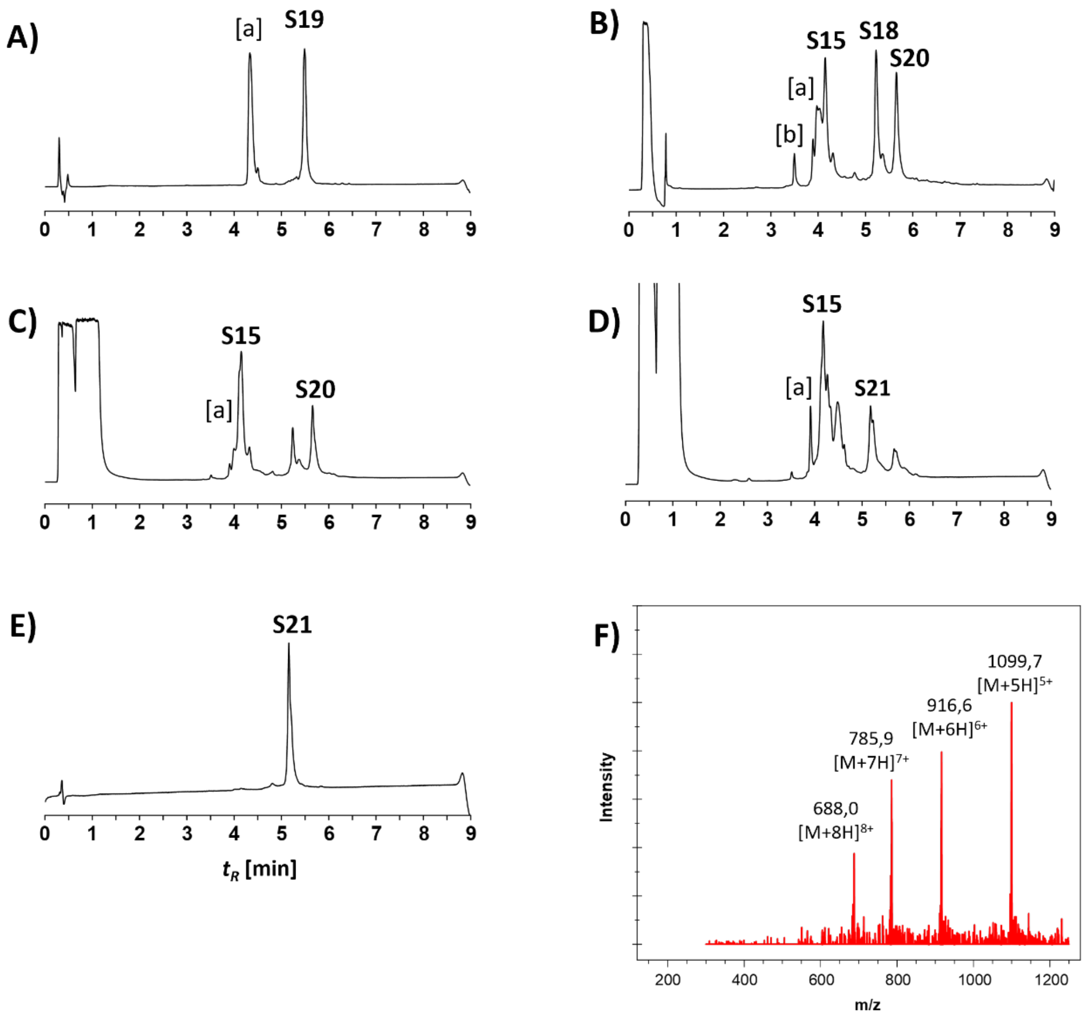

Figure S44: UPLC analysis of A) crude **S19** after treatment of **S15** with AcAc and MPAA (pH 3) and B) mixture obtained upon 5 days ligation of crude **S15** with purified **S18** and treatment with hydrazine (2.5 vol.%). UPLC analysis of **S20** after Amicon mass filtering and HisTag purification and exposure to removal conditions C) at t = 1 min and D) after t = 24 h. Purified **S21** analyzed by E) UPLC and F) ESI-MS. Conditions: A) 3 - 50 % B in 8 min, λ = 210 nm; B) - E) 5 - 30 % B in 8 min, λ = 210 nm; [a] hydrolysed **S19**. [b] **S18** with auxiliary and adjacent tyrosine cleaved.

Formation of Thioester S59, Ligation with 57 and Removal of MMPyE Group from S15 (60)

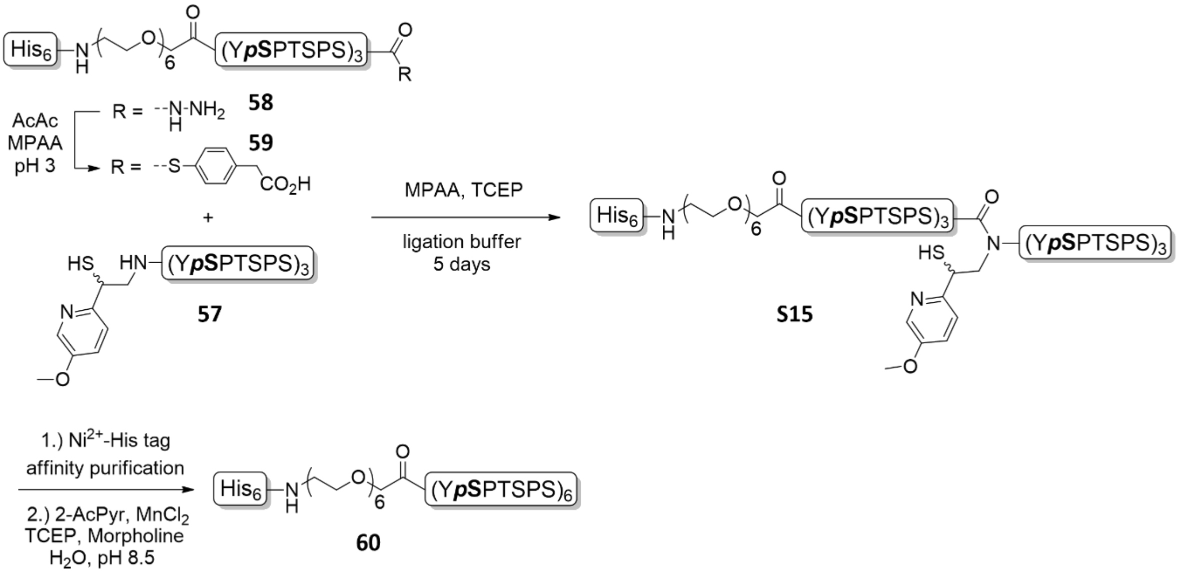

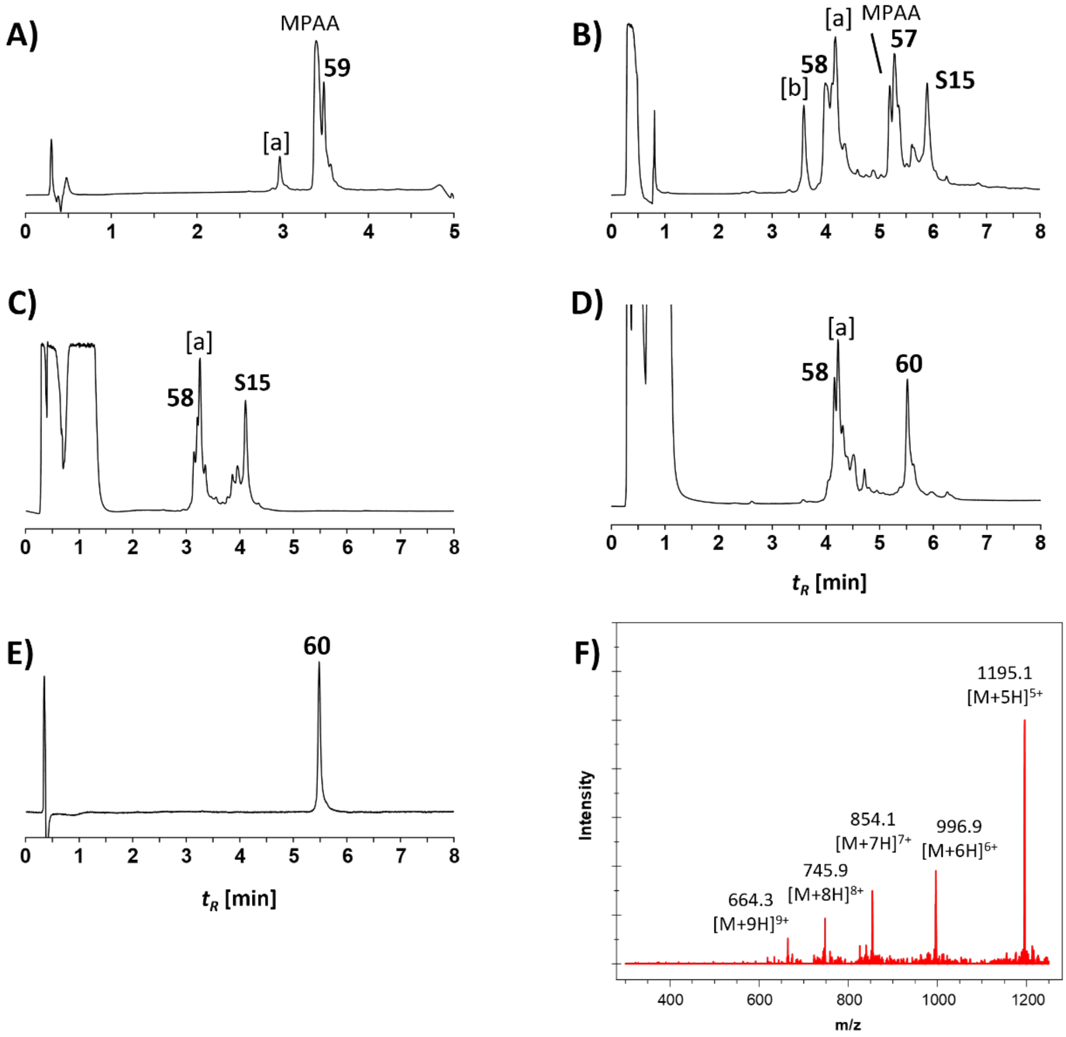

Figure S45: UPLC analysis of A) crude **59** after treatment of **58** with AcAc and MPAA (pH 3) and B) mixture obtained upon 5 days ligation of crude **59** with purified **57** and treatment with hydrazine (2.5 vol.%). UPLC analysis of **S15** after Amicon mass filtering and HisTag purification and exposure to removal conditions C) at t = 1 min and D) after t = 24 h. Purified **60** analyzed by E) UPLC and F) ESI-MS. Conditions: A) 3 - 50 % B in 8 min, λ = 210 nm; B) - E) 5 - 30 % B in 8 min, λ = 210 nm. [a] hydrolysed **59**. [b] **57** with auxiliary and adjacent tyrosine cleaved.

12. Synthesis of Cyclopeptide 65

Introduction of MMPyE-Auxiliary (62)

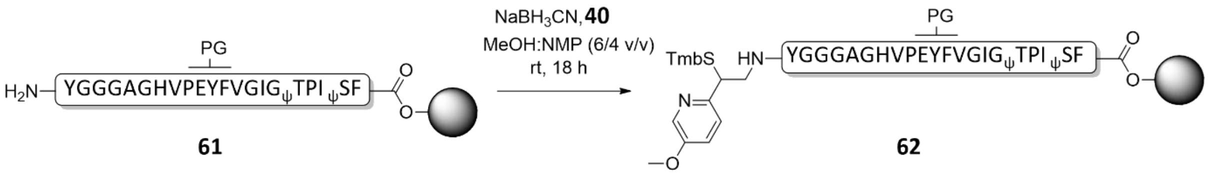

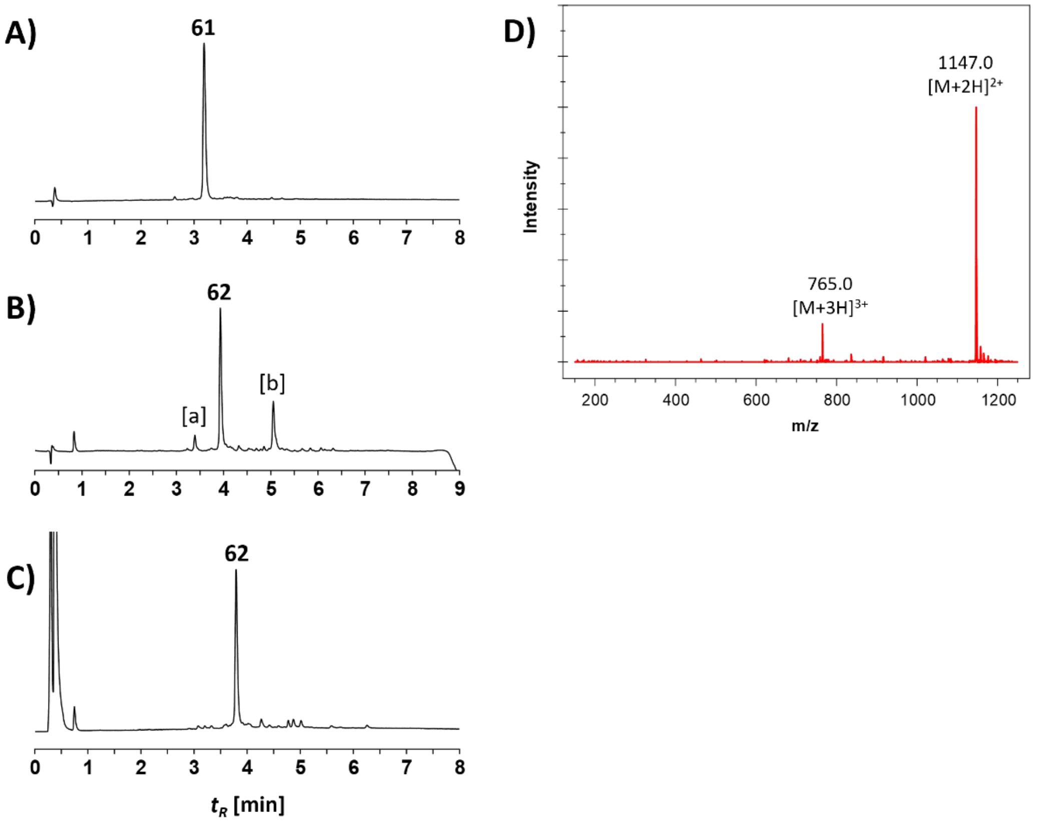

Figure S46: UPLC analysis of crude **61** A) before and B) after reductive alkylation and C) treatment with sat. $NaHCO_3$ to a pH of 7 (50 mM TCEP added). D) Crude MMPyE-peptide **62** analyzed by ESI-MS. Conditions: 20 - 50 % B in 8 min, λ = 210 nm. [a] disulfide of **62**. [b] **62** with a trifluoracetyl group added.

Formation of Thioester 58 and Cyclization (59)

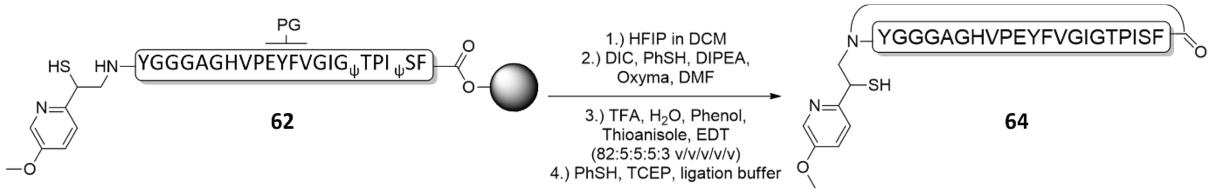

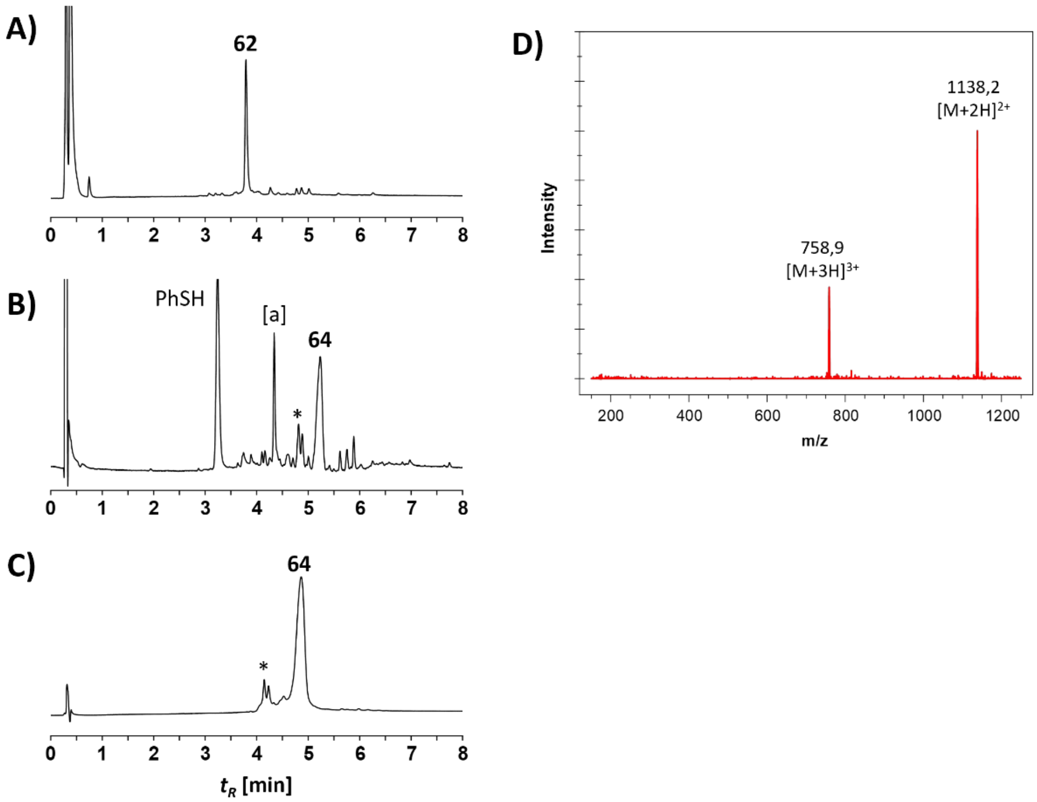

Figure S47: UPLC analysis of crude **62** A) before and B) after step 1.) - 4.). Purified cyclization product **64** analyzed by C) UPLC-trace and D) ESI-MS. UPLC analysis: 20 - 50 % B in 8 min, λ = 210 nm. [a] hydrolyzed thioester (from **63**). *indicates a peak with a m/z ratio equal to **64**. After auxiliary cleavage (see S49) the isomer peak disappears. We speculate that * is a cis/trans isomer of **64**.

Cyclization of Purified Thioester 63 (64)

For investigation of cyclization of purified thioester **63** was subjected to preparative HPLC, analyzed via UPLC (Fig. S48 A) and lyophilized over night. The white lyophilizate obtained after 18 h was analyzed by UPLC to determine complete conversion to cyclized peptide **64** (Fig. S48 B).

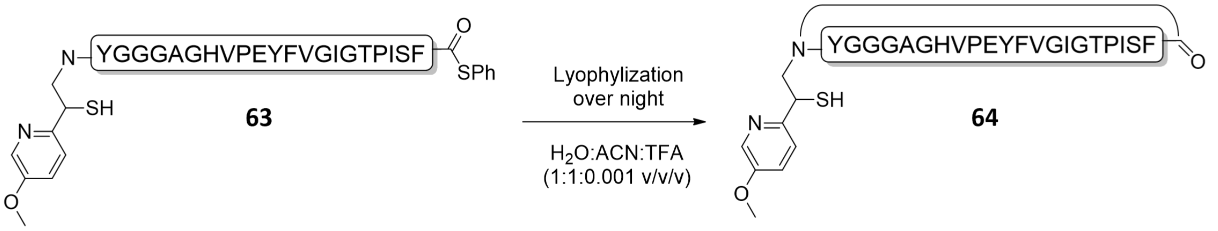

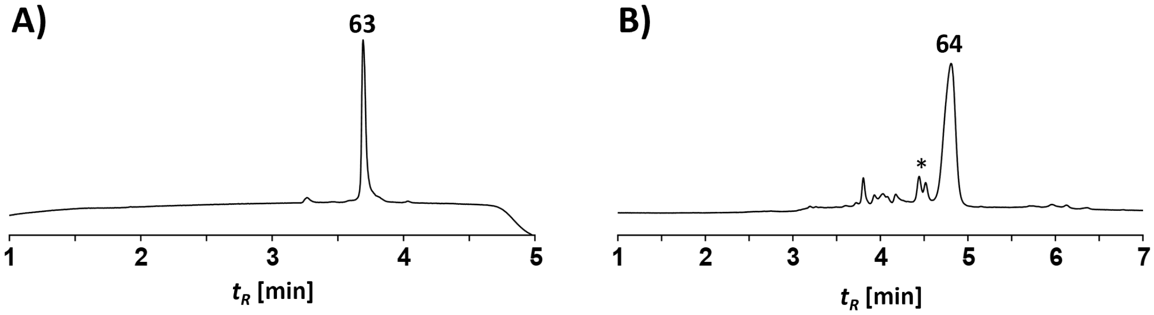

Figure S48: UPLC Analysis of A) thioester **63** immediately after HPLC purification and B) of cyclized peptide **64** obtained after 18 h of lyophilization.

Cyclopeptide 60

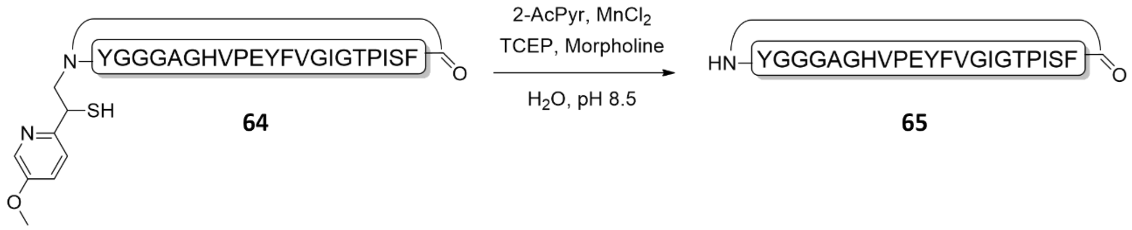

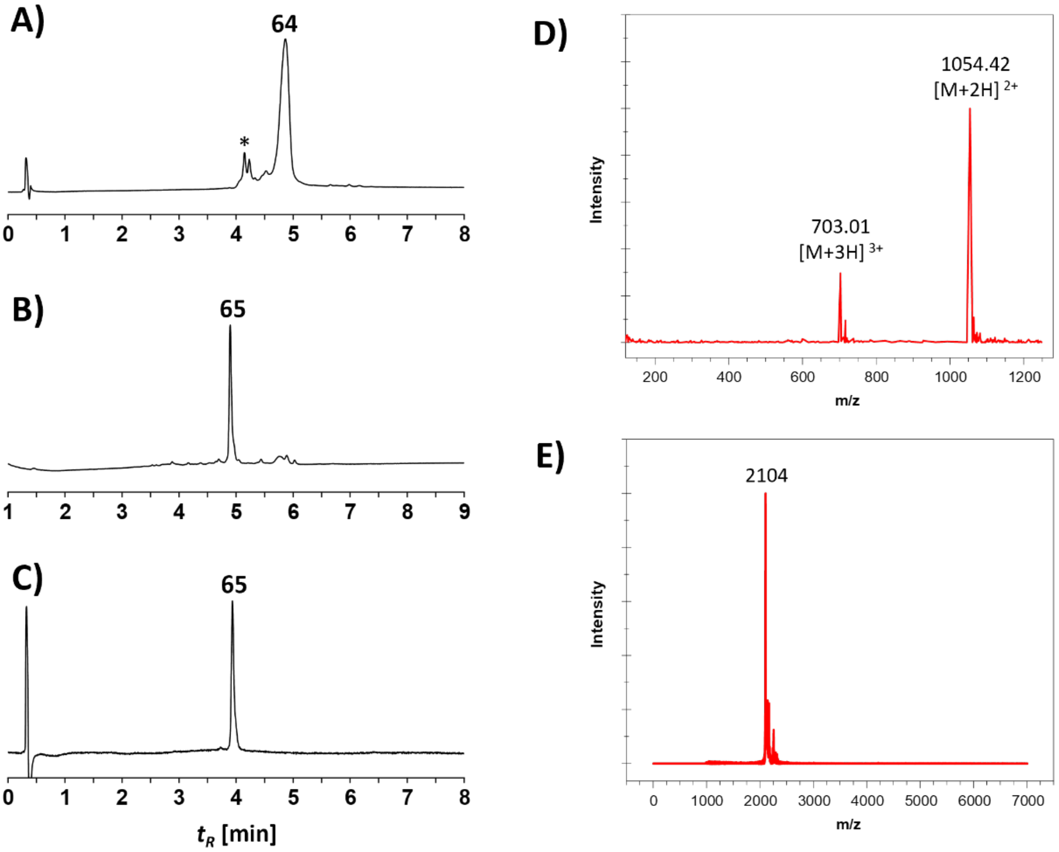

Figure S49: UPLC trace of A) starting material **64** and B) crude **65** obtained after 12 h incubation with the removal mixture. Purified **65** analyzed by C) UPLC, D) ESI-MS and E) MALDI-MS. UPLC analysis: A), C) 20 - 50 % B in 8 min, λ = 210 nm; B) 20 % B in 1 min, then 20 - 50 % B in 8 min, λ = 210 nm. *isomer of **64**.

13. References

[1] a) R. E. Thompson, B. Chan, L. Radom, K. A. Jolliffe and R. J. Payne, *Angewandte Chemie International Edition* **2013**, *52*, 9723-9727; b) S. Mavila, B. T. Worrell, H. R. Culver, T. M. Goldman, C. Wang, C.-H. Lim, D. W. Domaille, S. Pattanayak, M. K. McBride, C. B. Musgrave and C. N. Bowman, *Journal of the American Chemical Society* **2018**, *140*, 13594-13598.

[2] Y.-S. Tung, M. S. Coumar, Y.-S. Wu, H.-Y. Shiao, J.-Y. Chang, J.-P. Liou, P. Shukla, C.-W. Chang, C.-Y. Chang, C.-C. Kuo, T.-K. Yeh, C.-Y. Lin, J.-S. Wu, S.-Y. Wu, C.-C. Liao and H.-P. Hsieh, *Journal of Medicinal Chemistry* **2011**, *54*, 3076-3080.

[3] O. Fuchs, S. Trunschke, H. Hanebrink, M. Reimann and O. Seitz, *Angewandte Chemie International Edition* **2021**, *60*, 19483-19490.
